# Patterns of violence exposure in a life-course perspective and associations to mental and physical health problems and health-related risk behaviors among women and men in Sweden: A latent class analysis

**DOI:** 10.1016/j.ssmph.2025.101874

**Published:** 2025-10-23

**Authors:** Rickard Pettersson, Steven Lucas, Mattias Strandh

**Affiliations:** a Department of Social Work, Umeå University, Umeå, SE-901 87, Sweden; b Department of Women's and Children's Health, Uppsala University, Uppsala, SE-751 85, Sweden

**Keywords:** Violence, Adverse childhood experiences, Poly-victimization, Health, Life-course, Latent class analysis

## Abstract

**Background:**

Individual histories of abuse characteristics and other adversities must be considered to understand poly-victimization and its impact on ill-health, which suggests the importance of understanding how experiences of violence are interconnected over a life-course.

**Objective:**

To explore gendered patterns of lifetime poly-victimization—physical, emotional, and sexual—and examine how distinct exposure profiles relate to adult health outcomes. The analysis is guided by the Trauma-Informed Theory of Individual Health Behavior (TTB) framework to deepen understanding of gendered trauma trajectories and their long-term effects.

**Methods:**

10 337 Swedish women and men aged 18–74 participated in a combined online and postal survey. Attrition bias was controlled for based on official registry information. Latent Class Analysis (LCA) was used for identification of groups. Associations between mental and physical health indicators and health-related risk behaviors were analyzed using logistic regression, adjusting for background variables including age, self-reported parental immigrant status, and parental educational attainment.

**Results:**

Patterns of lifetime poly-victimization were more complex among women (7 classes) than men (4 classes). Among men, exposure was primarily characterized by childhood physical and emotional violence, as well as adult non-partner physical violence. In contrast, women's profiles often included childhood sexual violence and partner violence in adulthood, with stronger associations to multiple health problems and risk behaviors, and generally higher odds ratios compared to men. Among women, three unique clusters were identified, one of which may reflect more advanced resilience capacities compared to other clusters with similar trajectories of childhood violence exposure.

**Conclusions:**

Gendered patterns of poly-victimization and their health-related consequences underscore the importance of early intervention to prevent revictimization. The identification of unique and resilient clusters among women, despite similar childhood violence exposure, highlights the need for further research into protective mechanisms and trauma-to-benefit pathways, as conceptualized within the TTB framework.

## Highlights

• Patterns of exposure to poly-victimization in a life-course perspective are more complex among women compared to men.
• Women showed significant associations with a larger number of health problems and higher odd ratios compared to men.
• Associations to ill-health extend beyond the mere “dose” of victimization in childhood.
• Violence in adulthood amplifies the health correlates of childhood violence exposure.
• Resilient clusters highlights the need for research into protective mechanisms, as conceptualized within the TTB framework.

## 1 Introduction

Globally, exposure to interpersonal violence is common in a lifetime perspective, which poses a major threat to public health (Krug et al., 2002; WHO, 2014). The importance of multiple victimization over the life course is not well investigated (Simmons & Swahnberg, 2021) nor the associations between multiple victimization over the life-course and associations to long-term health problems and health related risk behaviors, especially in a Swedish context.

Research has shown that different types of violence are not evenly distributed in the population. Analyses, mainly from the United States (US) and the United Kingdom (UK), have shown that women are exposed to violence at higher rates than men (Walby & Allen, 2004; WHO, 2014), those exposed to one type of violence are at increased risk of other types of victimization (Finkelhor et al., 2009; Turner et al., 2010), and those who suffer from violence in childhood are disproportionately exposed to victimization in adult life (McIntyre & Widom, 2011; Widom et al., 2008). Previous research in Sweden shows that exposure to violence in childhood and adulthood, poly-victimization and revictimization follow the same patterns as in the US and the UK (Andersson et al., 2015; Annerbäck et al., 2012; Jernbro & Landberg, 2020; Källström et al., 2020; Simmons et al., 2015; Simmons & Swahnberg, 2021).

There is a growing body of empirical evidence that the accumulation of victimization is of importance for understanding well-being, where poorer health is seen among those who have experienced multiple forms of victimization (Felitti et al., 1998; Madigan et al., 2023). Finkelhor and colleagues (2007) introduced the concept of poly-victimization, emphasizing the cumulative impact of multiple victimizations on an individual's well-being. The theoretical framework suggests that the experience of diverse victimizations can have synergistic effects, exacerbating the negative consequences for physical and mental health. This has also been recognized in several studies of various Swedish populations. Victims of several types of violence show manifold more negative health outcomes compared to victims of single types of violence (Jernbro & Landberg, 2020; Källström et al., 2020; Simmons et al., 2015; Simmons & Swahnberg, 2021). Rather than viewing each type of victimization in isolation, poly-victimization encourages a holistic understanding of an individual's victimization history.

The concept of poly-victimization focuses not only on the “dose” of victimization, but also emphasizes the possible role of synergistic effects for well-being. This has also been recognized in a theory-based framework to understand and act on the mechanisms linking traumatic experiences and poor health outcomes (Marks et al., 2022). According to the Trauma-Informed Theory of Individual Health Behavior (TTB), childhood experiences of maltreatment may negatively affect the ability of some individuals to make positive choices regarding health-related behaviors but also to avoid environments that increase the likelihood of violence later in life.

Individual histories of abuse characteristics and other adversities must be considered to understand poly-victimization and its impact on ill-health, which suggests the importance of understanding how experiences of violence are interconnected over a life-course (Scott-Storey, 2011). Recent international research has started to examine empirically how exposure to violence and other traumatic events occurs in certain patterns, rather than randomly, using person-centered statistical techniques such as Latent Class Analysis (O'Donnell et al., 2017). Such systematic empirical approaches to violence and poly victimization specifically are rare and are currently lacking in Sweden. It is important to investigate whether exposure to different types of violence in childhood and adulthood may follow specific patterns and whether such patterns and their relationship with long-term health may vary between group membership.

### 1.1 Present study

This study aimed to analyze patterns of lifetime exposure to physical, emotional, and sexual violence in Sweden, to theoretically examine these patterns through the lens of TTB, and to investigate whether different exposure patterns are linked to varying risks of physical and mental health problems and health-related risk behaviors in adulthood. Potential disparities with respect to gender were also in focus. This was done using Latent Class Analysis (LCA) on a nationally representative Swedish survey of 10 337 women and men aged 18–74.

## 2 Background

### 2.1 Poly-victimization and health outcomes

Numerous studies in Sweden have concentrated on the phenomenon of poly-victimization across various age groups, encompassing children, adolescents, adults, and the elderly. A national survey investigating the experiences of violence among upper secondary school students aged 15 to 17 revealed a high prevalence of poly-victimization (Jernbro & Landberg, 2020). Among respondents who provided comprehensive answers regarding instances of child abuse, nearly 17 % reported exposure to two or more forms of violence. Notably, girls exhibited a higher frequency of poly-victimization compared to boys (11 % versus 5 %, respectively). One proposed explanation is that girls were significantly more prone to instances of sexual and psychological violence and were more likely to report parental violence compared to boys. The prevalence of poly-victimization varied across distinct forms of abuse. Among students who experienced neglect, 67 % were also exposed to other forms of violence, while corresponding percentages for emotional violence, physical violence, and violence between adults were 51 %, 32 %, and 48 %, respectively. The study also demonstrated a distinct dose-response pattern, indicating a correlation with mental and physical health problems, as well as health-related risk behaviors.

In a representative sample of young adults aged 20–24 (Källström et al., 2020), respondents answered questions regarding their encounters of physical, verbal or sexual violence, and property crime. Distinct victimization patterns, contingent on the perpetrator and gender, were identified; women were more prone to victimization by parents, siblings, and partners, while men faced a higher likelihood of victimization by peers. Peers emerged as the most frequent perpetrators of poly-victimization, with the aggression predominantly directed toward men. A positive dose-response relationship manifested in the context of victimization by peers, correlating with mental health issues such as depression, anxiety, and PTSD for both women and men. Furthermore, greater exposure to violence by parents and partners was linked to elevated levels of mental health problems among women, though no such correlation was evident for men.

Simmons and colleagues (2015) studied physical, emotional, and sexual violence exposure, along with victimization from multiple perpetrators (family, partner, acquaintance/stranger) in two adult samples: a population (18–64) and a clinical sample (18–91). In both women and men across population and clinical groups, poly-victimization and exposure to violence from multiple perpetrators were more strongly linked to mental health problems (anxiety, insomnia, and PTSD in a lifetime perspective measured as a sum score categorized as “few symptoms” and "many symptoms”) than single exposures or violence from one perpetrator. For instance, women reporting all three forms of violence were four times more likely to exhibit many mental health symptoms than those reporting only one form. Men with two or three kinds of perpetrators were three times more likely to report many mental health symptoms than those with one perpetrator. Additionally, exposure to emotional violence and any partner victimization, or any combinations of emotional/partner violence, posed the highest risks for many symptoms in both genders.

In a follow-up study, Simmons and Swahnberg (2021) examined an elderly subsample (ages 60–85) from the previous population study (Simmons et al., 2015). Their aim was to explore the lifetime prevalence of physical, emotional, and sexual violence, and the occurrence of "high" and "low" poly-victimization from various perpetrators. High and low poly-victimization was based on sum score (number of forms of violence, perpetrators and duration/frequency of violence exposure), with a cut-off score chosen for high poly-victimization so that approximately one-third of the poly-victims were classified as high poly-victims. They also investigated associations with general physical and mental health and the effect of resilience factors (sense of coherence and social support). About 24.8 % of women and 27.6 % of men reported some type of lifetime victimization, and of these 82.1 % of women and 62.4 % of men reported poly-victimization. Low poly-victimization was more prevalent among women (56.7 %) than men (39.8 %, p = 0.04). High poly-victimization was equally distributed between women and men victims (25.4 % versus 22.6 %, p = 0.68). High and low poly-victims exhibited stronger correlations with health problems compared to single-exposure victims.

Although prior research among children and adults in Sweden thus indicates a high prevalence of poly-victimization, posing an elevated risk for adverse health outcomes compared to singular exposures to violence or individual perpetrators, our knowledge of poly-victimization remains incomplete and largely fragmented. Notably, analyses often focus on specific groups, life stages, or relatively small sample sizes and there is a need to expand health outcomes studied to long-term physical health problems and health-related risk behaviors, as well as specific mental health problems in adulthood. Analyses in the US and the UK have shown strong associations between the “dose” of adversities such as exposure to different types of violence in childhood and mental health problems (Chapman et al., 2004; Dube et al., 2001; Edwards et al., 2003; Petruccelli et al., 2019) and health-related risk behaviors (Anda et al., 1999, 2002; Bellis et al., 2014; Dube et al., 2002; Dube et al., 2003a; Petruccelli et al., 2019) in adulthood, but also associations to long-term physical health problems such as ischemic heart disease (IHD), type 2 diabetes and cancer (Bellis et al., 2015; Brown et al., 2010; Dong et al., 2004; Dube et al., 2003b; Godoy et al., 2021; Holman et al., 2016; Petruccelli et al., 2019; Williamson et al., 2002; Zhu et al., 2022).

### 2.2 Trauma-informed theory of individual health behavior (TTB)

Crucially, the concept of poly-victimization extends beyond mere quantification of victimization instances. By highlighting potential synergistic effects on well-being, it underscores the importance of comprehending how experiences of violence interconnect over the life-course to elucidate its relationship with health and overall well-being (Scott-Storey, 2011). To comprehend the heightened risk of exposure to poly-victimization across the lifespan, it is essential to adopt a theoretical framework that captures the complex interplay between traumatic experiences and long-term health outcomes. Recently, Marks et al. (2022) proposed an extension of Bronfenbrenner's social-ecological model, offering a theory-based framework to better understand and address the mechanisms linking trauma to adverse health effects. The Trauma-Informed Theory of Individual Health Behavior (TTB) posits that an individual's ability to make decisions that enhance the likelihood of positive health outcomes following exposure to childhood trauma is based on three factors: (1) the forms and severity of trauma they have been and are exposed to; (2) how this trauma physiologically manifests (i.e., the trauma response); and (3) resilience to undertake behavior change despite this trauma response. Accordingly, childhood experiences of violence may potentially influence an individual's ability to make constructive life choices and to avoid environments that could increase the risk of future exposure to violence, including a heightened vulnerability to poly-victimization and revictimization later in life. The TTB framework may also be used to explain the findings reported by others who have examined the mechanisms behind exposure to poly-victimization and revictimization.

### 2.3 Latent class analysis and exposure to violence

In international research, there is an expanding body of empirical analyses aiming to identify patterns of traumatic events, investigating how exposure to trauma follows discernible patterns rather than random accumulation. A pivotal aspect of these empirical approaches has been the utilization of person-centered statistical techniques, notably Latent Class Analysis (O'Donnell et al., 2017), as opposed to a variable-centered approach. The methodology enables, for our purposes, the examination of multiple dimensions of violence exposure involving diverse perpetrators and analyzes how these dimensions typically cluster within individuals. This contrasts with the conventional approach of merely assessing how different types of violence accumulate or correlate with one another. Such an approach may allow for an understanding of poly-victimization from the perspective of what typically is experienced, as well as for the analysis of the implications of these experiences which goes beyond the question “how much violence?” to also take into account the question of “when did the violence occur?” and “who was the perpetrator?”.

As mentioned above, the majority of studies utilizing Latent Class Analysis have been conducted to discern patterns of traumatic events, encompassing a broader range of traumas. However, in the past 10–15 years, some studies have employed this technique with a specific focus on violence. For instance, a study in Uganda (Clark et al., 2016) examined patterns of exposure to various forms of violence and perpetrators in a population of children. Three distinct classes of violence were identified, revealing that exposure to violence among children follows distinctive patterns clustered by perpetrator and setting. Several North American studies have concentrated on patterns of peer and dating violence, exploring negative health outcomes. These studies identified 3–6 homogeneous subgroups among boys and girls or within an all-female population (Choi et al., 2017; Garthe et al., 2021; Spencer et al., 2016). The findings of these studies underscore the significance of comprehending how peer and dating violence co-occur. Moreover, they shed light on how different victimization patterns are linked to internalizing symptoms, with adolescents experiencing multiple types of dating violence reporting greater mental health problems.

Of particular interest for our purposes within this group of studies is the study by Ansara and Hinden (2010), which conducted separate analyses for women and men. In this study Latent Class Analysis was used to map the patterns of physical violence, sexual coercion, psychological abuse and controlling behavior. Data from the 2004 Canadian General Social Survey were analyzed, which included a large sample of 8360 women and 7056 men 15 years of age and over who reported having a current or ex-spouse or common-law partner. The study found substantially more classes among women than men, indicating greater differentiation and severity of violence in certain female patterns of violence.

However, the study by Ansara and Hinden (2010) is an exception within this research field. Investigation of patterns of exposure to violence in adulthood through Latent Class Analysis has frequently focused on specific groups of women. For instance, Cavanaugh et al. (2012) explored the lifelong exposure to violence from various perpetrators among working nurses and identified a four-class model: 1. low violence, 2. high psychological and physical intimate partner violence, 3. high physical and psychological workplace violence, and 4. moderate to high childhood abuse. In comparison to class 1, membership in classes 2 and 4 was associated with screening positive for depression, and class 2 additionally showed a correlation with posttraumatic stress disorder. In a subsequent study focusing on women with lifetime histories of intimate partner violence, Cavanaugh et al. (2013) identified a three-class model linked to mental disorders such as PTSD and drug use disorder. Another study investigated the lifetime experience of violence from different perpetrators among a population of women attending an STD clinic and found that combinations of violence experiences were associated with sexual risk behaviors (Walsh et al., 2012).

Men are typically excluded from these studies in terms of their role in perpetrator patterns (Lee et al., 2021). An exception is Scott-Storey and colleagues (2023), who explored patterns of cumulative lifetime violence as both targets and perpetrators, as well as their associations with mental health problems in a community sample of Canadian men aged 19–65 years. They identified a four-class model that was related to depression and PTSD scores.

International, primarily North American, research utilizing person-centered techniques to empirically investigate patterns of violence has proven to be fruitful. It has uncovered interpretable patterns of violence exposure with significant explanatory power concerning health and behavior. However, despite a growing interest in the topic, there is a notable dearth of large, representative studies including both women and men that adopt a lifetime perspective. The application of trauma theory-based frameworks in examining whether distinct patterns of exposure are associated with varying risks of physical and mental health problems, as well as health-related risk behaviors in adulthood, appears to be limited. This study aims to address this knowledge gap.

## 3 Methods

The data used in the study are based on a questionnaire drawing inspiration from the Adverse Childhood Experiences (ACE) study (Felitti et al., 1998) and a previous national violence prevalence study in Sweden (Lundgren, 2002). The survey comprised sections on demographic information followed by retrospective inquiries about childhood experiences of physical and emotional neglect and parental problems including substance abuse, mental illness, and suicide attempts. Instances of sexual, physical, and psychological violence were probed separately for age brackets 0–15, 15–17, and after 18. The questionnaire underwent evaluation for face and content validity at Statistics Sweden, involving expert reviews and cognitive interviews with individuals with and without a history of violence exposure.

Following adjustments, the final Violence and Health survey instrument comprised 97 questions with over 300 sub-items (Andersson et al., 2015). The questionnaire was distributed to a random sample of 10 000 women and 10 000 men aged 18 to 74 selected randomly from the Swedish population registry. An opt-out approach was applied. Subsequent letters provided instructions for online completion of a web-based survey, with paper questionnaires sent to non-responders. A maximum of four reminders were issued. Respondents were informed that completing the questionnaire constituted informed consent.

From the original sample of 20 000, 10 337 completed the questionnaire, resulting in a 52 % response rate (56 % for women, 48 % for men). Non-responder analysis and a multifactorial weighting algorithm addressed sociodemographically underrepresented groups. This research has been conducted in accordance to the Declaration of Helsinki, and the study was approved by the Ethical Review Board in Uppsala (Dnr 2011/156).

### 3.1 Measures

The variables included in the analyses were dichotomous or categorical with mutually exclusive groups.

#### 3.1.1 Indicators of exposure to violence

Thirteen variables were constructed to investigate patterns based on different perpetrators of physical, emotional and sexual violence in a life-course perspective. The variables were dichotomized, partly because there is a basic boundary between being exposed to violence and not, but also in order to manage the number of variables. For each violence type, any response indicating violence exposure was counted as a positive outcome.

#### 3.1.2 Exposure to violence in childhood, before the age of 18

Physical violence by a parent and other adults/peers (relatives, teachers, coaches or other adults/relatives, friends or other same age persons): Threatened you with a beating; hit you with open hand (a slap), pulled your hair, pushed you in a way that was painful; hit you with fist or a hard object, kicked you, took you in a chokehold; harmed you using a knife or firearm; subjected you to another kind of physical violence.

Emotional violence by a parent and other adults/peers: Violated or oppressed you verbally (for example degraded, insulted or humiliated you).

Sexual violence by a parent and other adults/peers: Forced you to pose naked; touched or caressed you in a sexual way; made you touch him/her in a sexual way; tried to have intercourse with you (oral, vaginal or anal) but did not complete the act; had intercourse with you (oral, vaginal or anal).

Physical, emotional and/or sexual violence by a partner: Threatened you with or subjected you to physical violence; offended or verbally oppressed you; forced you to pose naked; touched or caressed you in a sexual way; made you touch him/her in a sexual way; tried to have intercourse with you (oral, vaginal or anal) but did not complete the act; had intercourse with you (oral, vaginal or anal).

#### 3.1.3 Exposure to violence in adulthood, after the age of 18

Physical violence by a partner and other adults (relatives, acquaintances, co-workers, clients/patients, strangers): Threatened you with physical violence; hit you with open hand (a slap), pulled your hair, pushed you, etc.; hit you with a fist or a hard object, kicked you, took you in a chokehold, etc.; harmed you using a knife or firearm; subjected you to another kind of physical violence.

Emotional violence by a partner and other adults: Systematically and repeatedly degraded, insulted, humiliated or otherwise violated your dignity or oppressed you verbally; systematically and repeatedly dominated you and decided who to meet, how much money you were allowed to have, when to go out, what clothes to wear, etc.; systematically and repeatedly threatened to hurt him/herself or your children, to take the children and leave, to break valuables, to tell others things you would like to keep secret, etc.; systematically and repeatedly bullied, violated or harassed you at your workplace, in school, in your residential area, etc.

Sexual violence by a partner and other adults: Forced you to have sexual intercourse (oral, vaginal, anal) or another similar sexual act (such as masturbation) by using or threatening physical violence; attempted to force you to have sexual intercourse (oral, vaginal, anal) or another similar sexual act (such as masturbation) by using or threatening physical violence; forced you, or attempted to force you to engage in some kind of sexual activity when you were unable to defend yourself because you were sleeping, ill or under the influence of alcohol or drugs; grabbed or touched you in a sexual way against your will (e.g. caressed, restrained, hugged, kissed or “groped” you) or made you touch his/her body in a sexual way against your will.

#### 3.1.4 Outcome variables

Current physical and psychological health was assessed using a number of previously validated instruments, as well as by self-report. In all, 15 outcome variables indicating mental health problems, physical health problems and health-related risk behavior were used. As many variables were naturally dichotomous the choice was made to dichotomize all of the variables. The variables and cut off points are presented in Table 1.

**Table 1:** Description of outcome variables, measures used and criteria for positivity.

Outcome variables		
	Measure	Criterion for positivity
**Mental health problems**		
Depression	Hospital Anxiety and Depression Scale (Herrmann, 1997)	11 points or above, representing probable depression (maximum 21 points)
Anxiety	Hospital Anxiety and Depression Scale	11 points or above, representing probable anxiety (maximum 21 points)
Post-traumatic stress symptoms (PTSS)	PCL (Ruggiero et al., 2003)	9 points (maximum 18 points)
Self-harm	Self report	Any positive reponse regarding physical self-harm, suicidal ideation or suicide attempts
Somatization	PHQ-15 (Kroenke et al., 2002)	10 points or above (maximum 24)

**Physical health problems**		

Irritable bowel syndrome (IBS)	Self report	Any positive response
Fibromyalgia	Self report	Any positive response
Ischemic heart disease (IHD)	Self report	Any positive response regarding heart attack, angina or heart failure
Chronic obstructive pulmonary disease (COPD)	Self report	Any positive response
Type 2 diabetes	Self report	Any positive response
Cancer	Self report	Any positive response
Obesity	Calculated BMI	BMI 30 or above

**Health-related risk behaviors**		

Heavy smoking	Self report	Smoking more than 20 cigarettes per day
Alcohol misuse	AUDIT (Bergman & Källmén, 2002).	8 points or above for men, 6 points or above for women
Drug abuse	Self report	Any positive response

#### 3.1.5 Background variables

The background variables included in the analyses were age (collected from registry data and categorized in groups aged 17–25/26–35/36–45/46–55/56–65/66–74) and self-reported parental immigrant status (at least one parent born in a Nordic country/both parents born elsewhere) and parents’ highest level of education (at least one parent with secondary school education/both parents below secondary school).

### 3.2 Data analysis

The main aim of the article was to identify different groups of lifetime exposure to violence and to investigate the associations to mental and physical health problems and health-related risk behaviors in adulthood. To do this, we applied Latent Class Analysis (LCA) using the software Latent GOLD 6.0 to the data on lifetime exposure to violence. As our previous research using the same dataset showed substantial differences in exposure to different violence types and polyvictimization burden between women and men, we decided to perform separate LCA analyses for women and men, respectively (Pettersson et al., 2025). Latent Class Analysis is a data driven approach that allows the discovery of relatively homogenous groups in a heterogenous population without having pre-defined classes. This approach stems from a theoretical framework that views individuals as belonging to unobserved (latent) classes, with membership influenced by patterns of indicators (Weller et al., 2020). The method allows for the identification of distinct subgroups within the population based on their patterns of exposure to violence throughout their lifetime.

The number of latent classes in the model is a critical aspect of LCA. This was determined after fitting models iteratively until goodness of fit indices indicated the most parsimonious solution. The Bayesian Information Criterion (BIC) and Akaike Information Criterion (AIC) were used to determine the optimal class solution by balancing model fit and complexity. Lower BIC and AIC values indicate a more parsimonious model, facilitating model comparison and choice. Both criteria estimate prediction error while penalizing excessive parameters to prevent overfitting, with BIC applying a stronger penalty for complexity (Nylund et al., 2007; Weller et al., 2020). Given the large sample size in this study (n = 10 337), BIC was prioritized given that it penalizes complexity in the form of larger sample sizes more. The quality of classification in the chosen model was assessed using entropy, where values exceeding 0.6 were considered acceptable (Weller et al., 2020). While fit statistics play a role, it is acknowledged that a theoretically justified or more interpretable model may sometimes be preferred over one with better fit statistics (Porcu & Giambona, 2017). This means that models also must be evaluated for conceptual meaningfulness.

Additionally, our study incorporated a two-fold split-sample cross-validation procedure, following the methodology outlined by Masyn (2013). This validation process involved a random split of the sample, fitting the LCA model to one subset and validating it on the other, and vice versa. The stability and replicability of the optimal class solution across the two split samples establish the robustness of the findings.

The 15 outcome variables were dichotomous indicators of health issues. We examined the association between the groups identified in the LCA analysis of lifelong exposure to violence and the indicators of mental and physical health and health-related risk behaviors through binary logistic regression analyses. An analysis of binomial correlations demonstrated only weak associations (Pearson's r < 0.4) between background variables and the exposure and outcome variables. Nonetheless, as these background variables may be expected to affect the correlations between the exposure and outcome variables based on previous research, they were used in the regression analyses to adjust for potential confounding effects (Armstead et al., 2021; McLaughlin et al., 2016). First, unadjusted analyses were performed using each health outcome as the dependent variable and a single categorical variable with mutually exclusive groups for LCA class affiliation among women and men, respectively, as the independent variable. In the next step, the background variables were added as covariates in a single block for the adjusted model. As the adjusted model showed only minor differences in odds ratios and no shifts between significance and non-significance, only results from the adjusted model are presented in the results section. For each regression analysis, cases with missing data were excluded. The detailed results of the regression analyses are presented as Appendix 1. The data underwent analysis using SPSS version 28 by IBM Corp. A significance level of p < 0.05 and two-tailed analyses were applied.

## 4 Results

**Fig. 1:**
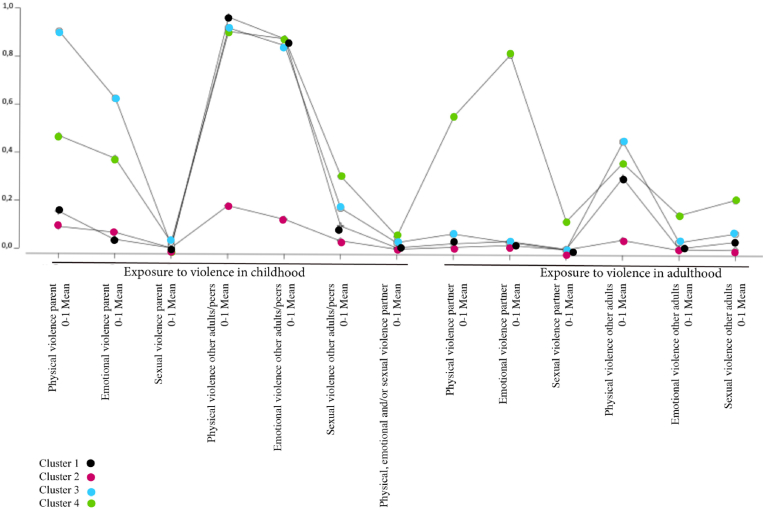
Men, plot of mean likelihood for exposure to violence for four classes (mean value for respective cluster).

In order to investigate if there are empirically discernible patterns of lifetime exposure to violence and if these groups differ between men and women we split the samples on gender before applying Latent Class Analysis, the result for men can be seen in Table 2 and the result for women can be seen in Table 5. In the process of model selection many models were attempted and models that did not have acceptable fit criteria were not selected. Table 2 shows that the analysis for men indicated a four cluster model being the best fit using the applied criteria. Although the AIC was somewhat lower for a five-cluster model, the BIC was lowest for the four-cluster model, which should be prioritized given the size of the sample. The four-cluster model also has acceptable entropy. Fig. 1 illustrates the four cluster model regarding exposure to violence in a life-course perspective for men plotted against mean values for the violence variables. Table 3 demonstrates the likelihood of violence exposure among men according to cluster with respect to perpetrator type in childhood and adulthood.

**Table 2:** Men, indicators of fit for models with one through five latent classes.

Classes	LL	BIC(LL)	AIC(LL)	L^2^	df	p-value	Entropy R^2^
1	−16791,7	33691,9	33609,4	5115,4	4209	1,1e-20	1,00
2	−15320,5	30866,4	30695,0	2173,0	4195	1,00	0,74
3	−14959,5	30261,4	30001,1	1451,1	4181	1,00	0,68
4^a^	−14801,4	30062,0	29712,9	1134,9	4167	1,00	0,72
5	−14748,3	30072,7	29634,7	1028,6	4153	1,00	0,71

a Best fitting model according to criterion.

**Table 3:**
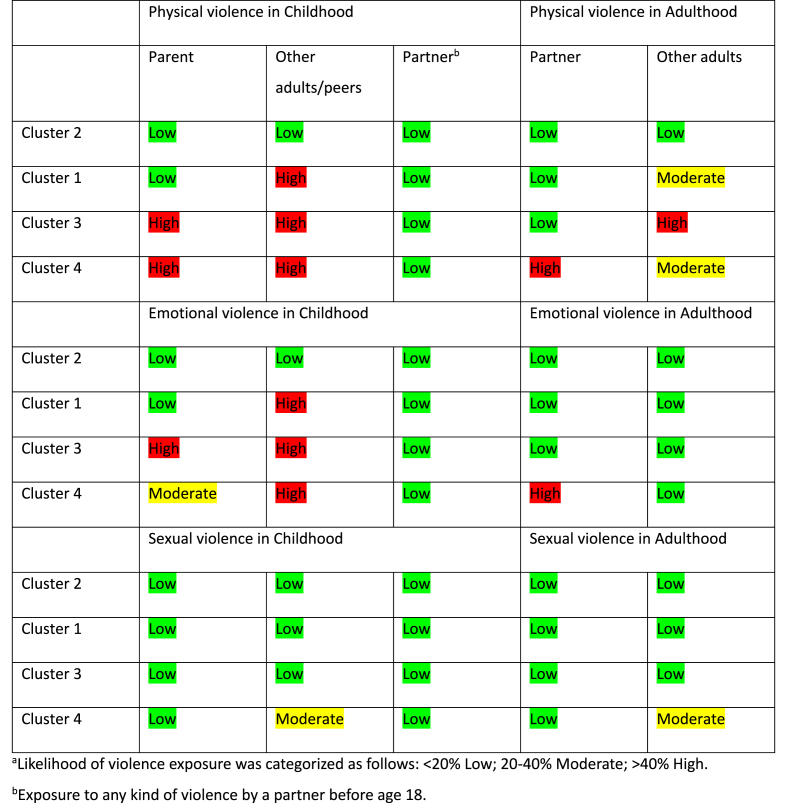
Likelihood^a^ of violence exposure among men according to cluster with respect to perpetrator type in childhood and adulthood.

Cluster 2 represented 43.1 % of the entire sample. This group showed low likelihood of exposure to all types of violence in both childhood and adulthood and was therefore used as the reference group in the logistic regression analyses shown further below.

Cluster 1 included 43 % of the sample. In this group, exposure to physical and emotional violence by other adults/peers in childhood was highly likely, and physical violence from other adults in adulthood was moderately likely.

Cluster 3 comprised 8.1 % of the sample. This group was characterized by a high likelihood of exposure to physical and emotional violence from a parent and other adults/peers during childhood and a high likelihood of physical violence from other adults in adulthood.

Cluster 4 included 5.9 % of the sample and was the group that showed the highest multiplicity of violence exposure. The group had high likelihood of physical violence, and moderate to high likelihood of emotional violence by parent and other adults/peers during childhood. Additionally, the group had moderate likelihood of sexual violence by other adults/peers before the age of 18. In adult life, the group showed high likelihood of exposure to physical and emotional violence by a partner and moderate likelihood of sexual violence by other adults.

There were clear associations between cluster membership and mental health problems among men, with higher odds of ill health coupled with higher multiplicity of exposure to violence in both childhood and adulthood (Table 4). The same pattern of associations was seen for somatization but not for the other physical health conditions studied. Significant associations were observed between clusters 1, 3 and 4 and IBS, while only cluster 4 was significantly associated with COPD and only cluster 3 with cancer. Heavy smoking and alcohol misuse were associated with clusters 1, 3 and 4 compared to the non-victimized group (cluster 2). A significant association with drug abuse was found for cluster 4.

**Table 4:** Cluster membership and associations with health problems and health-related risk behaviors among men, logistic regression models (odds ratios with 95 % confidence intervals).

	Depression		Anxiety		PTSS		Self-harm	
	OR	95 % CI	OR	95 % CI	OR	95 % CI	OR	95 % CI
Cluster membership^a^								
Cluster 1	1.51∗∗	1.13–2.03	1.44	0.85–2.45	1.65∗	1.07–2.53	3.48∗∗∗	2.39–5.05
Cluster 3	2.47∗∗∗	1.62–3.76	4.26∗∗∗	2.28–7.94	4.40∗∗∗	2.60–7.44	10.46∗∗∗	6.74–16.24
Cluster 4	3.11∗∗∗	1.69–3.85	6.56∗∗∗	3.48–12.36	9.24∗∗∗	5.52–15.45	16.48∗∗∗	10.46–25.97
*Pseudo R-square*								
*Cox and Snell*	*0.01*		*0.02*		*0.03*		*0.08*	
*Nagelkerke*	*0.03*		*0.10*		*0.11*		*0.18*	

	Somatization		IBS		Fibromyalgia		IHD	
	OR	95 % CI	OR	95 % CI	OR	95 % CI	OR	95 % CI
Cluster membership^a^								
Cluster 1	1.22	0.76–1.95	2.05∗∗	1.26–3.34	0.93	0.37–2.36	0.90	0.68–1.20
Cluster 3	4.27∗∗∗	2.47–7.38	3.52∗∗	1.83–6.78	0.60	0.08–4.72	1.20	0.74–1.97
Cluster 4	6.55∗∗∗	3.71–11.57	2.60∗∗∗	1.56–7.08	2.94	0.79–10.93	1.14	0.61–2.11
*Pseudo R-square*								
*Cox and Snell*	*0.02*		*0.01*		*0.00*		*0.08*	
*Nagelkerke*	*0.08*		*0.05*		*0.05*		*0.19*	

	COPD		Type 2 Diabetes		Cancer		Obesity	
	OR	95 % CI	OR	95 % CI	OR	95 % CI	OR	95 % CI
Cluster membership^a^								
Cluster 1	1.32	0.73–2.41	0.88	0.62–1.25	1.27	0.89–1.82	0.93	0.74–1.14
Cluster 3	1.57	0.59–4.21	1.45	0.83–2.52	1.94∗	1.12–3.36	1.31	0.94–1.84
Cluster 4	2.99∗	1.17–7.62	1.25	0.60–2.58	0.63	0.22–1.76	1.04	0.68–1.58
*Pseudo R-square*								
*Cox and Snell*	*0.01*		*0.05*		*0.02*		*0.02*	
*Nagelkerke*	*0.08*		*0.16*		*0.08*		*0.04*	

	Heavy smoking		Alcohol misuse misuse		Drug abuse	
	OR	95 % CI	OR	95 % CI	OR	95 % CI
Cluster membership^a^						
Cluster 1	1.53∗	1.10–2.13	1.95∗∗∗	1.60–2.37	1.10	0.36–3.32
Cluster 3	2.75∗∗∗	1.72–4.38	2.85 ∗∗∗	2.11–3.85	0.91	0.11–7.67
Cluster 4	1.88∗	0.82–2.48	3.98∗∗∗	2.86–5.53	9.95∗∗∗	3.21–30.87
*Pseudo R-square*						
*Cox and Snell*	*0.03*		*0.07*		*0.01*	
*Nagelkerke*	*0.09*		*0.11*		*0.08*	

∗∗∗ = p < 0.001. ∗∗ = p < 0.01. ∗ = 0.05, all models controlled for age, self-reported parental immigrant status and parents' highest level of education.

a Cluster 2 used as reference.

**Fig. 2:**
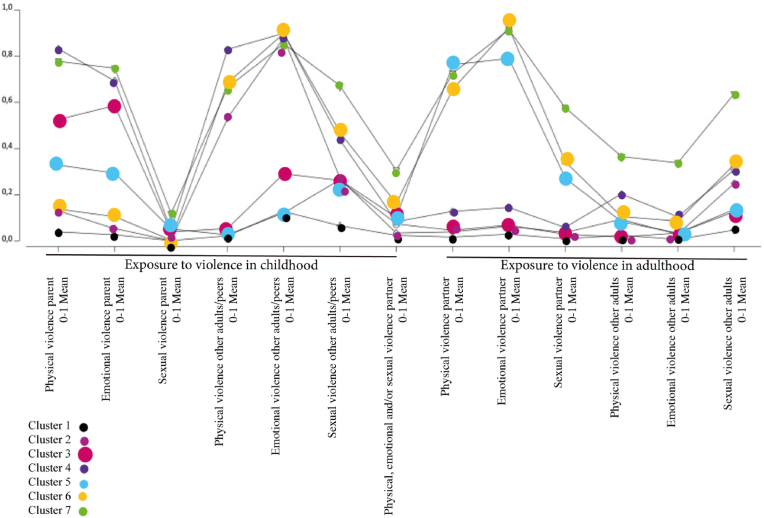
Women, plot of mean likelihood for exposure to violence for seven classes (mean value for respective cluster).

Among female respondents (Table 5), the analysis indicated that a seven-cluster model gave the best fit using the applied criteria. Similar to the case for men, the AIC was somewhat lower for a more complex model (eight clusters), but BIC was lowest for a seven-cluster model. Given the sample size and acceptable entropy, the seven-cluster model was thus chosen. Fig. 2 illustrates the seven cluster model regarding exposure to violence in a life-course perspective for women plotted against mean values for the violence variables. Table 6 demonstrates the likelihood of violence exposure among women according to cluster with respect to perpetrator type in childhood and adulthood.

**Table 5:** Women, indicators of fit for models with one through eight latent classes.

Classes	LL	BIC(LL)	AIC(LL)	L^2^	df	p-value	Entropy R^2^
1	−26871,1	53853,1	53768,1	9169,3	5094	1,8e-237	1,00
2	−24665,5	49561,5	49385,0	4758,1	5080	1,00	0,69
3	−24119,3	48588,6	48320,5	3665,7	5066	1,00	0,71
4	−23888,9	48247,4	47887,8	3205,0	5052	1,00	0,72
5	−23692,6	47974,3	47523,1	2812,3	5038	1,00	0,71
6	−23606,9	47922,5	47379,9	2641,0	5024	1,00	0,70
7^a^	−23524,5	47877,3	47243,0	2476,2	5010	1,00	0,67
8	−23473,6	47895,0	47169,2	2374,4	4996	1,00	0,67

a Best fitting model according to criterion.

**Table 6:**
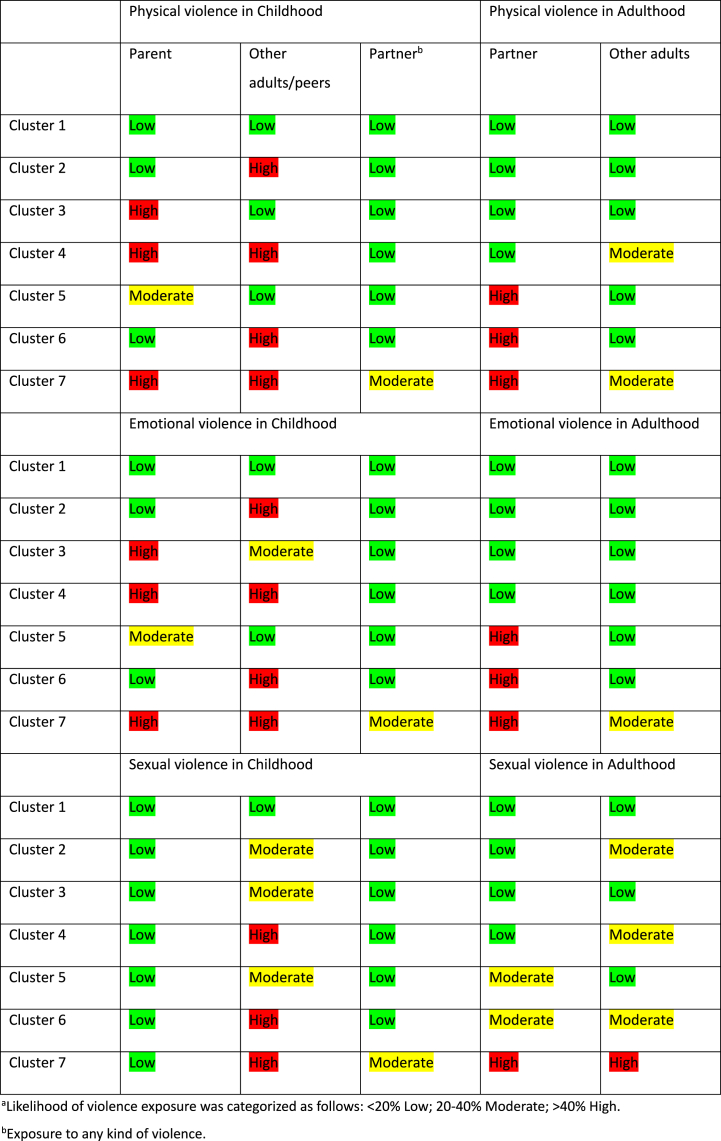
Likelihood^a^ of violence exposure among women according to cluster with respect to perpetrator type in childhood and adulthood.

Cluster 1 represents 40.8 % of the female sample. This group showed a low likelihood of exposure to all types of violence in both childhood and adulthood and was therefore used as the reference group in the logistic regression.

Cluster 2 included 23.3 % of the sample. In this group, physical and emotional violence by other adults/peers was highly likely in childhood. Exposure to sexual violence by other adults/peers was moderately likely before the age of 18. In adulthood, exposure to sexual violence by other adults was moderately likely.

Cluster 3 comprised 11.9 % of the sample. This group was characterized by a high likelihood of exposure to physical and emotional violence from a parent and moderate likelihood of emotional and sexual violence by other adults/peers in childhood. The likelihood of all types of violence in adulthood was low.

Cluster 4 included 8.1 % of the sample. This group had high likelihood of exposure to physical and emotional violence by a parent and a high likelihood of exposure to all types of violence by other adults/peers in childhood. In adulthood, exposure to physical and sexual violence by other adults was moderately likely.

Cluster 5 represented 5.8 % of the female sample. In this group, physical and emotional violence by a parent, as well as exposure to sexual violence by other adults/peers, was moderately likely in childhood. In adulthood, exposure to physical and emotional violence by a partner was highly likely. Exposure to sexual violence was moderately likely by both partner and other adults.

Cluster 6 included 5.7 % of the sample. This group was characterized by a high likelihood of physical, emotional and sexual violence by other adults/peers in childhood. The group showed a high likelihood of exposure to physical and emotional violence as well as a moderate likelihood of sexual violence by a partner in adulthood. Exposure to sexual violence by other adults after the age of 18 was moderately likely.

Cluster 7 comprised 4.5 % of the sample. This group showed the highest multiplicity of violence exposure i.e. high likelihood of physical and emotional violence by parent and high likelihood of physical, emotional and sexual violence by other adults/peers during childhood. In addition, there was a moderate likelihood of any violence exposure by a intimate partner before the age of 18. In adult life, the group showed a high likelihood of violence by a partner, moderate likelihood of physical and emotional violence and high likelihood of sexual violence by other adults.

Table 7 shows that cluster membership among women was significantly associated with all examined mental health problems, as well as with somatization and IBS, with the exceptions of depression in cluster 3 and IBS in cluster 2. In addition, significant positive associations were found for some clusters with respect to fibromyalgia, IHD, COPD, cancer and obesity. Regarding health-related risk behaviors, alcohol misuse was significantly associated with all clusters. Heavy smoking was linked to clusters 4, 5, and 7, while drug abuse was only significantly associated with clusters 5 and 7.

**Table 7:** Cluster membership and associations with health problems and health-related risk behaviors among women, logistic regression models (odds ratios with 95 % confidence intervals).

	Depression		Anxiety		PTSS		Self-harm	
	OR	95 % CI	OR	95 % CI	OR	95 % CI	OR	95 % CI
Cluster membership^a^								
Cluster 2	1.87∗∗∗	1.35–2.58	1,92∗∗∗	1.31–2.81	2.61∗∗∗	1.82–3.74	2.78∗∗∗	2.08–3.53
Cluster 3	1.46	0.94–2.27	1.73∗	1.05–2.87	3.75∗∗∗	2.49–5.63	2.35∗∗∗	1.63–3.33
Cluster 4	5,15∗∗∗	3.63–7.30	4.94∗∗∗	3.25–7.50	6.02∗∗∗	4.03–9.00	7.73∗∗∗	5.48–10.21
Cluster 5	2.73∗∗∗	1.72–4.34	2.53∗∗	1.40–4.58	4.65∗∗∗	2.85–7.59	4.55∗∗∗	3.00–6.56
Cluster 6	5.40∗∗∗	3.66–7.95	7.30∗∗∗	4,73–11.26	10.72∗∗∗	7.11–16.16	8.41∗∗∗	5.70–11.26
Cluster 7	5.59∗∗∗	3.59–8.70	9.78∗∗∗	6.17–15.49	24.49∗∗∗	16.16–37.09	30.21∗∗∗	20.0–42.48
*Pseudo R-square*								
*Cox and Snell*	*0.04*		*0.05*		*0.08*		*0.14*	
*Nagelkerke*	*0.09*		*0.13*		*0.18*		*0.25*	

	Somatization		IBS		Fibromyalgia		IHD	
	OR	95 % CI	OR	95 % CI	OR	95 % CI	OR	95 % CI
Cluster membership^a^								
Cluster 2	2.04∗∗∗	1.44–2.90	1.34	0.94–1.90	1.54	0.94–2.51	1.06	0.62–1.80
Cluster 3	2.13∗∗∗	1.40–3.22	1.60∗	1.06–2.41	1.05	0.55–1.98	0.73	0.37–1.45
Cluster 4	3.58∗∗∗	2.37–5.40	2.07∗∗∗	1.34–3.20	1.71	0.88–3.35	2.54∗∗	1.43–4.48
Cluster 5	2.94∗∗∗	1.82–4.75	2.00∗∗	1.23–3.26	2.22∗∗	1.18–4.17	0.49	0.15–1.58
Cluster 6	5.27∗∗∗	3.40–8.16	2.54∗∗∗	1.59–4.06	4.63∗∗∗	2.60–8.26	2.69∗∗	1.35–5.33
Cluster 7	8.64∗∗∗	5.60–13.34	3.41∗∗∗	2.09–5.56	3.54∗∗∗	1.78–7.07	2.07	0.91–4.71
*Pseudo R-square*								
*Cox and Snell*	*0.04*		*0.01*		*0.03*		*0.03*	
*Nagelkerke*	*0.09*		*0.03*		*0.12*		*0.13*	

	COPD		Type 2 Diabetes		Cancer		Obesity	
	OR	95 % CI	OR	95 % CI	OR	95 % CI	OR	95 % CI
Cluster membership^a^								
Cluster 2	1.18	0.57–2.45	0.61	0.33–1.15	1.31	0.89–1.93	0.86	0.67–1.11
Cluster 3	1.22	0.55–2.68	0.49	0.22–1.08	1.03	0.64–1.66	0.83	0.60–1.15
Cluster 4	0.28	0.04–2.10	0.91	0.41–2.03	1.58	0.94–2.66	1.40∗	1.01–1.94
Cluster 5	3.11∗∗	1.43–6.76	1.37	0.67–2.84	0.90	0.46–1.76	0.84	0.55–1.28
Cluster 6	4.16∗∗∗	1.82–9.50	0.60	0.18–1.94	1.93∗	1.08–3.44	1.30	0.89–1.91
Cluster 7	4.17∗∗	1.74–9.99	0.75	0.23–2.45	1.59	0.80–3.16	1.50	0.98–2.32
*Pseudo R-square*								
*Cox and Snell*	*0.02*		*0.03*		*0.03*		*0.02*	
*Nagelkerke*	*0.15*		*0.13*		*0.11*		*0.03*	

	Heavy smoking		Alcohol misuse		Drug abuse	
	OR	95 % CI	OR	95 % CI	OR	95 % CI
Cluster membership^a^						
Cluster 2	1.00	0.56–1.80	1.63∗∗∗	1.31–2.02	1.14	0.19–6.88
Cluster 3	1.49	0.81–2.75	1.48∗∗	1.10–1.98	0.00	0.00-.
Cluster 4	3.16∗∗∗	1.80–5.57	2.25∗∗∗	1.66–3.05	1.79	0.19–17.39
Cluster 5	2.49∗∗	1.31–4.75	1.78∗∗	1.23–2.58	9.45∗∗	1.87–47.84
Cluster 6	2.10	1.00–4.43	2.36∗∗∗	1.68–3.32	2.50	0.26–24.34
Cluster 7	3.94∗∗∗	2.00–7.75	4.35∗∗∗	3.04–6.23	15.56∗∗∗	3.42–70.69
*Pseudo R-square*						
*Cox and Snell*	*0.02*		*0.08*		*0.01*	
*Nagelkerke*	*0.07*		*0.14*		*0.14*	

∗∗∗ = p < 0.001. ∗∗ = p < 0.01. ∗ = 0.05, all models controlled for age, self-reported parental immigrant status and parents' highest level of education.

a Cluster 1 used as reference.

## 5 Discussion

This study empirically analyzed the patterns of exposure to physical, emotional and sexual violence in a lifetime perspective, and additionally investigated gender disparities and if group membership was correlated with physical and mental health problems and health-related risk behaviors in adulthood. This was done using Latent Class Analysis (LCA) on data from a nationally representative sample of 10 337 men and women aged 18–74 in Sweden. To our knowledge, this is the first study to use this methodology to investigate these associations. The following section discusses the results within the framework of trauma theory, as conceptualized through TTB.

### 5.1 Patterns of exposure to poly-victimization

Previous research using person-centered statistical techniques regarding intimate partner violence suggest that patterns of exposure to different types of violence are more complex among women compared to men (Ansara & Hindin, 2010). The present analyses confirm this by suggesting a best fit of seven clusters for women and a best fit of four clusters for men.

Overall, distinct patterns of exposure to violence over the life-course were revealed among both women and men. A large proportion, nearly half, of both women and men share a pattern of low likelihood of exposure to violence in a life-course perspective. All groups of the poly-victimized men share a history of exposure to physical and emotional violence by peers and other adults in childhood and physical violence by other adults in adulthood. The results agree with previous research in Sweden, which shows that poly-victimized boys are mainly exposed to violence by peers (Källström et al., 2020). Clusters 3 and 4 also showed a high likelihood of exposure to physical and emotional violence by parents and cluster 4 also had a high likelihood of physical and emotional partner violence in adulthood. According to TTB, individuals exposed to violence who have or are able to develop resilience factors including safety, autonomy, trauma awareness and trust are more likely to have positive coping strategies and reduced risk of remaining in trauma-perpetuating environments (Marks et al., 2022). Individuals in cluster 1 may constitute a comparatively more resilient subgroup relative to those in clusters 3 and 4, who exhibit a higher likelihood of experiencing violence in adulthood.

Among women, clusters 2 showed patterns of poly-victimization in a life-course perspective that were similar to cluster 1 among men. However, the analyses showed three unique patterns found only among women. In cluster 5, members had moderate to high likelihood of adult partner violence with a history of only moderate likelihood of exposure to violence in childhood. Cluster 7 was the only group within either gender with a moderate likelihood of exposure to any type of violence by a partner in childhood. Cluster 3 constitutes possibly one of the more prominent patterns at the group level in this analysis. The members were characterized by moderate to high likelihood of exposure to poly-victimization in childhood but only low likelihood of revictimization in adult life. These findings demonstrate that a substantial proportion (11.9 %) of women who experienced poly-victimization in childhood deviate from a conventional trajectory of violence exposure across the life course. This complements previous research regarding lifetime revictimization (McIntyre & Widom, 2011; Widom et al., 2008) by highlighting the existence of alternative, more positive pathways that may reduce the risk of revictimization in adulthood. Adopting a resilience-based approach, this group merits closer examination. They may represent a subgroup that has developed more advanced resilience capacities than several other clusters with comparable histories of childhood violence exposure, which appear to be at greater risk of continued victimization in adulthood. It is plausible that individuals in this group have cultivated protective strategies associated with resilience, including a heightened sense of safety, autonomy, trauma awareness, and trust - factors that are commonly linked to more adaptive coping mechanisms according to TTB (Marks et al., 2022). From a trauma-to-benefit perspective, early intervention is crucial to disrupt the progression of violence exposure and reduce the risk of revictimization. This group may therefore represents a strategically important focus for future research on resilience and protective factors.

### 5.2 Gendered patterns of exposure to sexual violence and intimate partner violence

The separate analyses for women and men showed similarities in patterns of poly-victimization, but also distinct gender disparities. The most striking difference concerns the likelihood of exposure to sexual violence and intimate partner violence. Among men, all patterns showed only low likelihood of exposure to any type of violence by a partner in childhood and exposure to sexual violence and adult partner violence are only found in cluster 4. In clusters 1 and 3, men showed only low likelihood of exposure to sexual violence in childhood as compared to moderate to high likelihood in clusters 2–7 among women, which is in line with previous data from Sweden (Jernbro & Landberg, 2020).

Among women, all poly-victimized groups had a moderate to high likelihood of exposure to sexual violence in childhood by peers and other adults as well as in adulthood by partners or other adults with the exception of members of cluster 3. Members of cluster 5, cluster 6 and cluster 7 showed moderate to high likelihood of exposure to violence by a partner in adulthood. Notably, these groups differ in their patterns of likelihood of violence exposure, type of violence and perpetrators before the age of 18, prior to victimization by an adult partner. Most prominent is the pattern of cluster 5, which shows that moderate to high likelihood of adult partner violence is not necessarily preceded by a high likelihood of violence exposure in childhood. This adds nuances to existing knowledge on poly-victimization (Finkelhor et al., 2009; Turner et al., 2010) and revictimization (McIntyre & Widom, 2011; Widom et al., 2008). From a trauma-to-benefit perspective, experiencing a single form of violence may not necessarily increase the risk of subsequent victimization, possibly due to the presence of resilience factors according to TTB. Moreover, not all adults exposed to partner violence have a history of childhood abuse. Previous research has indicated that other circumstances could elevate the risk of intimate partner violence in adulthood, independent of early victimization. In a systematic review Capaldi et al. (2012) for instance found that socioeconomic stressors such as unemployment and financial strain, psychological vulnerabilities including depression and poor conflict resolution skills, substance abuse, and social isolation were significant predictors of partner violence. The pattern of cluster 5 underscores the importance of considering multiple pathways to adult victimization beyond childhood trauma, thereby emphasizing the need for further investigation in future research.

### 5.3 Group membership and associations with health problems and health-related risk behaviors

Overall, the analysis showed that female and male members in groups with a high likelihood of victimization from multiple perpetrators over the lifespan exhibited more mental and physical health problems and health-related risk behaviors compared to members in groups with low and moderate likelihood of victimization. Previous research in Sweden has shown associations between the “dose” of poly-victimization and the risk of health problems and health-related risk behaviors in childhood (Annerbäck et al., 2012; Jernbro & Landberg, 2020) and adulthood (Källström et al., 2020; Pettersson et al., 2025; Simmons et al., 2015; Simmons & Swahnberg, 2021). Analyses in the US and the UK have also shown strong associations between the “dose” of adversities such as exposure to different types of violence in childhood and mental and physical health problems as well as health-related risk behaviors in adulthood (Madigan et al., 2023). Overall, women showed significant associations with a larger number of health problems and a tendency towards higher odds ratios as compared to men, especially in cluster 7.

In general, the results showed stronger associations between polyvictimization and mental health problems compared to physical health problems, which is in line with our previous findings regarding life-course exposure to violence (Pettersson et al., 2025). These findings also corroborate those of previous cross-sectional studies, mostly from the UK and US, where higher odds ratios were shown for mental health issues compared to physical disease outcomes among adults with a history of polyvictimization during childhood (Bellis et al., 2014; Petruccelli et al., 2019). Among women (clusters 4, 5, 6, and 7) and men (clusters 3 and 4) exposed to violence in both childhood and adulthood, the risk was particularly high for mental health problems, most markedly PTSS (OR 4.4–24.5) and self harm (OR 7.7–30.2). The more polyvictimized clusters were also the only ones that showed significant associations to IHD (women only, OR 2.5–2.7) and COPD (OR 3.0–4.2) suggesting that the perpetuation of violence exposure over the lifespan may more likely predispose to physical disease compared to shorter term exposure. The most polyvictimized clusters (women cluster 7, men cluster 4) also showed the highest odds of alcohol misuse (OR 4.0–4.4) and drug abuse (OR 10.0–15.6). Substance abuse per se may represent both a consequence of and a cause of violence exposure. To our knowledge, no previous studies have examined health outcomes in relation to polyvictimization in both childhood and adulthood using LCA, making comparisons with the present findings difficult. However, a systematic review and meta-analysis of 37 studies that gave risk estimates for 23 health outcomes comparing individuals exposed to four or more adverse childhood experiences to unexposed individuals showed ORs of 2–3 for poor self-rated health, cancer, heart disease, and respiratory disease, 3–6 for mental ill health and heavy alcohol use and more than 7 for self harm and problematic drug use (Huges et al., 2017).

The analyses of associations between group-membership and mental and physical health problems and health-related risk behaviors also suggest that the health correlates of poly-victimization extend beyond the mere “dose” of victimization in childhood shown in many ACE-studies (Felitti et al., 1998; Madigan et al., 2023). For example, among men, all the victimized groups showed exposure to multiple violence types by other adults/peers in childhood and physical violence by other adults in adulthood, but when parental violence (cluster 3) or both parental and adult partner violence (cluster 4) were also present, the odds of mental health problems were significantly higher than for the poly-victimized group without these experiences (cluster 1). Among women, the groups showing multiple exposure to violence in both childhood and adulthood (clusters 4, 5, 6 and 7) showed higher odds ratios for mental health problems and were the only groups showing significant associations to physical health problems and some health-related risk behaviors compared to the groups with multiple childhood violence exposure without poly-victimization in adulthood (clusters 2 and 3). In addition, women share the same experiences of poly-victimization by an adult partner as men (cluster 4) in groups 5, 6 and 7. According to TTB theory, exposure to parental violence in childhood and partner violence in adulthood may serve as both moderators and mediators of severe mental health problems, adverse physical health outcomes, and increased engagement in health-risk behaviors. Over the past two decades, research in stress physiology, neurobiology, and epigenetics (Jiang et al., 2019) has illuminated the mechanisms central to TTB theory—trauma exposure, physiological response, and resilience. Childhood maltreatment has been shown to activate stress systems, leading to long-term neurodevelopmental disruptions known as toxic stress (Bucci et al., 2016), particularly in brain regions governing cognition and emotional regulation, which are associated with poorer mental health outcomes (Stargel et al., 2022). Toxic stress has also been implicated in poor physical health outcomes following exposure to abuse and neglect during childhood, including IHD, COPD and cancer, through physiological and epigenetic effects on gene regulation and homeostatsis (Parade et al., 2021). Lifestyle behaviors, including smoking and alcohol consumption, may also compound these effects in a biopsychosocial context revolving on maladaptive coping mechanisms (Huges et al., 2017). These findings highlight the importance of a life-course perspective on violence exposure, as adult revictimization—especially intimate partner violence—may amplify the effects of earlier trauma and result in even more severe biopsychosocial consequences. While childhood adversity remains a critical factor, emerging research (Pettersson et al., 2025) suggests that adult trauma, which is often underrepresented in ACE studies, may also play a significant role in shaping long-term health trajectories.

### 5.4 Implications for future research

The findings of the present study support previous research on poly-victimization and revictimization in relation to violence exposure, while also offering new insights into the complexity of these phenomena. The distinct patterns of victimization across childhood and adulthood, the differential health risks associated with these experiences, and the observed gender differences, including the unique trajectories of certain clusters among women, suggest that disparities may exist in individuals’ ability to adopt preventive health actions and to decrease the risk of being exposed to harmful environments assoiated to revictimization later in life. These differences can be interpreted through the trauma-to-benefit (TTB) framework, but are also likely shaped by individual, familial, social, and societal factors (Scott-Storey, 2011). Overall, the results of this study highlight the need for further investigation to deepen our understanding of the interplay between violence exposure and health outcomes across the life course.

## 6 Strengths and limitations

A strength of this study is that it is based on a large and nationally representative sample of both women and men. Furthermore, the risk of non-response bias has been decreased by careful non-response analysis and subsequent calibration and weighting of data, based on sets of information from national registries.

Limitations of this study include the retrospective nature of the data, which may entail recall bias. As in all survey studies, the present results are limited by the cross-sectional nature of the data, with fixed responses to complicated issues. Another limitation is the relatively broad measures of victimization used to assess violence, without detailed information regarding the circumstances, frequency or duration of each type of violence exposure. For example, we cannot discern to what extent the results apply to specific subgroups, such as children repeatedly victimized or adults occasionally victimized decades ago. It should also be noted that although the cutoff of 20 % for a positive indicator of moderate likelihood of exposure to violence may appear relatively high, a lower cutoff would not have changed the patterns of exposure to violence. The survey was carried out in 2012, and patterns of violence exposure and health problems may have changed somewhat during the years that have passed since the data were collected. A further limitation is that gender-specific LCAs were estimated separately, meaning that class structures cannot be directly compared between women and men. The models are optimized within each gender and may capture partly different underlying dimensions. Thus, observed differences in the number or nature of classes should be interpreted with caution. Future research could apply multi-group LCA techniques (e.g., Collins & Lanza, 2010; Masyn, 2013) to formally test measurement invariance and allow for direct comparison of class structures across gender. Another limitation is that there is no possibility to determine whether the health problems and health-related risk behaviors were present before or after the initial event of victimization.

## CRediT authorship contribution statement

**Rickard Pettersson:** Writing – review & editing, Writing – original draft, Methodology, Formal analysis, Data curation, Conceptualization. **Steven Lucas:** Writing – review & editing, Data curation, Conceptualization. **Mattias Strandh:** Writing – review & editing, Methodology, Conceptualization.

## Ethical statement

Research has been conducted in accordance to the Declaration of Helsinki, and the study was approved by the Ethical Review Board in Uppsala (Dnr 2011/156). Questionnaires were distributed to 10 000 women and 10 000 men 18–74 years of age randomly selected from the population registry of Sweden. An introduction letter was sent informing about the study and that the individual could choose not to participate by contacting the research group or Statistics Sweden. Respondents were informed that by completing the online or paper version of the questionnaire they gave their informed consent to participate.

## Funding

This work was supported by the Crime Victim Fund (grant no. 04647/2014).

## Declaration of competing interest

The authors declare that they have no competing financial interests or personal relationships that could have appeared to influence the work of this manuscript.

## Data Availability

The data that has been used is confidential.
