# Supplementary material for: Patterns of violence exposure in a life-course perspective and associations to mental and physical health problems and health-related risk behaviors among women and men in Sweden: A latent class analysis
Source: SSM Popul Health. 2025 Oct 23;32:101874. doi: 10.1016/j.ssmph.2025.101874 (PMC12637257; doi:10.1016/j.ssmph.2025.101874)
Supplement: Multimedia component 1 [file mmc1.pdf]

Logistic Regression

Notes

Output Created		26-AUG-2025 11:44:23
Comments		
Input	Data	/Users/stevenlc/Library/CloudStorage/OneDrive-Privat/ICloud filer/Doktorander/Rickard/Artikel 4/Menfour class model.sav
	Active Dataset	DataSet3
	File Label	Scored Data File
	Filter	<none>
	Weight	<none>
	Split File	<none>
	N of Rows in Working Data File	4656
Missing Value Handling	Definition of Missing	User-defined missing values are treated as missing
Syntax		LOGISTIC REGRESSION VARIABLES HAD_probable_depression_ny /METHOD=ENTER Cluster_model_men /CONTRAST (Cluster_model_men)=INDICATOR(1) /PRINT=CI(95) /CRITERIA=PIN(0.05) POUT(0.10) ITERATE(20) CUT(0.5).
Resources	Processor Time	00:00:00,20
	Elapsed Time	00:00:00,00

Case Processing Summary

Unweighted Cases[a]		N	Percent
Selected Cases	Included in Analysis	4087	87.8
	Missing Cases	569	12.2

	Total	4656	100.0
Unselected Cases		0	.0
Total		4656	100.0

a. If weight is in effect, see classification table for the total number of cases.

Dependent Variable Encoding

Original Value	Internal Value
Nej	0
Ja	1

Categorical Variables Codings

			Parameter coding		
		Frequency	(1)	(2)	(3)
Cluster_model_men	1.00	1751	.000	.000	.000
	2.00	1760	1.000	.000	.000
	3.00	335	.000	1.000	.000
	4.00	241	.000	.000	1.000

Block 0: Beginning Block

Classification Table[a,b]

			Predicted		
			Depression_ny		Percentage Correct
	Observed		Nej	Ja	
Step 0	Depression_ny	Nej	3760	0	100.0
		Ja	327	0	.0
	Overall Percentage				92.0

a. Constant is included in the model.

b. The cut value is ,500

Variables in the Equation

		B	S.E.	Wald	df	Sig.	Exp(B)
Step 0	Constant	-2.442	.058	1794.314	1	.000	.087

Variables not in the Equation

			Score	df	Sig.
Step 0	Variables	Cluster_model_men	39.477	3	<.001
		Cluster_model_men(1)	.045	1	.832
		Cluster_model_men(2)	19.848	1	<.001
		Cluster_model_men(3)	12.976	1	<.001
	Overall Statistics		39.477	3	<.001

Block 1: Method = Enter

Omnibus Tests of Model Coefficients

		Chi-square	df	Sig.
Step 1	Step	34.978	3	<.001
	Block	34.978	3	<.001
	Model	34.978	3	<.001

Model Summary

Step	-2 Log likelihood	Cox & Snell R Square	Nagelkerke R Square
1	2243.878[a]	.009	.020

a. Estimation terminated at iteration number 5 because parameter estimates changed by less than ,001.

Classification Table[a]

	Observed		Predicted		
			Depression_ny		Percentage Correct
			Nej	Ja	
Step 1	Depression_ny	Nej	3760	0	100.0
		Ja	327	0	.0
	Overall Percentage				92.0

a. The cut value is ,500

Variables in the Equation

		B	S.E.	Wald	df	Sig.	Exp(B)	95% C.I.for EXP(B)	
								Lower	Upper
Step 1[a]	Cluster_model_men			37.732	3	<.001			
	Cluster_model_men(1)	.286	.134	4.573	1	.032	1.331	1.024	1.729
	Cluster_model_men(2)	.954	.185	26.476	1	<.001	2.595	1.805	3.733

	Cluster_model_men(3)	.936	.210	19.771	1	<.001	2.549	1.687	3.850
	Constant	-2.742	.100	748.753	1	<.001	.064		

a. Variable(s) entered on step 1: Cluster_model_men.

Logistic Regression

Notes

Output Created		26-AUG-2025 11:44:23
Comments		
Input	Data	/Users/stevenlc/Library/CloudStorage/OneDrive-Privat/ICloud filer/Doktorander/Rickard/Artikel 4/Menfour class model.sav
	Active Dataset	DataSet3
	File Label	Scored Data File
	Filter	<none>
	Weight	<none>
	Split File	<none>
	N of Rows in Working Data File	4656
Missing Value Handling	Definition of Missing	User-defined missing values are treated as missing
Syntax		LOGISTIC REGRESSION VARIABLES HAD_probable_ångest_ny<br>/METHOD=ENTER Cluster_model_men<br>/CONTRAST (Cluster_model_men)=INDICATOR(1)<br>/PRINT=CI(95)<br>/CRITERIA=PIN(0.05) POUT(0.10) ITERATE(20) CUT(0.5).
Resources	Processor Time	00:00:00,19
	Elapsed Time	00:00:00,00

Case Processing Summary

Unweighted Cases[a]		N	Percent
Selected Cases	Included in Analysis	4077	87.6
	Missing Cases	579	12.4
	Total	4656	100.0
Unselected Cases		0	.0
Total		4656	100.0

a. If weight is in effect, see classification table for the total number of cases.

Dependent Variable Encoding

Original Value	Internal Value
Nej	0
Ja	1

Categorical Variables Codings

		Frequency	Parameter coding (1)	(2)	(3)
Cluster_model_men	1.00	1747	.000	.000	.000
	2.00	1760	1.000	.000	.000
	3.00	331	.000	1.000	.000
	4.00	239	.000	.000	1.000

Block 0: Beginning Block

Classification Table[a,b]

Observed			Predicted Anxiety_ny Nej	Ja	Percentage Correct
Step 0	Anxiety_ny	Nej	3955	0	100.0
		Ja	122	0	.0
	Overall Percentage				97.0

a. Constant is included in the model.

b. The cut value is ,500

Variables in the Equation

		B	S.E.	Wald	df	Sig.	Exp(B)
Step 0	Constant	-3.479	.092	1432.199	1	.000	.031

Variables not in the Equation

			Score	df	Sig.
Step 0	Variables	Cluster_model_men	84.974	3	<.001
		Cluster_model_men(1)	2.587	1	.108
		Cluster_model_men(2)	22.504	1	<.001
		Cluster_model_men(3)	54.394	1	<.001
	Overall Statistics		84.974	3	<.001

Block 1: Method = Enter

Omnibus Tests of Model Coefficients

		Chi-square	df	Sig.
Step 1	Step	61.408	3	<.001
	Block	61.408	3	<.001
	Model	61.408	3	<.001

Model Summary

Step	-2 Log likelihood	Cox & Snell R Square	Nagelkerke R Square
1	1035.124[a]	.015	.063

a. Estimation terminated at iteration number 7 because parameter estimates changed by less than ,001.

Classification Table[a]

			Predicted		
			Anxiety_ny		Percentage Correct
Observed			Nej	Ja	
Step 1	Anxiety_ny	Nej	3955	0	100.0
		Ja	122	0	.0
	Overall Percentage				97.0

a. The cut value is ,500

Variables in the Equation

B	S.E.	Wald	df	Sig.	Exp(B)	95% C.I.for EXP(B) Lower	Upper

Step 1[a]	Cluster_model_men			69.478	3	<.001			
	Cluster_model_men(1)	.454	.244	3.454	1	.063	1.574	.976	2.540
	Cluster_model_men(2)	1.568	.285	30.290	1	<.001	4.799	2.745	8.390
	Cluster_model_men(3)	2.014	.282	51.057	1	<.001	7.494	4.313	13.021
	Constant	-4.117	.191	467.051	1	<.001	.016		

a. Variable(s) entered on step 1: Cluster_model_men.

Logistic Regression

Notes

Output Created		26-AUG-2025 11:44:23
Comments		
Input	Data	/Users/stevenlc/Library/CloudStorage/OneDrive-Privat/ICloud filer/Doktorander/Rickard/Artikel 4/Menfour class model.sav
	Active Dataset	DataSet3
	File Label	Scored Data File
	Filter	<none>
	Weight	<none>
	Split File	<none>
	N of Rows in Working Data File	4656
Missing Value Handling	Definition of Missing	User-defined missing values are treated as missing
Syntax		LOGISTIC REGRESSION VARIABLES PTSD_score_pos_ny /METHOD=ENTER Cluster_model_men /CONTRAST (Cluster_model_men)=INDICATOR(1) /PRINT=CI(95) /CRITERIA=PIN(0.05) POUT(0.10) ITERATE(20) CUT(0.5).
Resources	Processor Time	00:00:00,19
	Elapsed Time	00:00:01,00

Case Processing Summary

Unweighted Cases[a]		N	Percent
Selected Cases	Included in Analysis	4024	86.4
	Missing Cases	632	13.6
	Total	4656	100.0
Unselected Cases		0	.0
Total		4656	100.0

a. If weight is in effect, see classification table for the total number of cases.

Dependent Variable Encoding

Original Value	Internal Value
Nej	0
Ja	1

Categorical Variables Codings

			Parameter coding		
		Frequency	(1)	(2)	(3)
Cluster_model_men	1.00	1738	.000	.000	.000
	2.00	1731	1.000	.000	.000
	3.00	322	.000	1.000	.000
	4.00	233	.000	.000	1.000

Block 0: Beginning Block

Classification Table[a,b]

			Predicted		
			PTSS_ny		Percentage
	Observed		Nej	Ja	Correct
Step 0	PTSS_ny	Nej	3829	0	100.0
		Ja	195	0	.0
	Overall Percentage				95.2

a. Constant is included in the model.

b. The cut value is ,500

Variables in the Equation

		B	S.E.	Wald	df	Sig.	Exp(B)
Step 0	Constant	-2.977	.073	1644.843	1	.000	.051

Variables not in the Equation

			Score	df	Sig.
Step 0	Variables	Cluster_model_men	154.829	3	<.001
		Cluster_model_men(1)	2.604	1	.107
		Cluster_model_men(2)	27.542	1	<.001
		Cluster_model_men(3)	112.263	1	<.001
	Overall Statistics		154.829	3	<.001

Block 1: Method = Enter

Omnibus Tests of Model Coefficients

		Chi-square	df	Sig.
Step 1	Step	110.054	3	<.001
	Block	110.054	3	<.001
	Model	110.054	3	<.001

Model Summary

Step	-2 Log likelihood	Cox & Snell R Square	Nagelkerke R Square
1	1450.882[a]	.027	.084

a. Estimation terminated at iteration number 6 because parameter estimates changed by less than ,001.

Classification Table[a]

	Observed		Predicted: PTSS_ny Nej	Predicted: PTSS_ny Ja	Predicted: Percentage Correct
Step 1	PTSS_ny	Nej	3829	0	100.0
		Ja	195	0	.0
	Overall Percentage				95.2

a. The cut value is ,500

Variables in the Equation

		B	S.E.	Wald	df	Sig.	Exp(B)	95% C.I.for EXP(B) Lower	Upper
Step 1[a]	Cluster_model_men			122.828	3	<.001			
	Cluster_model_men(1)	.575	.197	8.556	1	.003	1.778	1.209	2.614
	Cluster_model_men(2)	1.594	.238	45.019	1	<.001	4.925	3.091	7.845
	Cluster_model_men(3)	2.269	.228	99.084	1	<.001	9.666	6.184	15.109
	Constant	-3.698	.156	560.587	1	<.001	.025		

a. Variable(s) entered on step 1: Cluster_model_men.

Logistic Regression

Notes

Output Created		26-AUG-2025 11:44:24
Comments		
Input	Data	/Users/stevenlc/Library/CloudStorage/OneDrive-Privat/ICloud filer/Doktorander/Rickard/Artikel 4/Menfour class model.sav
	Active Dataset	DataSet3
	File Label	Scored Data File
	Filter	<none>
	Weight	<none>
	Split File	<none>
	N of Rows in Working Data File	4656
Missing Value Handling	Definition of Missing	User-defined missing values are treated as missing

Syntax		LOGISTIC REGRESSION VARIABLES Any_selfharm_ny<br>/METHOD=ENTER Cluster_model_men<br>/CONTRAST (Cluster_model_men)=INDICATOR(1)<br>/PRINT=CI(95)<br>/CRITERIA=PIN(0.05) POUT(0.10) ITERATE(20) CUT(0.5).
Resources	Processor Time	00:00:00,19
	Elapsed Time	00:00:00,00

Case Processing Summary

Unweighted Cases[a]		N	Percent
Selected Cases	Included in Analysis	4198	90.2
	Missing Cases	458	9.8
	Total	4656	100.0
Unselected Cases		0	.0
Total		4656	100.0

a. If weight is in effect, see classification table for the total number of cases.

Dependent Variable Encoding

Original Value	Internal Value
Nej	0
Ja	1

Categorical Variables Codings

			Parameter coding		
		Frequency	(1)	(2)	(3)
Cluster_model_men	1.00	1809	.000	.000	.000
	2.00	1807	1.000	.000	.000
	3.00	336	.000	1.000	.000
	4.00	246	.000	.000	1.000

Block 0: Beginning Block

Classification Table[a,b]

Observed			Predicted: Self-harm_ny Nej	Predicted: Self-harm_ny Ja	Percentage Correct
Step 0	Self-harm_ny	Nej	3845	0	100.0
		Ja	353	0	.0
	Overall Percentage				91.6

a. Constant is included in the model.

b. The cut value is ,500

Variables in the Equation

		B	S.E.	Wald	df	Sig.	Exp(B)
Step 0	Constant	-2.388	.056	1843.824	1	.000	.092

Variables not in the Equation

			Score	df	Sig.
Step 0	Variables	Cluster_model_men	314.284	3	<.001
		Cluster_model_men(1)	1.275	1	.259
		Cluster_model_men(2)	80.388	1	<.001
		Cluster_model_men(3)	165.400	1	<.001
	Overall Statistics		314.284	3	<.001

Block 1: Method = Enter

Omnibus Tests of Model Coefficients

		Chi-square	df	Sig.
Step 1	Step	267.309	3	<.001
	Block	267.309	3	<.001
	Model	267.309	3	<.001

Model Summary

Step	-2 Log likelihood	Cox & Snell R Square	Nagelkerke R Square
1	2156.121[a]	.062	.141

a. Estimation terminated at iteration number 6 because parameter estimates changed by less than ,001.

Classification Table[a]

Observed			Predicted: Self-harm_ny Nej	Predicted: Self-harm_ny Ja	Percentage Correct
Step 1	Self-harm_ny	Nej	3845	0	100.0
		Ja	353	0	.0
	Overall Percentage				91.6

a. The cut value is ,500

Variables in the Equation

		B	S.E.	Wald	df	Sig.	Exp(B)	95% C.I.for EXP(B) Lower	95% C.I.for EXP(B) Upper
Step 1[a]	Cluster_model_men			236.468	3	<.001			
	Cluster_model_men(1)	1.374	.173	62.756	1	<.001	3.950	2.812	5.550
	Cluster_model_men(2)	2.392	.202	139.703	1	<.001	10.940	7.357	16.267
	Cluster_model_men(3)	2.868	.206	193.591	1	<.001	17.594	11.747	26.350
	Constant	-3.692	.153	585.080	1	<.001	.025		

a. Variable(s) entered on step 1: Cluster_model_men.

Logistic Regression

Notes

Output Created		26-AUG-2025 11:44:24
Comments		
Input	Data	/Users/stevenlc/Library/CloudStorage/OneDrive-Privat/ICloud filer/Doktorander/Rickard/Artikel 4/Menfour class model.sav
	Active Dataset	DataSet3
	File Label	Scored Data File
	Filter	<none>
	Weight	<none>
	Split File	<none>

	N of Rows in Working Data File	4656
Missing Value Handling	Definition of Missing	User-defined missing values are treated as missing
Syntax		LOGISTIC REGRESSION VARIABLES Symtom_score_måttlig_ny /METHOD=ENTER Cluster_model_men /CONTRAST (Cluster_model_men)=INDICATOR(1) /PRINT=CI(95) /CRITERIA=PIN(0.05) POUT(0.10) ITERATE(20) CUT(0.5).
Resources	Processor Time	00:00:00,19
	Elapsed Time	00:00:00,00

Case Processing Summary

Unweighted Cases[a]		N	Percent
Selected Cases	Included in Analysis	3907	83.9
	Missing Cases	749	16.1
	Total	4656	100.0
Unselected Cases		0	.0
Total		4656	100.0

a. If weight is in effect, see classification table for the total number of cases.

Dependent Variable Encoding

Original Value	Internal Value
Nej	0
Ja	1

Categorical Variables Codings

			Parameter coding		
		Frequency	(1)	(2)	(3)
Cluster_model_men	1.00	1666	.000	.000	.000
	2.00	1700	1.000	.000	.000
	3.00	314	.000	1.000	.000
	4.00	227	.000	.000	1.000

Block 0: Beginning Block

Classification Table[a,b]

Observed			Predicted: Somatization_ny Nej	Predicted: Somatization_ny Ja	Predicted: Percentage Correct
Step 0	Somatization_ny	Nej	3765	0	100.0
		Ja	142	0	.0
	Overall Percentage				96.4

a. Constant is included in the model.

b. The cut value is ,500

Variables in the Equation

		B	S.E.	Wald	df	Sig.	Exp(B)
Step 0	Constant	-3.278	.085	1470.083	1	.000	.038

Variables not in the Equation

			Score	df	Sig.
Step 0	Variables	Cluster_model_men	78.493	3	<.001
		Cluster_model_men(1)	7.410	1	.006
		Cluster_model_men(2)	21.041	1	<.001
		Cluster_model_men(3)	52.086	1	<.001
	Overall Statistics		78.493	3	<.001

Block 1: Method = Enter

Omnibus Tests of Model Coefficients

		Chi-square	df	Sig.
Step 1	Step	56.293	3	<.001
	Block	56.293	3	<.001
	Model	56.293	3	<.001

Model Summary

Step	-2 Log likelihood	Cox & Snell R Square	Nagelkerke R Square
1	1163.856[a]	.014	.053

a. Estimation terminated at iteration number 6 because parameter estimates changed by less than ,001.

Classification Table[a]

	Observed		Predicted: Somatization_ny Nej	Predicted: Somatization_ny Ja	Predicted: Percentage Correct
Step 1	Somatization_ny	Nej	3765	0	100.0
		Ja	142	0	.0
	Overall Percentage				96.4

a. The cut value is ,500

Variables in the Equation

		B	S.E.	Wald	df	Sig.	Exp(B)	95% C.I.for EXP(B) Lower	95% C.I.for EXP(B) Upper
Step 1[a]	Cluster_model_men			66.383	3	<.001			
	Cluster_model_men(1)	.073	.216	.113	1	.737	1.075	.704	1.643
	Cluster_model_men(2)	1.250	.258	23.550	1	<.001	3.491	2.107	5.783
	Cluster_model_men(3)	1.694	.255	44.030	1	<.001	5.441	3.299	8.973
	Constant	-3.655	.156	546.928	1	<.001	.026		

a. Variable(s) entered on step 1: Cluster_model_men.

Logistic Regression

Notes

Output Created		26-AUG-2025 11:44:24
Comments		
Input	Data	/Users/stevenlc/Library/CloudStorage/OneDrive-Privat/ICloud filer/Doktorander/Rickard/Artikel 4/Menfour class model.sav
	Active Dataset	DataSet3
	File Label	Scored Data File

	Filter	<none>
	Weight	<none>
	Split File	<none>
	N of Rows in Working Data File	4656
Missing Value Handling	Definition of Missing	User-defined missing values are treated as missing
Syntax		LOGISTIC REGRESSION VARIABLES IBS_ny /METHOD=ENTER Cluster_model_men /CONTRAST (Cluster_model_men)=INDICATOR(1) /PRINT=CI(95) /CRITERIA=PIN(0.05) POUT(0.10) ITERATE(20) CUT(0.5).
Resources	Processor Time	00:00:00,21
	Elapsed Time	00:00:00,00

Case Processing Summary

Unweighted Cases[a]		N	Percent
Selected Cases	Included in Analysis	4222	90.7
	Missing Cases	434	9.3
	Total	4656	100.0
Unselected Cases		0	.0
Total		4656	100.0

a. If weight is in effect, see classification table for the total number of cases.

Dependent Variable Encoding

Original Value	Internal Value
Nej	0
Ja	1

Categorical Variables Codings

			Parameter coding		
		Frequency	(1)	(2)	(3)
Cluster_model_men	1.00	1818	.000	.000	.000
	2.00	1817	1.000	.000	.000

	3.00	340	.000	1.000	.000
	4.00	247	.000	.000	1.000

Block 0: Beginning Block

Classification Table[a,b]

Observed			Predicted IBS_ny Nej	Predicted IBS_ny Ja	Predicted Percentage Correct
Step 0	IBS_ny	Nej	4106	0	100.0
		Ja	116	0	.0
	Overall Percentage				97.3

a. Constant is included in the model.

b. The cut value is ,500

Variables in the Equation

		B	S.E.	Wald	df	Sig.	Exp(B)
Step 0	Constant	-3.567	.094	1435.063	1	.000	.028

Variables not in the Equation

			Score	df	Sig.
Step 0	Variables	Cluster_model_men	13.795	3	.003
		Cluster_model_men(1)	.342	1	.558
		Cluster_model_men(2)	5.308	1	.021
		Cluster_model_men(3)	4.374	1	.036
	Overall Statistics		13.795	3	.003

Block 1: Method = Enter

Omnibus Tests of Model Coefficients

		Chi-square	df	Sig.
Step 1	Step	12.724	3	.005
	Block	12.724	3	.005
	Model	12.724	3	.005

Model Summary

Step	-2 Log likelihood	Cox & Snell R Square	Nagelkerke R Square
1	1049.977[a]	.003	.014

a. Estimation terminated at iteration number 7 because parameter estimates changed by less than ,001.

Classification Table[a]

Observed			Predicted IBS_ny Nej	Predicted IBS_ny Ja	Predicted Percentage Correct
Step 1	IBS_ny	Nej	4106	0	100.0
		Ja	116	0	.0
	Overall Percentage				97.3

a. The cut value is ,500

Variables in the Equation

		B	S.E.	Wald	df	Sig.	Exp(B)	95% C.I.for EXP(B) Lower	95% C.I.for EXP(B) Upper
Step 1[a]	Cluster_model_men			13.187	3	.004			
	Cluster_model_men(1)	.426	.220	3.731	1	.053	1.531	.994	2.357
	Cluster_model_men(2)	.923	.308	8.986	1	.003	2.516	1.376	4.599
	Cluster_model_men(3)	.956	.342	7.831	1	.005	2.601	1.332	5.082
	Constant	-3.931	.171	530.355	1	<.001	.020		

a. Variable(s) entered on step 1: Cluster_model_men.

Logistic Regression

Notes

Output Created		26-AUG-2025 11:44:24
Comments		
Input	Data	/Users/stevenlc/Library/CloudStorage/OneDrive-Privat/ICloud filer/Doktorander/Rickard/Artikel 4/Menfour class model.sav

	Active Dataset	DataSet3
	File Label	Scored Data File
	Filter	<none>
	Weight	<none>
	Split File	<none>
	N of Rows in Working Data File	4656
Missing Value Handling	Definition of Missing	User-defined missing values are treated as missing
Syntax		LOGISTIC REGRESSION VARIABLES Fibromyalgi_ny /METHOD=ENTER Cluster_model_men /CONTRAST (Cluster_model_men)=INDICATOR(1) /PRINT=CI(95) /CRITERIA=PIN(0.05) POUT(0.10) ITERATE(20) CUT(0.5).
Resources	Processor Time	00:00:00,20
	Elapsed Time	00:00:01,00

Case Processing Summary

Unweighted Cases[a]		N	Percent
Selected Cases	Included in Analysis	4222	90.7
	Missing Cases	434	9.3
	Total	4656	100.0
Unselected Cases		0	.0
Total		4656	100.0

a. If weight is in effect, see classification table for the total number of cases.

Dependent Variable Encoding

Original Value	Internal Value
Nej	0
Ja	1

Categorical Variables Codings

	Frequency	Parameter coding

			(1)	(2)	(3)
Cluster_model_men	1.00	1818	.000	.000	.000
	2.00	1817	1.000	.000	.000
	3.00	340	.000	1.000	.000
	4.00	247	.000	.000	1.000

Block 0: Beginning Block

Classification Table[a,b]

Observed			Predicted		
			Fibromyalgia_ny		Percentage Correct
			Nej	Ja	
Step 0	Fibromyalgia_ny	Nej	4194	0	100.0
		Ja	28	0	.0
	Overall Percentage				99.3

a. Constant is included in the model.
b. The cut value is ,500

Variables in the Equation

		B	S.E.	Wald	df	Sig.	Exp(B)
Step 0	Constant	-5.009	.190	697.921	1	<.001	.007

Variables not in the Equation

			Score	df	Sig.
Step 0	Variables	Cluster_model_men	1.594	3	.661
		Cluster_model_men(1)	.616	1	.432
		Cluster_model_men(2)	.032	1	.859
		Cluster_model_men(3)	1.211	1	.271
	Overall Statistics		1.594	3	.661

Block 1: Method = Enter

Omnibus Tests of Model Coefficients

	Chi-square	df	Sig.

Step 1	Step	1.390	3	.708
	Block	1.390	3	.708
	Model	1.390	3	.708

Model Summary

Step	-2 Log likelihood	Cox & Snell R Square	Nagelkerke R Square
1	335.312[a]	.000	.004

a. Estimation terminated at iteration number 8 because parameter estimates changed by less than ,001.

Classification Table[a]

Observed			Predicted Fibromyalgia_ny Nej	Predicted Fibromyalgia_ny Ja	Percentage Correct
Step 1	Fibromyalgia_ny	Nej	4194	0	100.0
		Ja	28	0	.0
	Overall Percentage				99.3

a. The cut value is ,500

Variables in the Equation

		B	S.E.	Wald	df	Sig.	Exp(B)	95% C.I.for EXP(B) Lower	95% C.I.for EXP(B) Upper
Step 1[a]	Cluster_model_men			1.542	3	.673			
	Cluster_model_men(1)	-.263	.422	.390	1	.532	.768	.336	1.757
	Cluster_model_men(2)	-.197	.762	.067	1	.796	.822	.185	3.657
	Cluster_model_men(3)	.535	.644	.689	1	.406	1.707	.483	6.033
	Constant	-4.933	.278	314.133	1	<.001	.007		

a. Variable(s) entered on step 1: Cluster_model_men.

Logistic Regression

Notes

Output Created	26-AUG-2025 11:44:25
Comments	

Input	Data	/Users/stevenlc/Library/CloudStorage/OneDrive-Privat/ICloud filer/Doktorander/Rickard/Artikel 4/Menfour class model.sav
	Active Dataset	DataSet3
	File Label	Scored Data File
	Filter	<none>
	Weight	<none>
	Split File	<none>
	N of Rows in Working Data File	4656
Missing Value Handling	Definition of Missing	User-defined missing values are treated as missing
Syntax		LOGISTIC REGRESSION VARIABLES Ischemic_HD_ny /METHOD=ENTER Cluster_model_men /CONTRAST (Cluster_model_men)=INDICATOR(1) /PRINT=CI(95) /CRITERIA=PIN(0.05) POUT(0.10) ITERATE(20) CUT(0.5).
Resources	Processor Time	00:00:00,19
	Elapsed Time	00:00:00,00

Case Processing Summary

Unweighted Cases[a]		N	Percent
Selected Cases	Included in Analysis	4222	90.7
	Missing Cases	434	9.3
	Total	4656	100.0
Unselected Cases		0	.0
Total		4656	100.0

a. If weight is in effect, see classification table for the total number of cases.

Dependent Variable Encoding

Original Value	Internal Value

Nej	0
Ja	1

Categorical Variables Codings

		Frequency	Parameter coding (1)	(2)	(3)
Cluster_model_men	1.00	1818	.000	.000	.000
	2.00	1817	1.000	.000	.000
	3.00	340	.000	1.000	.000
	4.00	247	.000	.000	1.000

Block 0: Beginning Block

Classification Table[a,b]

Observed			Predicted IHD_ny Nej	Ja	Percentage Correct
Step 0	IHD_ny	Nej	3920	0	100.0
		Ja	302	0	.0
	Overall Percentage				92.8

a. Constant is included in the model.

b. The cut value is ,500

Variables in the Equation

		B	S.E.	Wald	df	Sig.	Exp(B)
Step 0	Constant	-2.563	.060	1842.529	1	.000	.077

Variables not in the Equation

			Score	df	Sig.
Step 0	Variables	Cluster_model_men	18.462	3	<.001
		Cluster_model_men(1)	13.067	1	<.001
		Cluster_model_men(2)	.084	1	.772
		Cluster_model_men(3)	.871	1	.351
	Overall Statistics		18.462	3	<.001

Block 1: Method = Enter

Omnibus Tests of Model Coefficients

		Chi-square	df	Sig.
Step 1	Step	18.382	3	<.001
	Block	18.382	3	<.001
	Model	18.382	3	<.001

Model Summary

Step	-2 Log likelihood	Cox & Snell R Square	Nagelkerke R Square
1	2156.614[a]	.004	.011

a. Estimation terminated at iteration number 5 because parameter estimates changed by less than ,001.

Classification Table[a]

Observed			Predicted IHD_ny Nej	Predicted IHD_ny Ja	Percentage Correct
Step 1	IHD_ny	Nej	3920	0	100.0
		Ja	302	0	.0
	Overall Percentage				92.8

a. The cut value is ,500

Variables in the Equation

		B	S.E.	Wald	df	Sig.	Exp(B)	95% C.I.for EXP(B) Lower	95% C.I.for EXP(B) Upper
Step 1[a]	Cluster_model_men			18.132	3	<.001			
	Cluster_model_men(1)	-.539	.131	16.829	1	<.001	.583	.451	.755
	Cluster_model_men(2)	-.319	.231	1.909	1	.167	.727	.462	1.143
	Cluster_model_men(3)	-.508	.287	3.127	1	.077	.602	.343	1.057
	Constant	-2.304	.082	796.671	1	<.001	.100		

a. Variable(s) entered on step 1: Cluster_model_men.

Logistic Regression

Notes

Output Created		26-AUG-2025 11:44:25
Comments		
Input	Data	/Users/stevenlc/Library/CloudStorage/OneDrive-Privat/ICloud filer/Doktorander/Rickard/Artikel 4/Menfour class model.sav
	Active Dataset	DataSet3
	File Label	Scored Data File
	Filter	<none>
	Weight	<none>
	Split File	<none>
	N of Rows in Working Data File	4656
Missing Value Handling	Definition of Missing	User-defined missing values are treated as missing
Syntax		LOGISTIC REGRESSION VARIABLES KOL_ny /METHOD=ENTER Cluster_model_men /CONTRAST (Cluster_model_men)=INDICATOR(1) /PRINT=CI(95) /CRITERIA=PIN(0.05) POUT(0.10) ITERATE(20) CUT(0.5).
Resources	Processor Time	00:00:00,20
	Elapsed Time	00:00:00,00

Case Processing Summary

Unweighted Cases[a]		N	Percent
Selected Cases	Included in Analysis	4222	90.7
	Missing Cases	434	9.3
	Total	4656	100.0
Unselected Cases		0	.0
Total		4656	100.0

a. If weight is in effect, see classification table for the total number of cases.

Dependent Variable Encoding

Original Value	Internal Value
Nej	0
Ja	1

Categorical Variables Codings

			Parameter coding		
		Frequency	(1)	(2)	(3)
Cluster_model_men	1.00	1818	.000	.000	.000
	2.00	1817	1.000	.000	.000
	3.00	340	.000	1.000	.000
	4.00	247	.000	.000	1.000

Block 0: Beginning Block

Classification Table[a,b]

			Predicted		
			COPD_ny		Percentage Correct
Observed			Nej	Ja	
Step 0	COPD_ny	Nej	4151	0	100.0
		Ja	71	0	.0
	Overall Percentage				98.3

a. Constant is included in the model.

b. The cut value is ,500

Variables in the Equation

		B	S.E.	Wald	df	Sig.	Exp(B)
Step 0	Constant	-4.068	.120	1155.435	1	<.001	.017

Variables not in the Equation

			Score	df	Sig.
Step 0	Variables	Cluster_model_men	5.135	3	.162
		Cluster_model_men(1)	1.804	1	.179
		Cluster_model_men(2)	3.548	1	.060
		Cluster_model_men(3)	.887	1	.346
	Overall Statistics		5.135	3	.162

Block 1: Method = Enter

Omnibus Tests of Model Coefficients

		Chi-square	df	Sig.
Step 1	Step	4.511	3	.211
	Block	4.511	3	.211
	Model	4.511	3	.211

Model Summary

Step	-2 Log likelihood	Cox & Snell R Square	Nagelkerke R Square
1	716.412[a]	.001	.007

a. Estimation terminated at iteration number 7 because parameter estimates changed by less than ,001.

Classification Table[a]

	Observed		Predicted		
			COPD_ny		Percentage Correct
			Nej	Ja	
Step 1	COPD_ny	Nej	4151	0	100.0
		Ja	71	0	.0
	Overall Percentage				98.3

a. The cut value is ,500

Variables in the Equation

		B	S.E.	Wald	df	Sig.	Exp(B)	95% C.I.for EXP(B) Lower	Upper
Step 1[a]	Cluster_model_men			4.968	3	.174			
	Cluster_model_men(1)	-.185	.273	.457	1	.499	.831	.487	1.419
	Cluster_model_men(2)	.591	.370	2.552	1	.110	1.806	.875	3.730
	Cluster_model_men(3)	.395	.452	.761	1	.383	1.484	.611	3.602
	Constant	-4.088	.184	492.996	1	<.001	.017		

a. Variable(s) entered on step 1: Cluster_model_men.

Logistic Regression

Notes

Output Created		26-AUG-2025 11:44:25
Comments		
Input	Data	/Users/stevenlc/Library/CloudStorage/OneDrive-Privat/ICloud filer/Doktorander/Rickard/Artikel 4/Menfour class model.sav
	Active Dataset	DataSet3
	File Label	Scored Data File
	Filter	<none>
	Weight	<none>
	Split File	<none>
	N of Rows in Working Data File	4656
Missing Value Handling	Definition of Missing	User-defined missing values are treated as missing
Syntax		LOGISTIC REGRESSION VARIABLES Diabetes_II_ny /METHOD=ENTER Cluster_model_men /CONTRAST (Cluster_model_men)=INDICATOR(1) /PRINT=CI(95) /CRITERIA=PIN(0.05) POUT(0.10) ITERATE(20) CUT(0.5).
Resources	Processor Time	00:00:00,20
	Elapsed Time	00:00:00,00

Case Processing Summary

Unweighted Cases[a]		N	Percent
Selected Cases	Included in Analysis	4222	90.7
	Missing Cases	434	9.3
	Total	4656	100.0
Unselected Cases		0	.0
Total		4656	100.0

a. If weight is in effect, see classification table for the total number of cases.

Dependent Variable Encoding

Original Value	Internal Value
Nej	0
Ja	1

Categorical Variables Codings

		Frequency	Parameter coding (1)	(2)	(3)
Cluster_model_men	1.00	1818	.000	.000	.000
	2.00	1817	1.000	.000	.000
	3.00	340	.000	1.000	.000
	4.00	247	.000	.000	1.000

Block 0: Beginning Block

Classification Table[a,b]

Observed			Predicted: Diabetes type II_ny Nej	Ja	Percentage Correct
Step 0	Diabetes type II_ny	Nej	4027	0	100.0
		Ja	195	0	.0
	Overall Percentage				95.4

a. Constant is included in the model.

b. The cut value is ,500

Variables in the Equation

		B	S.E.	Wald	df	Sig.	Exp(B)
Step 0	Constant	-3.028	.073	1705.084	1	.000	.048

Variables not in the Equation

			Score	df	Sig.
Step 0	Variables	Cluster_model_men	13.407	3	.004

Cluster_model_men(1)	12.550	1	<.001
Cluster_model_men(2)	1.340	1	.247
Cluster_model_men(3)	.016	1	.899
Overall Statistics	13.407	3	.004

Block 1: Method = Enter

Omnibus Tests of Model Coefficients

		Chi-square	df	Sig.
Step 1	Step	13.732	3	.003
	Block	13.732	3	.003
	Model	13.732	3	.003

Model Summary

Step	-2 Log likelihood	Cox & Snell R Square	Nagelkerke R Square
1	1566.395[a]	.003	.010

a. Estimation terminated at iteration number 6 because parameter estimates changed by less than ,001.

Classification Table[a]

Observed			Predicted: Diabetes type II_ny Nej	Predicted: Diabetes type II_ny Ja	Percentage Correct
Step 1	Diabetes type II_ny	Nej	4027	0	100.0
		Ja	195	0	.0
	Overall Percentage				95.4

a. The cut value is ,500

Variables in the Equation

		B	S.E.	Wald	df	Sig.	Exp(B)	95% C.I.for EXP(B) Lower	95% C.I.for EXP(B) Upper
Step 1[a]	Cluster_model_men			13.096	3	.004			
	Cluster_model_men(1)	-.575	.166	12.044	1	<.001	.563	.407	.779
	Cluster_model_men(2)	.030	.252	.014	1	.906	1.030	.629	1.687
	Cluster_model_men(3)	-.264	.325	.660	1	.416	.768	.407	1.451
	Constant	-2.802	.101	769.922	1	<.001	.061		

a. Variable(s) entered on step 1: Cluster_model_men.

Logistic Regression

Notes

Output Created		26-AUG-2025 11:44:25
Comments		
Input	Data	/Users/stevenlc/Library/CloudStorage/OneDrive-Privat/ICloud filer/Doktorander/Rickard/Artikel 4/Menfour class model.sav
	Active Dataset	DataSet3
	File Label	Scored Data File
	Filter	<none>
	Weight	<none>
	Split File	<none>
	N of Rows in Working Data File	4656
Missing Value Handling	Definition of Missing	User-defined missing values are treated as missing
Syntax		LOGISTIC REGRESSION VARIABLES Tumörsjd_ny /METHOD=ENTER Cluster_model_men /CONTRAST (Cluster_model_men)=INDICATOR(1) /PRINT=CI(95) /CRITERIA=PIN(0.05) POUT(0.10) ITERATE(20) CUT(0.5).
Resources	Processor Time	00:00:00,20
	Elapsed Time	00:00:00,00

Case Processing Summary

Unweighted Cases[a]		N	Percent
Selected Cases	Included in Analysis	4222	90.7
	Missing Cases	434	9.3
	Total	4656	100.0

Unselected Cases	0	.0
Total	4656	100.0

a. If weight is in effect, see classification table for the total number of cases.

Dependent Variable Encoding

Original Value	Internal Value
Nej	0
Ja	1

Categorical Variables Codings

			Parameter coding		
		Frequency	(1)	(2)	(3)
Cluster_model_men	1.00	1818	.000	.000	.000
	2.00	1817	1.000	.000	.000
	3.00	340	.000	1.000	.000
	4.00	247	.000	.000	1.000

Block 0: Beginning Block

Classification Table[a,b]

			Predicted		
			Cancer_ny		Percentage
	Observed		Nej	Ja	Correct
Step 0	Cancer_ny	Nej	4059	0	100.0
		Ja	163	0	.0
	Overall Percentage				96.1

a. Constant is included in the model.

b. The cut value is ,500

Variables in the Equation

		B	S.E.	Wald	df	Sig.	Exp(B)
Step 0	Constant	-3.215	.080	1619.700	1	.000	.040

Variables not in the Equation

			Score	df	Sig.
Step 0	Variables	Cluster_model_men	6.547	3	.088
		Cluster_model_men(1)	.448	1	.503
		Cluster_model_men(2)	2.973	1	.085
		Cluster_model_men(3)	3.551	1	.060
	Overall Statistics		6.547	3	.088

Block 1: Method = Enter

Omnibus Tests of Model Coefficients

		Chi-square	df	Sig.
Step 1	Step	7.134	3	.068
	Block	7.134	3	.068
	Model	7.134	3	.068

Model Summary

Step	-2 Log likelihood	Cox & Snell R Square	Nagelkerke R Square
1	1373.397[a]	.002	.006

a. Estimation terminated at iteration number 7 because parameter estimates changed by less than ,001.

Classification Table[a]

Observed			Predicted: Cancer_ny Nej	Predicted: Cancer_ny Ja	Predicted: Percentage Correct
Step 1	Cancer_ny	Nej	4059	0	100.0
		Ja	163	0	.0
	Overall Percentage				96.1

a. The cut value is ,500

Variables in the Equation

		B	S.E.	Wald	df	Sig.	Exp(B)	95% C.I.for EXP(B) Lower	95% C.I.for EXP(B) Upper
Step 1[a]	Cluster_model_men			6.229	3	.101			
	Cluster_model_men(1)	-.118	.173	.470	1	.493	.888	.633	1.246
	Cluster_model_men(2)	.333	.264	1.587	1	.208	1.395	.831	2.342
	Cluster_model_men(3)	-.947	.518	3.343	1	.067	.388	.141	1.071

Constant	-3.160	.119	708.799	1	<.001	.042		

a. Variable(s) entered on step 1: Cluster_model_men.

Logistic Regression

Notes

Output Created		26-AUG-2025 11:44:25
Comments		
Input	Data	/Users/stevenlc/Library/CloudStorage/OneDrive-Privat/ICloud filer/Doktorander/Rickard/Artikel 4/Menfour class model.sav
	Active Dataset	DataSet3
	File Label	Scored Data File
	Filter	<none>
	Weight	<none>
	Split File	<none>
	N of Rows in Working Data File	4656
Missing Value Handling	Definition of Missing	User-defined missing values are treated as missing
Syntax		LOGISTIC REGRESSION VARIABLES Fetma_ny /METHOD=ENTER Cluster_model_men /CONTRAST (Cluster_model_men)=INDICATOR(1) /PRINT=CI(95) /CRITERIA=PIN(0.05) POUT(0.10) ITERATE(20) CUT(0.5).
Resources	Processor Time	00:00:00,19
	Elapsed Time	00:00:01,00

Case Processing Summary

Unweighted Cases[a]		N	Percent
Selected Cases	Included in Analysis	4166	89.5

	Missing Cases	490	10.5
	Total	4656	100.0
Unselected Cases		0	.0
Total		4656	100.0

a. If weight is in effect, see classification table for the total number of cases.

Dependent Variable Encoding

Original Value	Internal Value
Nej	0
Ja	1

Categorical Variables Codings

		Frequency	Parameter coding (1)	(2)	(3)
Cluster_model_men	1.00	1788	.000	.000	.000
	2.00	1796	1.000	.000	.000
	3.00	338	.000	1.000	.000
	4.00	244	.000	.000	1.000

Block 0: Beginning Block

Classification Table[a,b]

Observed			Predicted Obesity_ny Nej	Ja	Percentage Correct
Step 0	Obesity_ny	Nej	3567	0	100.0
		Ja	599	0	.0
	Overall Percentage				85.6

a. Constant is included in the model.
b. The cut value is ,500

Variables in the Equation

		B	S.E.	Wald	df	Sig.	Exp(B)
Step 0	Constant	-1.784	.044	1632.701	1	.000	.168

Variables not in the Equation

			Score	df	Sig.
Step 0	Variables	Cluster_model_men	6.538	3	.088
		Cluster_model_men(1)	4.669	1	.031
		Cluster_model_men(2)	3.400	1	.065
		Cluster_model_men(3)	.000	1	.988
	Overall Statistics		6.538	3	.088

Block 1: Method = Enter

Omnibus Tests of Model Coefficients

		Chi-square	df	Sig.
Step 1	Step	6.419	3	.093
	Block	6.419	3	.093
	Model	6.419	3	.093

Model Summary

Step	-2 Log likelihood	Cox & Snell R Square	Nagelkerke R Square
1	3424.463[a]	.002	.003

a. Estimation terminated at iteration number 4 because parameter estimates changed by less than ,001.

Classification Table[a]

Observed			Predicted: Obesity_ny Nej	Predicted: Obesity_ny Ja	Predicted: Percentage Correct
Step 1	Obesity_ny	Nej	3567	0	100.0
		Ja	599	0	.0
	Overall Percentage				85.6

a. The cut value is ,500

Variables in the Equation

		B	S.E.	Wald	df	Sig.	Exp(B)	95% C.I.for EXP(B) Lower	95% C.I.for EXP(B) Upper
Step 1[a]	Cluster_model_men			6.509	3	.089			

Cluster_model_men(1)	-.172	.096	3.177	1	.075	.842	.697	1.017
Cluster_model_men(2)	.193	.157	1.520	1	.218	1.213	.892	1.650
Cluster_model_men(3)	-.060	.194	.096	1	.756	.942	.643	1.378
Constant	-1.727	.066	683.464	1	<.001	.178		

a. Variable(s) entered on step 1: Cluster_model_men.

Logistic Regression

Notes

Output Created		26-AUG-2025 11:44:26
Comments		
Input	Data	/Users/stevenlc/Library/CloudStorage/OneDrive-Privat/ICloud filer/Doktorander/Rickard/Artikel 4/Menfour class model.sav
	Active Dataset	DataSet3
	File Label	Scored Data File
	Filter	<none>
	Weight	<none>
	Split File	<none>
	N of Rows in Working Data File	4656
Missing Value Handling	Definition of Missing	User-defined missing values are treated as missing
Syntax		LOGISTIC REGRESSION VARIABLES Storrökare_ny /METHOD=ENTER Cluster_model_men /CONTRAST (Cluster_model_men)=INDICATOR(1) /PRINT=CI(95) /CRITERIA=PIN(0.05) POUT(0.10) ITERATE(20) CUT(0.5).
Resources	Processor Time	00:00:00,19
	Elapsed Time	00:00:00,00

Case Processing Summary

Unweighted Cases[a]		N	Percent
Selected Cases	Included in Analysis	4172	89.6
	Missing Cases	484	10.4
	Total	4656	100.0
Unselected Cases		0	.0
Total		4656	100.0

a. If weight is in effect, see classification table for the total number of cases.

Dependent Variable Encoding

Original Value	Internal Value
Nej	0
Ja	1

Categorical Variables Codings

		Frequency	Parameter coding (1)	(2)	(3)
Cluster_model_men	1.00	1791	.000	.000	.000
	2.00	1798	1.000	.000	.000
	3.00	339	.000	1.000	.000
	4.00	244	.000	.000	1.000

Block 0: Beginning Block

Classification Table[a,b]

Observed			Predicted: Heavy smoking_ny Nej	Ja	Percentage Correct
Step 0	Heavy smoking_ny	Nej	3941	0	100.0
		Ja	231	0	.0
	Overall Percentage				94.5

a. Constant is included in the model.

b. The cut value is ,500

Variables in the Equation

	B	S.E.	Wald	df	Sig.	Exp(B)

Step 0	Constant	-2.837	.068	1755.994	1	.000	.059

Variables not in the Equation

			Score	df	Sig.
Step 0	Variables	Cluster_model_men	17.931	3	<.001
		Cluster_model_men(1)	.236	1	.627
		Cluster_model_men(2)	16.170	1	<.001
		Cluster_model_men(3)	.516	1	.473
	Overall Statistics		17.931	3	<.001

Block 1: Method = Enter

Omnibus Tests of Model Coefficients

		Chi-square	df	Sig.
Step 1	Step	15.172	3	.002
	Block	15.172	3	.002
	Model	15.172	3	.002

Model Summary

Step	-2 Log likelihood	Cox & Snell R Square	Nagelkerke R Square
1	1770.700[a]	.004	.010

a. Estimation terminated at iteration number 6 because parameter estimates changed by less than ,001.

Classification Table[a]

			Predicted		
			Heavy smoking_ny		Percentage Correct
	Observed		Nej	Ja	
Step 1	Heavy smoking_ny	Nej	3941	0	100.0
		Ja	231	0	.0
	Overall Percentage				94.5

a. The cut value is ,500

Variables in the Equation

	B	S.E.	Wald	df	Sig.	Exp(B)	95% C.I.for EXP(B)

								Lower	Upper
Step 1[a]	Cluster_model_men			17.185	3	<.001			
	Cluster_model_men(1)	.136	.153	.793	1	.373	1.146	.849	1.548
	Cluster_model_men(2)	.850	.211	16.290	1	<.001	2.340	1.548	3.535
	Cluster_model_men(3)	.355	.282	1.587	1	.208	1.426	.821	2.477
	Constant	-3.012	.112	726.162	1	<.001	.049		

a. Variable(s) entered on step 1: Cluster_model_men.

Logistic Regression

Notes

Output Created		26-AUG-2025 11:44:26
Comments		
Input	Data	/Users/stevenlc/Library/CloudStorage/OneDrive-Privat/ICloud filer/Doktorander/Rickard/Artikel 4/Menfour class model.sav
	Active Dataset	DataSet3
	File Label	Scored Data File
	Filter	<none>
	Weight	<none>
	Split File	<none>
	N of Rows in Working Data File	4656
Missing Value Handling	Definition of Missing	User-defined missing values are treated as missing
Syntax		LOGISTIC REGRESSION VARIABLES AUDITRISK_inknykt_ny /METHOD=ENTER Cluster_model_men /CONTRAST (Cluster_model_men)=INDICATOR(1) /PRINT=CI(95) /CRITERIA=PIN(0.05) POUT(0.10) ITERATE(20) CUT(0.5).
Resources	Processor Time	00:00:00,19

Elapsed Time	00:00:00,00

Case Processing Summary

Unweighted Cases[a]		N	Percent
Selected Cases	Included in Analysis	4106	88.2
	Missing Cases	550	11.8
	Total	4656	100.0
Unselected Cases		0	.0
Total		4656	100.0

a. If weight is in effect, see classification table for the total number of cases.

Dependent Variable Encoding

Original Value	Internal Value
Nej	0
Ja	1

Categorical Variables Codings

			Parameter coding		
		Frequency	(1)	(2)	(3)
Cluster_model_men	1.00	1746	.000	.000	.000
	2.00	1782	1.000	.000	.000
	3.00	335	.000	1.000	.000
	4.00	243	.000	.000	1.000

Block 0: Beginning Block

Classification Table[a,b]

			Predicted		
			Heavy drinking_ny		Percentage Correct
Observed			Nej	Ja	
Step 0	Heavy drinking_ny	Nej	3246	0	100.0
		Ja	860	0	.0
	Overall Percentage				79.1

a. Constant is included in the model.

b. The cut value is ,500

Variables in the Equation

		B	S.E.	Wald	df	Sig.	Exp(B)
Step 0	Constant	-1.328	.038	1199.459	1	<.001	.265

Variables not in the Equation

			Score	df	Sig.
Step 0	Variables	Cluster_model_men	130.700	3	<.001
		Cluster_model_men(1)	23.585	1	<.001
		Cluster_model_men(2)	21.162	1	<.001
		Cluster_model_men(3)	38.354	1	<.001
	Overall Statistics		130.700	3	<.001

Block 1: Method = Enter

Omnibus Tests of Model Coefficients

		Chi-square	df	Sig.
Step 1	Step	130.681	3	<.001
	Block	130.681	3	<.001
	Model	130.681	3	<.001

Model Summary

Step	-2 Log likelihood	Cox & Snell R Square	Nagelkerke R Square
1	4083.935[a]	.031	.049

a. Estimation terminated at iteration number 4 because parameter estimates changed by less than ,001.

Classification Table[a]

Observed			Predicted: Heavy drinking_ny Nej	Predicted: Heavy drinking_ny Ja	Predicted: Percentage Correct
Step 1	Heavy drinking_ny	Nej	3246	0	100.0
		Ja	860	0	.0
	Overall Percentage				79.1

a. The cut value is ,500

Variables in the Equation

		B	S.E.	Wald	df	Sig.	Exp(B)	95% C.I.for EXP(B) Lower	Upper
Step 1[a]	Cluster_model_men			124.869	3	<.001			
	Cluster_model_men(1)	.749	.089	69.971	1	<.001	2.114	1.774	2.519
	Cluster_model_men(2)	1.064	.138	59.589	1	<.001	2.897	2.212	3.796
	Cluster_model_men(3)	1.327	.151	77.626	1	<.001	3.771	2.807	5.067
	Constant	-1.876	.071	707.832	1	<.001	.153		

a. Variable(s) entered on step 1: Cluster_model_men.

Logistic Regression

Notes

Output Created		26-AUG-2025 11:44:26
Comments		
Input	Data	/Users/stevenlc/Library/CloudStorage/OneDrive-Privat/ICloud filer/Doktorander/Rickard/Artikel 4/Menfour class model.sav
	Active Dataset	DataSet3
	File Label	Scored Data File
	Filter	<none>
	Weight	<none>
	Split File	<none>
	N of Rows in Working Data File	4656
Missing Value Handling	Definition of Missing	User-defined missing values are treated as missing

Syntax		LOGISTIC REGRESSION VARIABLES Drogmissbruk_ny /METHOD=ENTER Cluster_model_men /CONTRAST (Cluster_model_men)=INDICATOR(1) /PRINT=CI(95) /CRITERIA=PIN(0.05) POUT(0.10) ITERATE(20) CUT(0.5).
Resources	Processor Time	00:00:00,20
	Elapsed Time	00:00:00,00

Case Processing Summary

Unweighted Cases[a]		N	Percent
Selected Cases	Included in Analysis	4222	90.7
	Missing Cases	434	9.3
	Total	4656	100.0
Unselected Cases		0	.0
Total		4656	100.0

a. If weight is in effect, see classification table for the total number of cases.

Dependent Variable Encoding

Original Value	Internal Value
Nej	0
Ja	1

Categorical Variables Codings

			Parameter coding		
		Frequency	(1)	(2)	(3)
Cluster_model_men	1.00	1818	.000	.000	.000
	2.00	1817	1.000	.000	.000
	3.00	340	.000	1.000	.000
	4.00	247	.000	.000	1.000

Block 0: Beginning Block

Classification Table[a,b]

Observed			Predicted: Drug abuse_ny Nej	Ja	Percentage Correct
Step 0	Drug abuse_ny	Nej	4195	0	100.0
		Ja	27	0	.0
	Overall Percentage				99.4

a. Constant is included in the model.

b. The cut value is ,500

Variables in the Equation

		B	S.E.	Wald	df	Sig.	Exp(B)
Step 0	Constant	-5.046	.193	683.030	1	<.001	.006

Variables not in the Equation

			Score	df	Sig.
Step 0	Variables	Cluster_model_men	28.011	3	<.001
		Cluster_model_men(1)	1.992	1	.158
		Cluster_model_men(2)	.015	1	.902
		Cluster_model_men(3)	27.897	1	<.001
	Overall Statistics		28.011	3	<.001

Block 1: Method = Enter

Omnibus Tests of Model Coefficients

		Chi-square	df	Sig.
Step 1	Step	15.222	3	.002
	Block	15.222	3	.002
	Model	15.222	3	.002

Model Summary

Step	-2 Log likelihood	Cox & Snell R Square	Nagelkerke R Square
1	311.426[a]	.004	.048

a. Estimation terminated at iteration number 8 because parameter estimates changed by less than ,001.

Classification Table[a]

Observed			Predicted: Drug abuse_ny Nej	Predicted: Drug abuse_ny Ja	Predicted: Percentage Correct
Step 1	Drug abuse_ny	Nej	4195	0	100.0
		Ja	27	0	.0
	Overall Percentage				99.4

a. The cut value is ,500

Variables in the Equation

		B	S.E.	Wald	df	Sig.	Exp(B)	95% C.I.for EXP(B) Lower	95% C.I.for EXP(B) Upper
Step 1[a]	Cluster_model_men			20.773	3	<.001			
	Cluster_model_men(1)	-.118	.487	.058	1	.809	.889	.342	2.309
	Cluster_model_men(2)	.173	.784	.049	1	.825	1.189	.256	5.529
	Cluster_model_men(3)	1.906	.491	15.088	1	<.001	6.728	2.571	17.605
	Constant	-5.303	.334	251.872	1	<.001	.005		

a. Variable(s) entered on step 1: Cluster_model_men.

Logistic Regression

Notes

Output Created		26-AUG-2025 11:44:26
Comments		
Input	Data	/Users/stevenlc/Library/CloudStorage/OneDrive-Privat/ICloud filer/Doktorander/Rickard/Artikel 4/Menfour class model.sav
	Active Dataset	DataSet3
	File Label	Scored Data File
	Filter	<none>
	Weight	<none>
	Split File	<none>

	N of Rows in Working Data File	4656
Missing Value Handling	Definition of Missing	User-defined missing values are treated as missing
Syntax		LOGISTIC REGRESSION VARIABLES HAD_probable_depression_ny /METHOD=ENTER Cluster_model_men fodelselandmammapappa_ny utbildningmammapappa_ny Birthyear_decades /CONTRAST (Cluster_model_men)=Indicator(1) /CONTRAST (fodelselandmammapappa_ny)=Indicator(1) /CONTRAST (utbildningmammapappa_ny)=Indicator(1) /CONTRAST (Birthyear_decades)=Indicator(1) /PRINT=CI(95) /CRITERIA=PIN(0.05) POUT(0.10) ITERATE(20) CUT(0.5).
Resources	Processor Time	00:00:00,22
	Elapsed Time	00:00:00,00

Case Processing Summary

Unweighted Cases[a]		N	Percent
Selected Cases	Included in Analysis	3626	77.9
	Missing Cases	1030	22.1
	Total	4656	100.0
Unselected Cases		0	.0
Total		4656	100.0

a. If weight is in effect, see classification table for the total number of cases.

Dependent Variable Encoding

Original Value	Internal Value
Nej	0
Ja	1

Categorical Variables Codings

		Frequency	Parameter coding (1)	(2)	(3)	(4)	(5)
Birthyear_decades	18-27	443	.000	.000	.000	.000	.000
	28-37	581	1.000	.000	.000	.000	.000
	38-47	667	.000	1.000	.000	.000	.000
	48-57	692	.000	.000	1.000	.000	.000
	58-67	863	.000	.000	.000	1.000	.000
	68-74	380	.000	.000	.000	.000	1.000
Cluster_model_men	1.00	1550	.000	.000	.000		
	2.00	1578	1.000	.000	.000		
	3.00	296	.000	1.000	.000		
	4.00	202	.000	.000	1.000		
utbildningmammapappa_ny	Minst en förälder högre utb än grundskola	1814	.000				
	Båda föräldrarna grundskola	1812	1.000				
fodelselandmammapappa_ny	Minst en förälder född i norden	3401	.000				
	Båda födda utanför Norden	225	1.000				

Block 0: Beginning Block

Classification Table[a,b]

Observed			Predicted Depression_ny Nej	Ja	Percentage Correct
Step 0	Depression_ny	Nej	3357	0	100.0
		Ja	269	0	.0
	Overall Percentage				92.6

a. Constant is included in the model.

b. The cut value is ,500

Variables in the Equation

		B	S.E.	Wald	df	Sig.	Exp(B)
Step 0	Constant	-2.524	.063	1586.668	1	.000	.080

Variables not in the Equation

			Score	df	Sig.
Step 0	Variables	Cluster_model_men	33.608	3	<.001
		Cluster_model_men(1)	.253	1	.615
		Cluster_model_men(2)	10.559	1	.001
		Cluster_model_men(3)	14.991	1	<.001
		fodelselandmammapappa_ny(1)	3.685	1	.055
		utbildningmammapappa_ny(1)	.005	1	.942
		Birthyear_decades	7.856	5	.164
		Birthyear_decades(1)	.251	1	.617
		Birthyear_decades(2)	.331	1	.565
		Birthyear_decades(3)	.184	1	.668
		Birthyear_decades(4)	6.416	1	.011
		Birthyear_decades(5)	2.610	1	.106
	Overall Statistics		45.870	10	<.001

Block 1: Method = Enter

Omnibus Tests of Model Coefficients

		Chi-square	df	Sig.
Step 1	Step	42.633	10	<.001
	Block	42.633	10	<.001
	Model	42.633	10	<.001

Model Summary

Step	-2 Log likelihood	Cox & Snell R Square	Nagelkerke R Square
1	1874.330[a]	.012	.028

a. Estimation terminated at iteration number 6 because parameter estimates changed by less than ,001.

Classification Table[a]

Observed	Predicted

			Depression_ny		Percentage
			Nej	Ja	Correct
Step 1	Depression_ny	Nej	3357	0	100.0
		Ja	269	0	.0
	Overall Percentage				92.6

a. The cut value is ,500

Variables in the Equation

		B	S.E.	Wald	df	Sig.	Exp(B)	95% C.I.for EXP(B)	
								Lower	Upper
Step 1[a]	Cluster_model_men			32.474	3	<.001			
	Cluster_model_men(1)	.414	.151	7.565	1	.006	1.513	1.126	2.032
	Cluster_model_men(2)	.906	.214	17.988	1	<.001	2.474	1.628	3.760
	Cluster_model_men(3)	1.133	.234	23.364	1	<.001	3.106	1.962	4.918
	fodelselandmammapappa_ny(1)	.428	.230	3.464	1	.063	1.534	.978	2.408
	utbildningmammapappa_ny(1)	.157	.148	1.118	1	.290	1.170	.875	1.564
	Birthyear_decades			8.569	5	.128			
	Birthyear_decades(1)	-.056	.240	.054	1	.817	.946	.591	1.514
	Birthyear_decades(2)	-.049	.238	.042	1	.838	.952	.598	1.518
	Birthyear_decades(3)	-.041	.243	.028	1	.866	.960	.596	1.546
	Birthyear_decades(4)	-.392	.255	2.349	1	.125	.676	.410	1.115
	Birthyear_decades(5)	.276	.275	1.007	1	.316	1.318	.769	2.259
	Constant	-2.925	.210	193.980	1	<.001	.054		

a. Variable(s) entered on step 1: Cluster_model_men, fodelselandmammapappa_ny, utbildningmammapappa_ny, Birthyear_decades.

Logistic Regression

Notes

Output Created		26-AUG-2025 11:44:26
Comments		
Input	Data	/Users/stevenlc/Library/CloudStorage/OneDrive-Privat/ICloud filer/Doktorander/Rickard/Artikel 4/Menfour class model.sav
	Active Dataset	DataSet3
	File Label	Scored Data File

	Filter	<none>
	Weight	<none>
	Split File	<none>
	N of Rows in Working Data File	4656
Missing Value Handling	Definition of Missing	User-defined missing values are treated as missing
Syntax		LOGISTIC REGRESSION VARIABLES HAD_probable_ångest_ny /METHOD=ENTER Cluster_model_men fodelselandmammapappa_ny utbildningmammapappa_ny Birthyear_decades /CONTRAST (Cluster_model_men)=Indicator(1) /CONTRAST (fodelselandmammapappa_ny)=Indicator(1) /CONTRAST (utbildningmammapappa_ny)=Indicator(1) /CONTRAST (Birthyear_decades)=Indicator(1) /PRINT=CI(95) /CRITERIA=PIN(0.05) POUT(0.10) ITERATE(20) CUT(0.5).
Resources	Processor Time	00:00:00,22
	Elapsed Time	00:00:01,00

Case Processing Summary

Unweighted Cases[a]		N	Percent
Selected Cases	Included in Analysis	3620	77.7
	Missing Cases	1036	22.3
	Total	4656	100.0
Unselected Cases		0	.0
Total		4656	100.0

a. If weight is in effect, see classification table for the total number of cases.

Dependent Variable Encoding

Original Value	Internal Value
Nej	0
Ja	1

Categorical Variables Codings

		Frequency	Parameter coding (1)	(2)	(3)	(4)	(5)
Birthyear_decades	18-27	438	.000	.000	.000	.000	.000
	28-37	580	1.000	.000	.000	.000	.000
	38-47	660	.000	1.000	.000	.000	.000
	48-57	680	.000	.000	1.000	.000	.000
	58-67	875	.000	.000	.000	1.000	.000
	68-74	387	.000	.000	.000	.000	1.000
Cluster_model_men	1.00	1545	.000	.000	.000		
	2.00	1581	1.000	.000	.000		
	3.00	294	.000	1.000	.000		
	4.00	200	.000	.000	1.000		
utbildningmammapappa_ny	Minst en förälder högre utb än grundskola	1803	.000				
	Båda föräldrarna grundskola	1817	1.000				
fodelselandmammapappa_ny	Minst en förälder född i norden	3394	.000				
	Båda födda utanför Norden	226	1.000				

Block 0: Beginning Block

Classification Table[a,b]

Observed			Predicted Anxiety_ny Nej	Ja	Percentage Correct
Step 0	Anxiety_ny	Nej	3518	0	100.0
		Ja	102	0	.0
	Overall Percentage				97.2

a. Constant is included in the model.

b. The cut value is ,500

Variables in the Equation

		B	S.E.	Wald	df	Sig.	Exp(B)
Step 0	Constant	-3.541	.100	1242.681	1	<.001	.029

Variables not in the Equation

			Score	df	Sig.
Step 0	Variables	Cluster_model_men	65.404	3	<.001
		Cluster_model_men(1)	1.262	1	.261
		Cluster_model_men(2)	18.558	1	<.001
		Cluster_model_men(3)	39.881	1	<.001
		fodelselandmammapappa_ny(1)	3.698	1	.054
		utbildningmammapappa_ny(1)	6.003	1	.014
		Birthyear_decades	30.375	5	<.001
		Birthyear_decades(1)	4.396	1	.036
		Birthyear_decades(2)	.784	1	.376
		Birthyear_decades(3)	.975	1	.323
		Birthyear_decades(4)	15.267	1	<.001
		Birthyear_decades(5)	6.602	1	.010
	Overall Statistics		92.677	10	<.001

Block 1: Method = Enter

Omnibus Tests of Model Coefficients

		Chi-square	df	Sig.
Step 1	Step	79.356	10	<.001
	Block	79.356	10	<.001
	Model	79.356	10	<.001

Model Summary

Step	-2 Log likelihood	Cox & Snell R Square	Nagelkerke R Square
1	849.871[a]	.022	.096

a. Estimation terminated at iteration number 8 because parameter estimates changed by less than ,001.

Classification Table[a]

Observed			Predicted: Anxiety_ny Nej	Predicted: Anxiety_ny Ja	Percentage Correct
Step 1	Anxiety_ny	Nej	3518	0	100.0
		Ja	102	0	.0
	Overall Percentage				97.2

a. The cut value is ,500

Variables in the Equation

		B	S.E.	Wald	df	Sig.	Exp(B)	95% C.I.for EXP(B) Lower	95% C.I.for EXP(B) Upper
Step 1[a]	Cluster_model_men			48.347	3	<.001			
	Cluster_model_men(1)	.367	.270	1.852	1	.174	1.443	.851	2.448
	Cluster_model_men(2)	1.449	.318	20.762	1	<.001	4.257	2.283	7.939
	Cluster_model_men(3)	1.881	.323	33.867	1	<.001	6.561	3.482	12.362
	fodelselandmammapappa_ny(1)	.433	.337	1.647	1	.199	1.542	.796	2.986
	utbildningmammapappa_ny(1)	.143	.237	.365	1	.546	1.154	.725	1.837
	Birthyear_decades			21.523	5	<.001			
	Birthyear_decades(1)	-.403	.309	1.699	1	.192	.668	.364	1.225
	Birthyear_decades(2)	-.657	.324	4.108	1	.043	.518	.274	.979
	Birthyear_decades(3)	-.568	.331	2.951	1	.086	.567	.296	1.083
	Birthyear_decades(4)	-1.850	.447	17.127	1	<.001	.157	.065	.378
	Birthyear_decades(5)	-1.888	.646	8.556	1	.003	.151	.043	.536
	Constant	-3.465	.291	142.096	1	<.001	.031		

a. Variable(s) entered on step 1: Cluster_model_men, fodelselandmammapappa_ny, utbildningmammapappa_ny, Birthyear_decades.

Logistic Regression

Notes

Output Created		26-AUG-2025 11:44:27
Comments		
Input	Data	/Users/stevenlc/Library/CloudStorage/OneDrive-Privat/ICloud filer/Doktorander/Rickard/Artikel 4/Menfour class model.sav
	Active Dataset	DataSet3

	File Label	Scored Data File
	Filter	<none>
	Weight	<none>
	Split File	<none>
	N of Rows in Working Data File	4656
Missing Value Handling	Definition of Missing	User-defined missing values are treated as missing
Syntax		LOGISTIC REGRESSION VARIABLES PTSD_score_pos_ny /METHOD=ENTER Cluster_model_men fodelselandmammapappa_ny utbildningmammapappa_ny Birthyear_decades /CONTRAST (Cluster_model_men)=Indicator(1) /CONTRAST (fodelselandmammapappa_ny)=Indicator(1) /CONTRAST (utbildningmammapappa_ny)=Indicator(1) /CONTRAST (Birthyear_decades)=Indicator(1) /PRINT=CI(95) /CRITERIA=PIN(0.05) POUT(0.10) ITERATE(20) CUT(0.5).
Resources	Processor Time	00:00:00,22
	Elapsed Time	00:00:00,00

Case Processing Summary

Unweighted Cases[a]		N	Percent
Selected Cases	Included in Analysis	3582	76.9
	Missing Cases	1074	23.1
	Total	4656	100.0
Unselected Cases		0	.0
Total		4656	100.0

a. If weight is in effect, see classification table for the total number of cases.

Dependent Variable Encoding

Original Value	Internal Value
Nej	0
Ja	1

Categorical Variables Codings

		Frequency	Parameter coding (1)	(2)	(3)	(4)	(5)
Birthyear_decades	18-27	441	.000	.000	.000	.000	.000
	28-37	577	1.000	.000	.000	.000	.000
	38-47	661	.000	1.000	.000	.000	.000
	48-57	679	.000	.000	1.000	.000	.000
	58-67	851	.000	.000	.000	1.000	.000
	68-74	373	.000	.000	.000	.000	1.000
Cluster_model_men	1.00	1541	.000	.000	.000		
	2.00	1557	1.000	.000	.000		
	3.00	284	.000	1.000	.000		
	4.00	200	.000	.000	1.000		
utbildningmammapappa_ny	Minst en förälder högre utb än grundskola	1793	.000				
	Båda föräldrarna grundskola	1789	1.000				
fodelselandmammapappa_ny	Minst en förälder född i norden	3362	.000				
	Båda födda utanför Norden	220	1.000				

Block 0: Beginning Block

Classification Table[a,b]

Observed			Predicted PTSS_ny Nej	Ja	Percentage Correct
Step 0	PTSS_ny	Nej	3424	0	100.0
		Ja	158	0	.0
	Overall Percentage				95.6

a. Constant is included in the model.

b. The cut value is ,500

Variables in the Equation

		B	S.E.	Wald	df	Sig.	Exp(B)
Step 0	Constant	-3.076	.081	1428.991	1	.000	.046

Variables not in the Equation

			Score	df	Sig.
Step 0	Variables	Cluster_model_men	112.800	3	<.001
		Cluster_model_men(1)	1.589	1	.208
		Cluster_model_men(2)	21.715	1	<.001
		Cluster_model_men(3)	79.621	1	<.001
		fodelselandmammapappa_ny(1)	14.656	1	<.001
		utbildningmammapappa_ny(1)	3.758	1	.053
		Birthyear_decades	30.354	5	<.001
		Birthyear_decades(1)	4.468	1	.035
		Birthyear_decades(2)	.205	1	.651
		Birthyear_decades(3)	.401	1	.527
		Birthyear_decades(4)	11.242	1	<.001
		Birthyear_decades(5)	5.071	1	.024
	Overall Statistics		153.062	10	<.001

Block 1: Method = Enter

Omnibus Tests of Model Coefficients

		Chi-square	df	Sig.
Step 1	Step	118.962	10	<.001
	Block	118.962	10	<.001
	Model	118.962	10	<.001

Model Summary

Step	-2 Log likelihood	Cox & Snell R Square	Nagelkerke R Square
1	1176.225[a]	.033	.108

a. Estimation terminated at iteration number 7 because parameter estimates changed by less than ,001.

Classification Table[a]

Observed			Predicted PTSS_ny Nej	Predicted PTSS_ny Ja	Percentage Correct
Step 1	PTSS_ny	Nej	3424	0	100.0
		Ja	158	0	.0
	Overall Percentage				95.6

a. The cut value is ,500

Variables in the Equation

		B	S.E.	Wald	df	Sig.	Exp(B)	95% C.I.for EXP(B) Lower	95% C.I.for EXP(B) Upper
Step 1[a]	Cluster_model_men			89.671	3	<.001			
	Cluster_model_men(1)	.498	.219	5.153	1	.023	1.645	1.070	2.529
	Cluster_model_men(2)	1.481	.268	30.515	1	<.001	4.397	2.600	7.437
	Cluster_model_men(3)	2.223	.262	71.805	1	<.001	9.237	5.524	15.448
	fodelselandmammapappa_ny(1)	.805	.257	9.782	1	.002	2.236	1.350	3.702
	utbildningmammapappa_ny(1)	.284	.195	2.110	1	.146	1.328	.906	1.948
	Birthyear_decades			26.914	5	<.001			
	Birthyear_decades(1)	-.566	.258	4.825	1	.028	.568	.343	.941
	Birthyear_decades(2)	-1.021	.281	13.200	1	<.001	.360	.208	.625
	Birthyear_decades(3)	-.755	.276	7.480	1	.006	.470	.274	.807
	Birthyear_decades(4)	-1.484	.319	21.682	1	<.001	.227	.121	.423
	Birthyear_decades(5)	-1.438	.429	11.237	1	<.001	.237	.102	.550
	Constant	-3.113	.239	169.654	1	<.001	.044		

a. Variable(s) entered on step 1: Cluster_model_men, fodelselandmammapappa_ny, utbildningmammapappa_ny, Birthyear_decades.

Logistic Regression

Notes

Output Created	26-AUG-2025 11:44:27
Comments	

Input	Data	/Users/stevenlc/Library/CloudStorage/OneDrive-Privat/ICloud filer/Doktorander/Rickard/Artikel 4/Menfour class model.sav
	Active Dataset	DataSet3
	File Label	Scored Data File
	Filter	<none>
	Weight	<none>
	Split File	<none>
	N of Rows in Working Data File	4656
Missing Value Handling	Definition of Missing	User-defined missing values are treated as missing
Syntax		LOGISTIC REGRESSION VARIABLES Any_selfharm_ny /METHOD=ENTER Cluster_model_men fodelselandmammapappa_ny utbildningmammapappa_ny Birthyear_decades /CONTRAST (Cluster_model_men)=Indicator(1) /CONTRAST (fodelselandmammapappa_ny)=Indicator(1) /CONTRAST (utbildningmammapappa_ny)=Indicator(1) /CONTRAST (Birthyear_decades)=Indicator(1) /PRINT=CI(95) /CRITERIA=PIN(0.05) POUT(0.10) ITERATE(20) CUT(0.5).
Resources	Processor Time	00:00:00,21
	Elapsed Time	00:00:00,00

Case Processing Summary

Unweighted Cases[a]	N	Percent

Selected Cases	Included in Analysis	3726	80.0
	Missing Cases	930	20.0
	Total	4656	100.0
Unselected Cases		0	.0
Total		4656	100.0

a. If weight is in effect, see classification table for the total number of cases.

Dependent Variable Encoding

Original Value	Internal Value
Nej	0
Ja	1

Categorical Variables Codings

		Frequency	Parameter coding (1)	(2)	(3)	(4)	(5)
Birthyear_decades	18-27	458	.000	.000	.000	.000	.000
	28-37	591	1.000	.000	.000	.000	.000
	38-47	676	.000	1.000	.000	.000	.000
	48-57	706	.000	.000	1.000	.000	.000
	58-67	890	.000	.000	.000	1.000	.000
	68-74	405	.000	.000	.000	.000	1.000
Cluster_model_men	1.00	1601	.000	.000	.000		
	2.00	1621	1.000	.000	.000		
	3.00	297	.000	1.000	.000		
	4.00	207	.000	.000	1.000		
utbildningmammapappa_ny	Minst en förälder högre utb än grundskola	1858	.000				
	Båda föräldrarna grundskola	1868	1.000				
fodelselandmammapappa_ny	Minst en förälder född i norden	3490	.000				
	Båda födda utanför Norden	236	1.000				

Block 0: Beginning Block

Classification Table[a,b]

Observed	Predicted: Self-harm_ny	Percentage

			Nej	Ja	Correct
Step 0	Self-harm_ny	Nej	3422	0	100.0
		Ja	304	0	.0
	Overall Percentage				91.8

a. Constant is included in the model.

b. The cut value is ,500

Variables in the Equation

		B	S.E.	Wald	df	Sig.	Exp(B)
Step 0	Constant	-2.421	.060	1636.377	1	.000	.089

Variables not in the Equation

			Score	df	Sig.
Step 0	Variables	Cluster_model_men	260.595	3	<.001
		Cluster_model_men(1)	2.367	1	.124
		Cluster_model_men(2)	66.008	1	<.001
		Cluster_model_men(3)	132.825	1	<.001
		fodelselandmammapappa_ny(1)	1.667	1	.197
		utbildningmammapappa_ny(1)	36.404	1	<.001
		Birthyear_decades	91.629	5	<.001
		Birthyear_decades(1)	30.626	1	<.001
		Birthyear_decades(2)	.195	1	.659
		Birthyear_decades(3)	2.150	1	.143
		Birthyear_decades(4)	20.955	1	<.001
		Birthyear_decades(5)	23.187	1	<.001
	Overall Statistics		332.655	10	<.001

Block 1: Method = Enter

Omnibus Tests of Model Coefficients

		Chi-square	df	Sig.
Step 1	Step	302.255	10	<.001
	Block	302.255	10	<.001
	Model	302.255	10	<.001

Model Summary

Step	-2 Log likelihood	Cox & Snell R Square	Nagelkerke R Square
1	1803.925[a]	.078	.180

a. Estimation terminated at iteration number 7 because parameter estimates changed by less than ,001.

Classification Table[a]

Observed			Predicted		
			Self-harm_ny		Percentage Correct
			Nej	Ja	
Step 1	Self-harm_ny	Nej	3413	9	99.7
		Ja	299	5	1.6
	Overall Percentage				91.7

a. The cut value is ,500

Variables in the Equation

		B	S.E.	Wald	df	Sig.	Exp(B)	95% C.I.for EXP(B) Lower	Upper
Step 1[a]	Cluster_model_men			185.256	3	<.001			
	Cluster_model_men(1)	1.246	.191	42.733	1	<.001	3.476	2.392	5.050
	Cluster_model_men(2)	2.348	.224	109.439	1	<.001	10.460	6.738	16.238
	Cluster_model_men(3)	2.802	.232	145.936	1	<.001	16.482	10.461	25.969
	fodelselandmammapappa_ny(1)	-.624	.296	4.435	1	.035	.536	.300	.958
	utbildningmammapappa_ny(1)	-.071	.152	.216	1	.642	.932	.692	1.255
	Birthyear_decades			50.848	5	<.001			
	Birthyear_decades(1)	-.322	.188	2.936	1	.087	.725	.502	1.047
	Birthyear_decades(2)	-.914	.207	19.518	1	<.001	.401	.267	.602
	Birthyear_decades(3)	-1.007	.220	20.884	1	<.001	.365	.237	.563
	Birthyear_decades(4)	-1.304	.238	29.919	1	<.001	.271	.170	.433
	Birthyear_decades(5)	-1.999	.403	24.613	1	<.001	.136	.062	.298
	Constant	-2.792	.206	184.349	1	<.001	.061		

a. Variable(s) entered on step 1: Cluster_model_men, fodelselandmammapappa_ny, utbildningmammapappa_ny, Birthyear_decades.

Logistic Regression

Notes

Output Created		26-AUG-2025 11:44:27
Comments		
Input	Data	/Users/stevenlc/Library/CloudStorage/OneDrive-Privat/ICloud filer/Doktorander/Rickard/Artikel 4/Menfour class model.sav
	Active Dataset	DataSet3
	File Label	Scored Data File
	Filter	<none>
	Weight	<none>
	Split File	<none>
	N of Rows in Working Data File	4656
Missing Value Handling	Definition of Missing	User-defined missing values are treated as missing
Syntax		LOGISTIC REGRESSION VARIABLES Symtom_score_måttlig_ny<br>/METHOD=ENTER Cluster_model_men fodelselandmammapappa_ny utbildningmammapappa_ny Birthyear_decades<br>/CONTRAST (Cluster_model_men)=Indicator(1)<br>/CONTRAST (fodelselandmammapappa_ny)=Indicator(1)<br>/CONTRAST (utbildningmammapappa_ny)=Indicator(1)<br>/CONTRAST (Birthyear_decades)=Indicator(1)<br>/PRINT=CI(95)<br>/CRITERIA=PIN(0.05) POUT(0.10) ITERATE(20) CUT(0.5).
Resources	Processor Time	00:00:00,22
	Elapsed Time	00:00:00,00

Case Processing Summary

Unweighted Cases[a]		N	Percent
Selected Cases	Included in Analysis	3466	74.4
	Missing Cases	1190	25.6
	Total	4656	100.0
Unselected Cases		0	.0
Total		4656	100.0

a. If weight is in effect, see classification table for the total number of cases.

Dependent Variable Encoding

Original Value	Internal Value
Nej	0
Ja	1

Categorical Variables Codings

		Frequency	Parameter coding (1)	(2)	(3)	(4)	(5)
Birthyear_decades	18-27	439	.000	.000	.000	.000	.000
	28-37	573	1.000	.000	.000	.000	.000
	38-47	645	.000	1.000	.000	.000	.000
	48-57	666	.000	.000	1.000	.000	.000
	58-67	798	.000	.000	.000	1.000	.000
	68-74	345	.000	.000	.000	.000	1.000
Cluster_model_men	1.00	1472	.000	.000	.000		
	2.00	1527	1.000	.000	.000		
	3.00	276	.000	1.000	.000		
	4.00	191	.000	.000	1.000		
utbildningmammapappa_ny	Minst en förälder högre utb än grundskola	1774	.000				
	Båda föräldrarna grundskola	1692	1.000				
fodelselandmammapappa_ny	Minst en förälder född i norden	3250	.000				
	Båda födda utanför Norden	216	1.000				

Block 0: Beginning Block

Classification Table[a,b]

			Predicted		
			Somatization_ny		Percentage Correct
	Observed		Nej	Ja	
Step 0	Somatization_ny	Nej	3346	0	100.0
		Ja	120	0	.0
	Overall Percentage				96.5

a. Constant is included in the model.

b. The cut value is ,500

Variables in the Equation

		B	S.E.	Wald	df	Sig.	Exp(B)
Step 0	Constant	-3.328	.093	1283.077	1	<.001	.036

Variables not in the Equation

			Score	df	Sig.
Step 0	Variables	Cluster_model_men	74.193	3	<.001
		Cluster_model_men(1)	7.742	1	.005
		Cluster_model_men(2)	24.574	1	<.001
		Cluster_model_men(3)	44.519	1	<.001
		fodelselandmammapappa_ny(1)	4.504	1	.034
		utbildningmammapappa_ny(1)	.404	1	.525
		Birthyear_decades	12.014	5	.035
		Birthyear_decades(1)	2.132	1	.144
		Birthyear_decades(2)	.310	1	.578
		Birthyear_decades(3)	.864	1	.353
		Birthyear_decades(4)	1.977	1	.160
		Birthyear_decades(5)	3.531	1	.060
	Overall Statistics		94.539	10	<.001

Block 1: Method = Enter

Omnibus Tests of Model Coefficients

		Chi-square	df	Sig.
Step 1	Step	73.302	10	<.001
	Block	73.302	10	<.001
	Model	73.302	10	<.001

Model Summary

Step	-2 Log likelihood	Cox & Snell R Square	Nagelkerke R Square
1	969.678[a]	.021	.081

a. Estimation terminated at iteration number 7 because parameter estimates changed by less than ,001.

Classification Table[a]

	Observed		Predicted: Somatization_ny Nej	Predicted: Somatization_ny Ja	Predicted: Percentage Correct
Step 1	Somatization_ny	Nej	3346	0	100.0
		Ja	120	0	.0
	Overall Percentage				96.5

a. The cut value is ,500

Variables in the Equation

		B	S.E.	Wald	df	Sig.	Exp(B)	95% C.I.for EXP(B) Lower	95% C.I.for EXP(B) Upper
Step 1[a]	Cluster_model_men			63.265	3	<.001			
	Cluster_model_men(1)	.195	.242	.651	1	.420	1.215	.757	1.952
	Cluster_model_men(2)	1.451	.280	26.914	1	<.001	4.268	2.467	7.384
	Cluster_model_men(3)	1.879	.290	41.970	1	<.001	6.549	3.709	11.565
	fodelselandmammapappa_ny(1)	.634	.314	4.069	1	.044	1.885	1.018	3.490
	utbildningmammapappa_ny(1)	-.099	.212	.218	1	.640	.906	.597	1.373
	Birthyear_decades			15.312	5	.009			
	Birthyear_decades(1)	.265	.472	.315	1	.574	1.303	.517	3.288
	Birthyear_decades(2)	.546	.453	1.458	1	.227	1.727	.711	4.193
	Birthyear_decades(3)	.943	.442	4.550	1	.033	2.569	1.080	6.112
	Birthyear_decades(4)	1.103	.441	6.246	1	.012	3.014	1.269	7.160
	Birthyear_decades(5)	1.434	.479	8.966	1	.003	4.196	1.641	10.728
	Constant	-4.597	.417	121.600	1	<.001	.010		

a. Variable(s) entered on step 1: Cluster_model_men, fodelselandmammapappa_ny, utbildningmammapappa_ny, Birthyear_decades.

Logistic Regression

Notes

Output Created		26-AUG-2025 11:44:27
Comments		
Input	Data	/Users/stevenlc/Library/CloudStorage/OneDrive-Privat/ICloud filer/Doktorander/Rickard/Artikel 4/Menfour class model.sav
	Active Dataset	DataSet3
	File Label	Scored Data File
	Filter	<none>
	Weight	<none>
	Split File	<none>
	N of Rows in Working Data File	4656
Missing Value Handling	Definition of Missing	User-defined missing values are treated as missing
Syntax		LOGISTIC REGRESSION VARIABLES IBS_ny<br>/METHOD=ENTER Cluster_model_men fodelselandmammapappa_ny utbildningmammapappa_ny Birthyear_decades<br>/CONTRAST (Cluster_model_men)=Indicator(1)<br>/CONTRAST (fodelselandmammapappa_ny)=Indicator(1)<br>/CONTRAST (utbildningmammapappa_ny)=Indicator(1)<br>/CONTRAST (Birthyear_decades)=Indicator(1)<br>/PRINT=CI(95)<br>/CRITERIA=PIN(0.05) POUT(0.10) ITERATE(20) CUT(0.5).
Resources	Processor Time	00:00:00,22
	Elapsed Time	00:00:01,00

Case Processing Summary

Unweighted Cases[a]		N	Percent
Selected Cases	Included in Analysis	3741	80.3
	Missing Cases	915	19.7
	Total	4656	100.0
Unselected Cases		0	.0
Total		4656	100.0

a. If weight is in effect, see classification table for the total number of cases.

Dependent Variable Encoding

Original Value	Internal Value
Nej	0
Ja	1

Categorical Variables Codings

			Parameter coding				
		Frequency	(1)	(2)	(3)	(4)	(5)
Birthyear_decades	18-27	458	.000	.000	.000	.000	.000
	28-37	595	1.000	.000	.000	.000	.000
	38-47	678	.000	1.000	.000	.000	.000
	48-57	709	.000	.000	1.000	.000	.000
	58-67	895	.000	.000	.000	1.000	.000
	68-74	406	.000	.000	.000	.000	1.000
Cluster_model_men	1.00	1606	.000	.000	.000		
	2.00	1627	1.000	.000	.000		
	3.00	300	.000	1.000	.000		
	4.00	208	.000	.000	1.000		
utbildningmammapappa_ny	Minst en förälder högre utb än grundskola	1866	.000				
	Båda föräldrarna grundskola	1875	1.000				
fodelselandmammapappa_ny	Minst en förälder född i norden	3504	.000				
	Båda födda utanför Norden	237	1.000				

Block 0: Beginning Block

Classification Table[a,b]

Observed			Predicted IBS_ny Nej	Ja	Percentage Correct
Step 0	IBS_ny	Nej	3641	0	100.0
		Ja	100	0	.0
	Overall Percentage				97.3

a. Constant is included in the model.

b. The cut value is ,500

Variables in the Equation

		B	S.E.	Wald	df	Sig.	Exp(B)
Step 0	Constant	-3.595	.101	1257.746	1	<.001	.027

Variables not in the Equation

			Score	df	Sig.
Step 0	Variables	Cluster_model_men	16.440	3	<.001
		Cluster_model_men(1)	.850	1	.357
		Cluster_model_men(2)	6.788	1	.009
		Cluster_model_men(3)	3.857	1	.050
		fodelselandmammapappa_ny(1)	.309	1	.578
		utbildningmammapappa_ny (1)	.001	1	.981
		Birthyear_decades	14.168	5	.015
		Birthyear_decades(1)	2.678	1	.102
		Birthyear_decades(2)	.244	1	.621
		Birthyear_decades(3)	1.640	1	.200
		Birthyear_decades(4)	4.650	1	.031
		Birthyear_decades(5)	4.013	1	.045
	Overall Statistics		37.729	10	<.001

Block 1: Method = Enter

Omnibus Tests of Model Coefficients

		Chi-square	df	Sig.
Step 1	Step	37.165	10	<.001
	Block	37.165	10	<.001
	Model	37.165	10	<.001

Model Summary

Step	-2 Log likelihood	Cox & Snell R Square	Nagelkerke R Square
1	884.525[a]	.010	.045

a. Estimation terminated at iteration number 7 because parameter estimates changed by less than ,001.

Classification Table[a]

Observed			Predicted IBS_ny Nej	Predicted IBS_ny Ja	Percentage Correct
Step 1	IBS_ny	Nej	3641	0	100.0
		Ja	100	0	.0
	Overall Percentage				97.3

a. The cut value is ,500

Variables in the Equation

		B	S.E.	Wald	df	Sig.	Exp(B)	95% C.I.for EXP(B) Lower	95% C.I.for EXP(B) Upper
Step 1[a]	Cluster_model_men			18.584	3	<.001			
	Cluster_model_men(1)	.718	.249	8.335	1	.004	2.049	1.259	3.336
	Cluster_model_men(2)	1.258	.334	14.191	1	<.001	3.519	1.829	6.770
	Cluster_model_men(3)	1.201	.386	9.688	1	.002	3.323	1.560	7.078
	fodelselandmammapappa_ny(1)	-.245	.470	.272	1	.602	.782	.311	1.967
	utbildningmammapappa_ny(1)	-.359	.231	2.406	1	.121	.699	.444	1.099
	Birthyear_decades			20.388	5	.001			
	Birthyear_decades(1)	.170	.522	.106	1	.745	1.185	.426	3.297
	Birthyear_decades(2)	.840	.475	3.121	1	.077	2.316	.912	5.879
	Birthyear_decades(3)	.556	.503	1.222	1	.269	1.743	.651	4.670
	Birthyear_decades(4)	1.354	.471	8.254	1	.004	3.872	1.538	9.749
	Birthyear_decades(5)	1.590	.507	9.833	1	.002	4.904	1.815	13.248
	Constant	-4.845	.451	115.269	1	<.001	.008		

a. Variable(s) entered on step 1: Cluster_model_men, fodelselandmammapappa_ny, utbildningmammapappa_ny, Birthyear_decades.

Logistic Regression

Notes

Output Created		26-AUG-2025 11:44:28
Comments		
Input	Data	/Users/stevenlc/Library/CloudStorage/OneDrive-Privat/ICloud filer/Doktorander/Rickard/Artikel 4/Menfour class model.sav
	Active Dataset	DataSet3
	File Label	Scored Data File
	Filter	<none>
	Weight	<none>
	Split File	<none>
	N of Rows in Working Data File	4656
Missing Value Handling	Definition of Missing	User-defined missing values are treated as missing
Syntax		LOGISTIC REGRESSION VARIABLES Fibromyalgi_ny<br>/METHOD=ENTER Cluster_model_men fodelselandmammapappa_ny utbildningmammapappa_ny Birthyear_decades<br>/CONTRAST (Cluster_model_men)=Indicator(1)<br>/CONTRAST (fodelselandmammapappa_ny)=Indicator(1)<br>/CONTRAST (utbildningmammapappa_ny)=Indicator(1)<br>/CONTRAST (Birthyear_decades)=Indicator(1)<br>/PRINT=CI(95)<br>/CRITERIA=PIN(0.05) POUT(0.10) ITERATE(20) CUT(0.5).
Resources	Processor Time	00:00:00,22
	Elapsed Time	00:00:00,00

Case Processing Summary

Unweighted Cases[a]		N	Percent
Selected Cases	Included in Analysis	3741	80.3
	Missing Cases	915	19.7
	Total	4656	100.0
Unselected Cases		0	.0
Total		4656	100.0

a. If weight is in effect, see classification table for the total number of cases.

Dependent Variable Encoding

Original Value	Internal Value
Nej	0
Ja	1

Categorical Variables Codings

			Parameter coding				
		Frequency	(1)	(2)	(3)	(4)	(5)
Birthyear_decades	18-27	458	.000	.000	.000	.000	.000
	28-37	595	1.000	.000	.000	.000	.000
	38-47	678	.000	1.000	.000	.000	.000
	48-57	709	.000	.000	1.000	.000	.000
	58-67	895	.000	.000	.000	1.000	.000
	68-74	406	.000	.000	.000	.000	1.000
Cluster_model_men	1.00	1606	.000	.000	.000		
	2.00	1627	1.000	.000	.000		
	3.00	300	.000	1.000	.000		
	4.00	208	.000	.000	1.000		
utbildningmammapappa_ny	Minst en förälder högre utb än grundskola	1866	.000				
	Båda föräldrarna grundskola	1875	1.000				
fodelselandmammapappa_ny	Minst en förälder född i norden	3504	.000				
	Båda födda utanför Norden	237	1.000				

Block 0: Beginning Block

Classification Table[a,b]

Observed			Predicted Fibromyalgia_ny Nej	Ja	Percentage Correct
Step 0	Fibromyalgia_ny	Nej	3718	0	100.0
		Ja	23	0	.0
	Overall Percentage				99.4

a. Constant is included in the model.

b. The cut value is ,500

Variables in the Equation

		B	S.E.	Wald	df	Sig.	Exp(B)
Step 0	Constant	-5.085	.209	591.164	1	<.001	.006

Variables not in the Equation

			Score	df	Sig.
Step 0	Variables	Cluster_model_men	3.253	3	.354
		Cluster_model_men(1)	.714	1	.398
		Cluster_model_men(2)	.423	1	.515
		Cluster_model_men(3)	2.468	1	.116
		fodelselandmammapappa_ny(1)	.217	1	.641
		utbildningmammapappa_ny(1)	2.110	1	.146
		Birthyear_decades	10.181	5	.070
		Birthyear_decades(1)	2.311	1	.128
		Birthyear_decades(2)	1.386	1	.239
		Birthyear_decades(3)	.526	1	.468
		Birthyear_decades(4)	7.264	1	.007
		Birthyear_decades(5)	1.023	1	.312
	Overall Statistics		13.963	10	.175

Block 1: Method = Enter

Omnibus Tests of Model Coefficients

		Chi-square	df	Sig.
Step 1	Step	13.221	10	.212
	Block	13.221	10	.212
	Model	13.221	10	.212

Model Summary

Step	-2 Log likelihood	Cox & Snell R Square	Nagelkerke R Square
1	266.852[a]	.004	.049

a. Estimation terminated at iteration number 9 because parameter estimates changed by less than ,001.

Classification Table[a]

Observed			Predicted: Fibromyalgia_ny Nej	Ja	Percentage Correct
Step 1	Fibromyalgia_ny	Nej	3718	0	100.0
		Ja	23	0	.0
	Overall Percentage				99.4

a. The cut value is ,500

Variables in the Equation

		B	S.E.	Wald	df	Sig.	Exp(B)	95% C.I.for EXP(B) Lower	Upper
Step 1[a]	Cluster_model_men			3.467	3	.325			
	Cluster_model_men(1)	-.071	.473	.023	1	.880	.931	.368	2.355
	Cluster_model_men(2)	-.510	1.051	.235	1	.628	.600	.076	4.715
	Cluster_model_men(3)	1.079	.670	2.593	1	.107	2.941	.791	10.932
	fodelselandmammapappa_ny(1)	.560	.755	.551	1	.458	1.751	.399	7.687
	utbildningmammapappa_ny(1)	.168	.504	.111	1	.740	1.183	.440	3.176
	Birthyear_decades			7.152	5	.210			
	Birthyear_decades(1)	-1.014	1.230	.680	1	.410	.363	.033	4.042
	Birthyear_decades(2)	-.507	1.020	.247	1	.619	.602	.082	4.450
	Birthyear_decades(3)	-.133	.953	.020	1	.889	.875	.135	5.664
	Birthyear_decades(4)	.943	.840	1.262	1	.261	2.569	.495	13.321
	Birthyear_decades(5)	.741	.940	.621	1	.431	2.098	.332	13.251
	Constant	-5.522	.767	51.837	1	<.001	.004		

a. Variable(s) entered on step 1: Cluster_model_men, fodelselandmammapappa_ny, utbildningmammapappa_ny, Birthyear_decades.

Logistic Regression

Notes

Output Created		26-AUG-2025 11:44:28
Comments		
Input	Data	/Users/stevenlc/Library/CloudStorage/OneDrive-Privat/ICloud filer/Doktorander/Rickard/Artikel 4/Menfour class model.sav
	Active Dataset	DataSet3
	File Label	Scored Data File
	Filter	<none>
	Weight	<none>
	Split File	<none>
	N of Rows in Working Data File	4656
Missing Value Handling	Definition of Missing	User-defined missing values are treated as missing
Syntax		LOGISTIC REGRESSION VARIABLES Ischemic_HD_ny /METHOD=ENTER Cluster_model_men fodelselandmammapappa_ny utbildningmammapappa_ny Birthyear_decades /CONTRAST (Cluster_model_men)=Indicator(1) /CONTRAST (fodelselandmammapappa_ny)=Indicator(1) /CONTRAST (utbildningmammapappa_ny)=Indicator(1) /CONTRAST (Birthyear_decades)=Indicator(1) /PRINT=CI(95) /CRITERIA=PIN(0.05) POUT(0.10) ITERATE(20) CUT(0.5).
Resources	Processor Time	00:00:00,22
	Elapsed Time	00:00:00,00

Case Processing Summary

Unweighted Cases[a]		N	Percent
Selected Cases	Included in Analysis	3741	80.3
	Missing Cases	915	19.7
	Total	4656	100.0
Unselected Cases		0	.0
Total		4656	100.0

a. If weight is in effect, see classification table for the total number of cases.

Dependent Variable Encoding

Original Value	Internal Value
Nej	0
Ja	1

Categorical Variables Codings

			Parameter coding				
		Frequency	(1)	(2)	(3)	(4)	(5)
Birthyear_decades	18-27	458	.000	.000	.000	.000	.000
	28-37	595	1.000	.000	.000	.000	.000
	38-47	678	.000	1.000	.000	.000	.000
	48-57	709	.000	.000	1.000	.000	.000
	58-67	895	.000	.000	.000	1.000	.000
	68-74	406	.000	.000	.000	.000	1.000
Cluster_model_men	1.00	1606	.000	.000	.000		
	2.00	1627	1.000	.000	.000		
	3.00	300	.000	1.000	.000		
	4.00	208	.000	.000	1.000		
utbildningmammapappa_ny	Minst en förälder högre utb än grundskola	1866	.000				
	Båda föräldrarna grundskola	1875	1.000				
fodelselandmammapappa_ny	Minst en förälder född i norden	3504	.000				
	Båda födda utanför Norden	237	1.000				

Block 0: Beginning Block

Classification Table[a,b]

Observed			Predicted IHD_ny Nej	Predicted IHD_ny Ja	Percentage Correct
Step 0	IHD_ny	Nej	3469	0	100.0
		Ja	272	0	.0
	Overall Percentage				92.7

a. Constant is included in the model.

b. The cut value is ,500

Variables in the Equation

		B	S.E.	Wald	df	Sig.	Exp(B)
Step 0	Constant	-2.546	.063	1634.710	1	.000	.078

Variables not in the Equation

			Score	df	Sig.
Step 0	Variables	Cluster_model_men	15.007	3	.002
		Cluster_model_men(1)	12.019	1	<.001
		Cluster_model_men(2)	.002	1	.965
		Cluster_model_men(3)	.340	1	.560
		fodelselandmammapappa_ny(1)	2.595	1	.107
		utbildningmammapappa_ny(1)	54.595	1	<.001
		Birthyear_decades	316.731	5	<.001
		Birthyear_decades(1)	45.692	1	<.001
		Birthyear_decades(2)	35.199	1	<.001
		Birthyear_decades(3)	8.882	1	.003
		Birthyear_decades(4)	65.720	1	<.001
		Birthyear_decades(5)	186.606	1	<.001
	Overall Statistics		318.214	10	<.001

Block 1: Method = Enter

Omnibus Tests of Model Coefficients

		Chi-square	df	Sig.
Step 1	Step	301.180	10	<.001
	Block	301.180	10	<.001

	Model	301.180	10	<.001

Model Summary

Step	-2 Log likelihood	Cox & Snell R Square	Nagelkerke R Square
1	1648.537[a]	.077	.190

a. Estimation terminated at iteration number 8 because parameter estimates changed by less than ,001.

Classification Table[a]

Observed			Predicted IHD_ny Nej	Ja	Percentage Correct
Step 1	IHD_ny	Nej	3469	0	100.0
		Ja	272	0	.0
	Overall Percentage				92.7

a. The cut value is ,500

Variables in the Equation

		B	S.E.	Wald	df	Sig.	Exp(B)	95% C.I.for EXP(B) Lower	Upper
Step 1[a]	Cluster_model_men			1.602	3	.659			
	Cluster_model_men(1)	-.103	.146	.499	1	.480	.902	.677	1.201
	Cluster_model_men(2)	.184	.251	.539	1	.463	1.203	.735	1.967
	Cluster_model_men(3)	.127	.316	.162	1	.687	1.136	.612	2.108
	fodelselandmammapappa_ny(1)	-.173	.330	.273	1	.601	.841	.440	1.608
	utbildningmammapappa_ny(1)	-.034	.155	.047	1	.827	.967	.713	1.311
	Birthyear_decades			178.066	5	<.001			
	Birthyear_decades(1)	-.490	.674	.528	1	.468	.613	.163	2.297
	Birthyear_decades(2)	.564	.533	1.120	1	.290	1.757	.619	4.991
	Birthyear_decades(3)	1.485	.490	9.175	1	.002	4.414	1.689	11.537
	Birthyear_decades(4)	2.642	.472	31.313	1	<.001	14.038	5.565	35.412
	Birthyear_decades(5)	3.350	.478	49.182	1	<.001	28.494	11.173	72.664
	Constant	-4.458	.458	94.903	1	<.001	.012		

a. Variable(s) entered on step 1: Cluster_model_men, fodelselandmammapappa_ny, utbildningmammapappa_ny, Birthyear_decades.

Logistic Regression

Notes

Output Created		26-AUG-2025 11:44:28
Comments		
Input	Data	/Users/stevenlc/Library/CloudStorage/OneDrive-Privat/ICloud filer/Doktorander/Rickard/Artikel 4/Menfour class model.sav
	Active Dataset	DataSet3
	File Label	Scored Data File
	Filter	<none>
	Weight	<none>
	Split File	<none>
	N of Rows in Working Data File	4656
Missing Value Handling	Definition of Missing	User-defined missing values are treated as missing
Syntax		LOGISTIC REGRESSION VARIABLES KOL_ny<br>/METHOD=ENTER Cluster_model_men fodelselandmammapappa_ny utbildningmammapappa_ny Birthyear_decades<br>/CONTRAST (Cluster_model_men)=Indicator(1)<br>/CONTRAST (fodelselandmammapappa_ny)=Indicator(1)<br>/CONTRAST (utbildningmammapappa_ny)=Indicator(1)<br>/CONTRAST (Birthyear_decades)=Indicator(1)<br>/PRINT=CI(95)<br>/CRITERIA=PIN(0.05) POUT(0.10) ITERATE(20) CUT(0.5).
Resources	Processor Time	00:00:00,22
	Elapsed Time	00:00:00,00

Case Processing Summary

Unweighted Cases[a]		N	Percent
Selected Cases	Included in Analysis	3741	80.3
	Missing Cases	915	19.7
	Total	4656	100.0
Unselected Cases		0	.0
Total		4656	100.0

a. If weight is in effect, see classification table for the total number of cases.

Dependent Variable Encoding

Original Value	Internal Value
Nej	0
Ja	1

Categorical Variables Codings

			Parameter coding				
		Frequency	(1)	(2)	(3)	(4)	(5)
Birthyear_decades	18-27	458	.000	.000	.000	.000	.000
	28-37	595	1.000	.000	.000	.000	.000
	38-47	678	.000	1.000	.000	.000	.000
	48-57	709	.000	.000	1.000	.000	.000
	58-67	895	.000	.000	.000	1.000	.000
	68-74	406	.000	.000	.000	.000	1.000
Cluster_model_men	1.00	1606	.000	.000	.000		
	2.00	1627	1.000	.000	.000		
	3.00	300	.000	1.000	.000		
	4.00	208	.000	.000	1.000		
utbildningmammapappa_ny	Minst en förälder högre utb än grundskola	1866	.000				
	Båda föräldrarna grundskola	1875	1.000				
fodelselandmammapappa_ny	Minst en förälder född i norden	3504	.000				
	Båda födda utanför Norden	237	1.000				

Block 0: Beginning Block

Classification Table[a,b]

Observed			Predicted COPD_ny Nej	Predicted COPD_ny Ja	Percentage Correct
Step 0	COPD_ny	Nej	3685	0	100.0
		Ja	56	0	.0
	Overall Percentage				98.5

a. Constant is included in the model.

b. The cut value is ,500

Variables in the Equation

		B	S.E.	Wald	df	Sig.	Exp(B)
Step 0	Constant	-4.187	.135	966.888	1	<.001	.015

Variables not in the Equation

			Score	df	Sig.
Step 0	Variables	Cluster_model_men	3.052	3	.384
		Cluster_model_men(1)	.409	1	.522
		Cluster_model_men(2)	.064	1	.801
		Cluster_model_men(3)	2.876	1	.090
		fodelselandmammapappa_ny(1)	.092	1	.762
		utbildningmammapappa_ny(1)	10.325	1	.001
		Birthyear_decades	33.604	5	<.001
		Birthyear_decades(1)	8.473	1	.004
		Birthyear_decades(2)	.564	1	.453
		Birthyear_decades(3)	.806	1	.369
		Birthyear_decades(4)	9.184	1	.002
		Birthyear_decades(5)	14.917	1	<.001
	Overall Statistics		39.288	10	<.001

Block 1: Method = Enter

Omnibus Tests of Model Coefficients

		Chi-square	df	Sig.
Step 1	Step	41.060	10	<.001
	Block	41.060	10	<.001

	Model	41.060	10	<.001

Model Summary

Step	-2 Log likelihood	Cox & Snell R Square	Nagelkerke R Square
1	540.695[a]	.011	.076

a. Estimation terminated at iteration number 9 because parameter estimates changed by less than ,001.

Classification Table[a]

	Observed		Predicted COPD_ny Nej	Predicted COPD_ny Ja	Predicted Percentage Correct
Step 1	COPD_ny	Nej	3685	0	100.0
		Ja	56	0	.0
	Overall Percentage				98.5

a. The cut value is ,500

Variables in the Equation

		B	S.E.	Wald	df	Sig.	Exp(B)	95% C.I.for EXP(B) Lower	95% C.I.for EXP(B) Upper
Step 1[a]	Cluster_model_men			5.462	3	.141			
	Cluster_model_men(1)	.279	.306	.829	1	.362	1.321	.725	2.407
	Cluster_model_men(2)	.451	.504	.801	1	.371	1.570	.585	4.212
	Cluster_model_men(3)	1.096	.477	5.273	1	.022	2.991	1.174	7.621
	fodelselandmammapappa_ny(1)	.117	.607	.037	1	.848	1.124	.342	3.692
	utbildningmammapappa_ny(1)	.259	.324	.640	1	.424	1.296	.686	2.448
	Birthyear_decades			20.357	5	.001			
	Birthyear_decades(1)	-.366	1.417	.067	1	.796	.693	.043	11.142
	Birthyear_decades(2)	1.536	1.070	2.060	1	.151	4.644	.570	37.811
	Birthyear_decades(3)	1.497	1.076	1.934	1	.164	4.469	.542	36.854
	Birthyear_decades(4)	2.343	1.047	5.014	1	.025	10.416	1.339	81.006
	Birthyear_decades(5)	2.758	1.062	6.747	1	.009	15.764	1.968	126.284
	Constant	-6.401	1.023	39.168	1	<.001	.002		

a. Variable(s) entered on step 1: Cluster_model_men, fodelselandmammapappa_ny, utbildningmammapappa_ny, Birthyear_decades.

Logistic Regression

Notes

Output Created		26-AUG-2025 11:44:28
Comments		
Input	Data	/Users/stevenlc/Library/CloudStorage/OneDrive-Privat/ICloud filer/Doktorander/Rickard/Artikel 4/Menfour class model.sav
	Active Dataset	DataSet3
	File Label	Scored Data File
	Filter	<none>
	Weight	<none>
	Split File	<none>
	N of Rows in Working Data File	4656
Missing Value Handling	Definition of Missing	User-defined missing values are treated as missing
Syntax		LOGISTIC REGRESSION VARIABLES Diabetes_II_ny /METHOD=ENTER Cluster_model_men fodelselandmammapappa_ny utbildningmammapappa_ny Birthyear_decades /CONTRAST (Cluster_model_men)=Indicator(1) /CONTRAST (fodelselandmammapappa_ny)=Indicator(1) /CONTRAST (utbildningmammapappa_ny)=Indicator(1) /CONTRAST (Birthyear_decades)=Indicator(1) /PRINT=CI(95) /CRITERIA=PIN(0.05) POUT(0.10) ITERATE(20) CUT(0.5).
Resources	Processor Time	00:00:00,23

Elapsed Time	00:00:00,00

Case Processing Summary

Unweighted Cases[a]		N	Percent
Selected Cases	Included in Analysis	3741	80.3
	Missing Cases	915	19.7
	Total	4656	100.0
Unselected Cases		0	.0
Total		4656	100.0

a. If weight is in effect, see classification table for the total number of cases.

Dependent Variable Encoding

Original Value	Internal Value
Nej	0
Ja	1

Categorical Variables Codings

		Frequency	Parameter coding (1)	(2)	(3)	(4)	(5)
Birthyear_decades	18-27	458	.000	.000	.000	.000	.000
	28-37	595	1.000	.000	.000	.000	.000
	38-47	678	.000	1.000	.000	.000	.000
	48-57	709	.000	.000	1.000	.000	.000
	58-67	895	.000	.000	.000	1.000	.000
	68-74	406	.000	.000	.000	.000	1.000
Cluster_model_men	1.00	1606	.000	.000	.000		
	2.00	1627	1.000	.000	.000		
	3.00	300	.000	1.000	.000		
	4.00	208	.000	.000	1.000		
utbildningmammapappa_ny	Minst en förälder högre utb än grundskola	1866	.000				
	Båda föräldrarna grundskola	1875	1.000				
fodelselandmammapappa_ny	Minst en förälder född i norden	3504	.000				
	Båda födda utanför Norden	237	1.000				

Block 0: Beginning Block

Classification Table[a,b]

Observed			Predicted: Diabetes type II_ny Nej	Predicted: Diabetes type II_ny Ja	Predicted: Percentage Correct
Step 0	Diabetes type II_ny	Nej	3566	0	100.0
		Ja	175	0	.0
	Overall Percentage				95.3

a. Constant is included in the model.

b. The cut value is ,500

Variables in the Equation

		B	S.E.	Wald	df	Sig.	Exp(B)
Step 0	Constant	-3.014	.077	1515.784	1	.000	.049

Variables not in the Equation

			Score	df	Sig.
Step 0	Variables	Cluster_model_men	10.750	3	.013
		Cluster_model_men(1)	9.864	1	.002
		Cluster_model_men(2)	.715	1	.398
		Cluster_model_men(3)	.061	1	.805
		fodelselandmammapappa_ny(1)	.962	1	.327
		utbildningmammapappa_ny(1)	55.916	1	<.001
		Birthyear_decades	171.708	5	<.001
		Birthyear_decades(1)	32.272	1	<.001
		Birthyear_decades(2)	26.716	1	<.001
		Birthyear_decades(3)	.027	1	.869
		Birthyear_decades(4)	45.415	1	<.001
		Birthyear_decades(5)	75.936	1	<.001
	Overall Statistics		177.074	10	<.001

Block 1: Method = Enter

Omnibus Tests of Model Coefficients

		Chi-square	df	Sig.
Step 1	Step	196.254	10	<.001

	Block	196.254	10	<.001
	Model	196.254	10	<.001

Model Summary

Step	-2 Log likelihood	Cox & Snell R Square	Nagelkerke R Square
1	1217.242[a]	.051	.162

a. Estimation terminated at iteration number 9 because parameter estimates changed by less than ,001.

Classification Table[a]

	Observed		Predicted: Diabetes type II_ny Nej	Predicted: Diabetes type II_ny Ja	Predicted: Percentage Correct
Step 1	Diabetes type II_ny	Nej	3566	0	100.0
		Ja	175	0	.0
	Overall Percentage				95.3

a. The cut value is ,500

Variables in the Equation

		B	S.E.	Wald	df	Sig.	Exp(B)	95% C.I.for EXP(B) Lower	95% C.I.for EXP(B) Upper
Step 1[a]	Cluster_model_men			3.342	3	.342			
	Cluster_model_men(1)	-.132	.178	.543	1	.461	.877	.618	1.244
	Cluster_model_men(2)	.371	.282	1.729	1	.188	1.450	.834	2.521
	Cluster_model_men(3)	.221	.370	.355	1	.551	1.247	.603	2.577
	fodelselandmammapappa_ny(1)	.040	.382	.011	1	.916	1.041	.493	2.200
	utbildningmammapappa_ny(1)	.331	.197	2.828	1	.093	1.393	.947	2.050
	Birthyear_decades			72.002	5	<.001			
	Birthyear_decades(1)	-.318	1.416	.050	1	.822	.728	.045	11.676
	Birthyear_decades(2)	1.264	1.085	1.358	1	.244	3.539	.422	29.666
	Birthyear_decades(3)	2.941	1.022	8.278	1	.004	18.928	2.553	140.304
	Birthyear_decades(4)	3.544	1.017	12.146	1	<.001	34.618	4.716	254.095
	Birthyear_decades(5)	4.003	1.022	15.357	1	<.001	54.781	7.397	405.699
	Constant	-6.140	1.007	37.194	1	<.001	.002		

a. Variable(s) entered on step 1: Cluster_model_men, fodelselandmammapappa_ny, utbildningmammapappa_ny, Birthyear_decades.

Logistic Regression

Notes

Output Created		26-AUG-2025 11:44:29
Comments		
Input	Data	/Users/stevenlc/Library/CloudStorage/OneDrive-Privat/ICloud filer/Doktorander/Rickard/Artikel 4/Menfour class model.sav
	Active Dataset	DataSet3
	File Label	Scored Data File
	Filter	<none>
	Weight	<none>
	Split File	<none>
	N of Rows in Working Data File	4656
Missing Value Handling	Definition of Missing	User-defined missing values are treated as missing
Syntax		LOGISTIC REGRESSION VARIABLES Tumörsjd_ny /METHOD=ENTER Cluster_model_men fodelselandmammapappa_ny utbildningmammapappa_ny Birthyear_decades /CONTRAST (Cluster_model_men)=Indicator(1) /CONTRAST (fodelselandmammapappa_ny)=Indicator(1) /CONTRAST (utbildningmammapappa_ny)=Indicator(1) /CONTRAST (Birthyear_decades)=Indicator(1) /PRINT=CI(95) /CRITERIA=PIN(0.05) POUT(0.10) ITERATE(20) CUT(0.5).
Resources	Processor Time	00:00:00,23

Elapsed Time	00:00:00,00

Case Processing Summary

Unweighted Cases[a]		N	Percent
Selected Cases	Included in Analysis	3741	80.3
	Missing Cases	915	19.7
	Total	4656	100.0
Unselected Cases		0	.0
Total		4656	100.0

a. If weight is in effect, see classification table for the total number of cases.

Dependent Variable Encoding

Original Value	Internal Value
Nej	0
Ja	1

Categorical Variables Codings

			Parameter coding				
		Frequency	(1)	(2)	(3)	(4)	(5)
Birthyear_decades	18-27	458	.000	.000	.000	.000	.000
	28-37	595	1.000	.000	.000	.000	.000
	38-47	678	.000	1.000	.000	.000	.000
	48-57	709	.000	.000	1.000	.000	.000
	58-67	895	.000	.000	.000	1.000	.000
	68-74	406	.000	.000	.000	.000	1.000
Cluster_model_men	1.00	1606	.000	.000	.000		
	2.00	1627	1.000	.000	.000		
	3.00	300	.000	1.000	.000		
	4.00	208	.000	.000	1.000		
utbildningmammapappa_ny	Minst en förälder högre utb än grundskola	1866	.000				
	Båda föräldrarna grundskola	1875	1.000				
fodelselandmammapappa_ny	Minst en förälder född i norden	3504	.000				
	Båda födda utanför Norden	237	1.000				

Block 0: Beginning Block

Classification Table[a,b]

Observed			Predicted Cancer_ny Nej	Predicted Cancer_ny Ja	Predicted Percentage Correct
Step 0	Cancer_ny	Nej	3588	0	100.0
		Ja	153	0	.0
	Overall Percentage				95.9

a. Constant is included in the model.

b. The cut value is ,500

Variables in the Equation

		B	S.E.	Wald	df	Sig.	Exp(B)
Step 0	Constant	-3.155	.083	1460.598	1	.000	.043

Variables not in the Equation

			Score	df	Sig.
Step 0	Variables	Cluster_model_men	5.409	3	.144
		Cluster_model_men(1)	.179	1	.672
		Cluster_model_men(2)	3.034	1	.082
		Cluster_model_men(3)	2.636	1	.104
		fodelselandmammapappa_ny(1)	.329	1	.566
		utbildningmammapappa_ny(1)	17.470	1	<.001
		Birthyear_decades	81.778	5	<.001
		Birthyear_decades(1)	11.981	1	<.001
		Birthyear_decades(2)	7.441	1	.006
		Birthyear_decades(3)	.177	1	.674
		Birthyear_decades(4)	12.671	1	<.001
		Birthyear_decades(5)	49.074	1	<.001
	Overall Statistics		88.917	10	<.001

Block 1: Method = Enter

Omnibus Tests of Model Coefficients

		Chi-square	df	Sig.
Step 1	Step	84.873	10	<.001

	Block	84.873	10	<.001
	Model	84.873	10	<.001

Model Summary

Step	-2 Log likelihood	Cox & Snell R Square	Nagelkerke R Square
1	1192.964[a]	.022	.078

a. Estimation terminated at iteration number 7 because parameter estimates changed by less than ,001.

Classification Table[a]

Observed			Predicted Cancer_ny Nej	Ja	Percentage Correct
Step 1	Cancer_ny	Nej	3588	0	100.0
		Ja	153	0	.0
	Overall Percentage				95.9

a. The cut value is ,500

Variables in the Equation

		B	S.E.	Wald	df	Sig.	Exp(B)	95% C.I.for EXP(B) Lower	Upper
Step 1[a]	Cluster_model_men			7.498	3	.058			
	Cluster_model_men(1)	.240	.184	1.695	1	.193	1.271	.886	1.822
	Cluster_model_men(2)	.662	.281	5.572	1	.018	1.940	1.119	3.362
	Cluster_model_men(3)	-.465	.526	.781	1	.377	.628	.224	1.761
	fodelselandmammapappa_ny(1)	.012	.378	.001	1	.974	1.012	.483	2.122
	utbildningmammapappa_ny(1)	.049	.196	.064	1	.800	1.051	.716	1.542
	Birthyear_decades			56.876	5	<.001			
	Birthyear_decades(1)	.521	.605	.741	1	.389	1.683	.514	5.512
	Birthyear_decades(2)	.925	.570	2.634	1	.105	2.523	.825	7.713
	Birthyear_decades(3)	1.482	.548	7.301	1	.007	4.402	1.502	12.897
	Birthyear_decades(4)	2.004	.538	13.889	1	<.001	7.418	2.586	21.282
	Birthyear_decades(5)	2.618	.546	22.990	1	<.001	13.711	4.702	39.981
	Constant	-4.903	.517	90.002	1	<.001	.007		

a. Variable(s) entered on step 1: Cluster_model_men, fodelselandmammapappa_ny, utbildningmammapappa_ny, Birthyear_decades.

Logistic Regression

Notes

Output Created		26-AUG-2025 11:44:29
Comments		
Input	Data	/Users/stevenlc/Library/CloudStorage/OneDrive-Privat/ICloud filer/Doktorander/Rickard/Artikel 4/Menfour class model.sav
	Active Dataset	DataSet3
	File Label	Scored Data File
	Filter	<none>
	Weight	<none>
	Split File	<none>
	N of Rows in Working Data File	4656
Missing Value Handling	Definition of Missing	User-defined missing values are treated as missing
Syntax		LOGISTIC REGRESSION VARIABLES Fetma_ny /METHOD=ENTER Cluster_model_men fodelselandmammapappa_ny utbildningmammapappa_ny Birthyear_decades /CONTRAST (Cluster_model_men)=Indicator(1) /CONTRAST (fodelselandmammapappa_ny)=Indicator(1) /CONTRAST (utbildningmammapappa_ny)=Indicator(1) /CONTRAST (Birthyear_decades)=Indicator(1) /PRINT=CI(95) /CRITERIA=PIN(0.05) POUT(0.10) ITERATE(20) CUT(0.5).
Resources	Processor Time	00:00:00,21

Elapsed Time	00:00:00,00

Case Processing Summary

Unweighted Cases[a]		N	Percent
Selected Cases	Included in Analysis	3694	79.3
	Missing Cases	962	20.7
	Total	4656	100.0
Unselected Cases		0	.0
Total		4656	100.0

a. If weight is in effect, see classification table for the total number of cases.

Dependent Variable Encoding

Original Value	Internal Value
Nej	0
Ja	1

Categorical Variables Codings

			Parameter coding				
		Frequency	(1)	(2)	(3)	(4)	(5)
Birthyear_decades	18-27	452	.000	.000	.000	.000	.000
	28-37	587	1.000	.000	.000	.000	.000
	38-47	669	.000	1.000	.000	.000	.000
	48-57	704	.000	.000	1.000	.000	.000
	58-67	884	.000	.000	.000	1.000	.000
	68-74	398	.000	.000	.000	.000	1.000
Cluster_model_men	1.00	1580	.000	.000	.000		
	2.00	1610	1.000	.000	.000		
	3.00	298	.000	1.000	.000		
	4.00	206	.000	.000	1.000		
utbildningmammapappa_ny	Minst en förälder högre utb än grundskola	1843	.000				
	Båda föräldrarna grundskola	1851	1.000				
fodelselandmammapappa_ny	Minst en förälder född i norden	3462	.000				
	Båda födda utanför Norden	232	1.000				

Block 0: Beginning Block

Classification Table[a,b]

Observed			Predicted Obesity_ny Nej	Predicted Obesity_ny Ja	Percentage Correct
Step 0	Obesity_ny	Nej	3178	0	100.0
		Ja	516	0	.0
	Overall Percentage				86.0

a. Constant is included in the model.

b. The cut value is ,500

Variables in the Equation

		B	S.E.	Wald	df	Sig.	Exp(B)
Step 0	Constant	-1.818	.047	1467.057	1	.000	.162

Variables not in the Equation

			Score	df	Sig.
Step 0	Variables	Cluster_model_men	5.729	3	.126
		Cluster_model_men(1)	4.392	1	.036
		Cluster_model_men(2)	2.669	1	.102
		Cluster_model_men(3)	.002	1	.963
		fodelselandmammapappa_ny(1)	5.891	1	.015
		utbildningmammapappa_ny(1)	30.775	1	<.001
		Birthyear_decades	53.059	5	<.001
		Birthyear_decades(1)	16.192	1	<.001
		Birthyear_decades(2)	.468	1	.494
		Birthyear_decades(3)	10.380	1	.001
		Birthyear_decades(4)	4.714	1	.030
		Birthyear_decades(5)	4.862	1	.027
	Overall Statistics		66.543	10	<.001

Block 1: Method = Enter

Omnibus Tests of Model Coefficients

		Chi-square	df	Sig.
Step 1	Step	72.787	10	<.001

	Block	72.787	10	<.001
	Model	72.787	10	<.001

Model Summary

Step	-2 Log likelihood	Cox & Snell R Square	Nagelkerke R Square
1	2914.869[a]	.020	.035

a. Estimation terminated at iteration number 5 because parameter estimates changed by less than ,001.

Classification Table[a]

Observed			Predicted Obesity_ny Nej	Ja	Percentage Correct
Step 1	Obesity_ny	Nej	3178	0	100.0
		Ja	516	0	.0
	Overall Percentage				86.0

a. The cut value is ,500

Variables in the Equation

		B	S.E.	Wald	df	Sig.	Exp(B)	95% C.I.for EXP(B) Lower	Upper
Step 1[a]	Cluster_model_men			4.054	3	.256			
	Cluster_model_men(1)	-.075	.106	.502	1	.479	.927	.753	1.142
	Cluster_model_men(2)	.272	.173	2.477	1	.116	1.312	.936	1.840
	Cluster_model_men(3)	.035	.216	.027	1	.870	1.036	.678	1.583
	fodelselandmammapappa_ny(1)	-.470	.242	3.784	1	.052	.625	.389	1.004
	utbildningmammapappa_ny(1)	.258	.111	5.416	1	.020	1.294	1.042	1.608
	Birthyear_decades			29.451	5	<.001			
	Birthyear_decades(1)	.337	.245	1.893	1	.169	1.401	.867	2.265
	Birthyear_decades(2)	.859	.228	14.226	1	<.001	2.360	1.510	3.686
	Birthyear_decades(3)	1.028	.227	20.567	1	<.001	2.797	1.793	4.362
	Birthyear_decades(4)	.871	.229	14.471	1	<.001	2.389	1.525	3.742
	Birthyear_decades(5)	.958	.249	14.784	1	<.001	2.608	1.600	4.250
	Constant	-2.695	.205	172.170	1	<.001	.068		

a. Variable(s) entered on step 1: Cluster_model_men, fodelselandmammapappa_ny, utbildningmammapappa_ny, Birthyear_decades.

Logistic Regression

Notes

Output Created		26-AUG-2025 11:44:29
Comments		
Input	Data	/Users/stevenlc/Library/CloudStorage/OneDrive-Privat/ICloud filer/Doktorander/Rickard/Artikel 4/Menfour class model.sav
	Active Dataset	DataSet3
	File Label	Scored Data File
	Filter	<none>
	Weight	<none>
	Split File	<none>
	N of Rows in Working Data File	4656
Missing Value Handling	Definition of Missing	User-defined missing values are treated as missing
Syntax		LOGISTIC REGRESSION VARIABLES Storrökare_ny<br>/METHOD=ENTER Cluster_model_men fodelselandmammapappa_ny utbildningmammapappa_ny Birthyear_decades<br>/CONTRAST (Cluster_model_men)=Indicator(1)<br>/CONTRAST (fodelselandmammapappa_ny)=Indicator(1)<br>/CONTRAST (utbildningmammapappa_ny)=Indicator(1)<br>/CONTRAST (Birthyear_decades)=Indicator(1)<br>/PRINT=CI(95)<br>/CRITERIA=PIN(0.05) POUT(0.10) ITERATE(20) CUT(0.5).
Resources	Processor Time	00:00:00,21

Elapsed Time	00:00:00,00

Case Processing Summary

Unweighted Cases[a]		N	Percent
Selected Cases	Included in Analysis	3702	79.5
	Missing Cases	954	20.5
	Total	4656	100.0
Unselected Cases		0	.0
Total		4656	100.0

a. If weight is in effect, see classification table for the total number of cases.

Dependent Variable Encoding

Original Value	Internal Value
Nej	0
Ja	1

Categorical Variables Codings

		Frequency	Parameter coding (1)	(2)	(3)	(4)	(5)
Birthyear_decades	18-27	457	.000	.000	.000	.000	.000
	28-37	593	1.000	.000	.000	.000	.000
	38-47	673	.000	1.000	.000	.000	.000
	48-57	704	.000	.000	1.000	.000	.000
	58-67	885	.000	.000	.000	1.000	.000
	68-74	390	.000	.000	.000	.000	1.000
Cluster_model_men	1.00	1584	.000	.000	.000		
	2.00	1614	1.000	.000	.000		
	3.00	299	.000	1.000	.000		
	4.00	205	.000	.000	1.000		
utbildningmammapappa_ny	Minst en förälder högre utb än grundskola	1850	.000				
	Båda föräldrarna grundskola	1852	1.000				
fodelselandmammapappa_ny	Minst en förälder född i norden	3469	.000				
	Båda födda utanför Norden	233	1.000				

Block 0: Beginning Block

Classification Table[a,b]

Observed			Predicted: Heavy smoking_ny Nej	Predicted: Heavy smoking_ny Ja	Predicted: Percentage Correct
Step 0	Heavy smoking_ny	Nej	3504	0	100.0
		Ja	198	0	.0
	Overall Percentage				94.7

a. Constant is included in the model.

b. The cut value is ,500

Variables in the Equation

		B	S.E.	Wald	df	Sig.	Exp(B)
Step 0	Constant	-2.873	.073	1547.330	1	.000	.057

Variables not in the Equation

			Score	df	Sig.
Step 0	Variables	Cluster_model_men	13.947	3	.003
		Cluster_model_men(1)	.038	1	.845
		Cluster_model_men(2)	12.161	1	<.001
		Cluster_model_men(3)	.423	1	.516
		fodelselandmammapappa_ny(1)	10.047	1	.002
		utbildningmammapappa_ny(1)	11.238	1	<.001
		Birthyear_decades	83.383	5	<.001
		Birthyear_decades(1)	17.023	1	<.001
		Birthyear_decades(2)	14.342	1	<.001
		Birthyear_decades(3)	.991	1	.320
		Birthyear_decades(4)	53.397	1	<.001
		Birthyear_decades(5)	4.731	1	.030
	Overall Statistics		119.595	10	<.001

Block 1: Method = Enter

Omnibus Tests of Model Coefficients

		Chi-square	df	Sig.
Step 1	Step	118.669	10	<.001

	Block	118.669	10	<.001
	Model	118.669	10	<.001

Model Summary

Step	-2 Log likelihood	Cox & Snell R Square	Nagelkerke R Square
1	1426.179[a]	.032	.092

a. Estimation terminated at iteration number 7 because parameter estimates changed by less than ,001.

Classification Table[a]

Observed			Predicted Heavy smoking_ny Nej	Predicted Heavy smoking_ny Ja	Percentage Correct
Step 1	Heavy smoking_ny	Nej	3504	0	100.0
		Ja	198	0	.0
	Overall Percentage				94.7

a. The cut value is ,500

Variables in the Equation

		B	S.E.	Wald	df	Sig.	Exp(B)	95% C.I.for EXP(B) Lower	95% C.I.for EXP(B) Upper
Step 1[a]	Cluster_model_men			19.562	3	<.001			
	Cluster_model_men(1)	.425	.169	6.306	1	.012	1.530	1.098	2.133
	Cluster_model_men(2)	1.010	.238	17.944	1	<.001	2.746	1.721	4.383
	Cluster_model_men(3)	.634	.321	3.894	1	.048	1.884	1.004	3.535
	fodelselandmammapappa_ny(1)	.892	.246	13.126	1	<.001	2.439	1.506	3.951
	utbildningmammapappa_ny(1)	-.056	.170	.110	1	.740	.945	.678	1.318
	Birthyear_decades			71.979	5	<.001			
	Birthyear_decades(1)	-.048	.471	.010	1	.919	.953	.379	2.399
	Birthyear_decades(2)	.306	.441	.479	1	.489	1.357	.572	3.223
	Birthyear_decades(3)	1.339	.399	11.284	1	<.001	3.816	1.747	8.336
	Birthyear_decades(4)	1.972	.389	25.671	1	<.001	7.185	3.351	15.406
	Birthyear_decades(5)	1.751	.422	17.230	1	<.001	5.762	2.520	13.172
	Constant	-4.469	.377	140.176	1	<.001	.011		

a. Variable(s) entered on step 1: Cluster_model_men, fodelselandmammapappa_ny, utbildningmammapappa_ny, Birthyear_decades.

Logistic Regression

Notes

Output Created		26-AUG-2025 11:44:29
Comments		
Input	Data	/Users/stevenlc/Library/CloudStorage/OneDrive-Privat/ICloud filer/Doktorander/Rickard/Artikel 4/Menfour class model.sav
	Active Dataset	DataSet3
	File Label	Scored Data File
	Filter	<none>
	Weight	<none>
	Split File	<none>
	N of Rows in Working Data File	4656
Missing Value Handling	Definition of Missing	User-defined missing values are treated as missing
Syntax		LOGISTIC REGRESSION VARIABLES AUDITRISK_inknykt_ny /METHOD=ENTER Cluster_model_men fodelselandmammapappa_ny utbildningmammapappa_ny Birthyear_decades /CONTRAST (Cluster_model_men)=Indicator(1) /CONTRAST (fodelselandmammapappa_ny)=Indicator(1) /CONTRAST (utbildningmammapappa_ny)=Indicator(1) /CONTRAST (Birthyear_decades)=Indicator(1) /PRINT=CI(95) /CRITERIA=PIN(0.05) POUT(0.10) ITERATE(20) CUT(0.5).

Resources	Processor Time	00:00:00,21
	Elapsed Time	00:00:00,00

Case Processing Summary

Unweighted Cases[a]		N	Percent
Selected Cases	Included in Analysis	3649	78.4
	Missing Cases	1007	21.6
	Total	4656	100.0
Unselected Cases		0	.0
Total		4656	100.0

a. If weight is in effect, see classification table for the total number of cases.

Dependent Variable Encoding

Original Value	Internal Value
Nej	0
Ja	1

Categorical Variables Codings

		Frequency	Parameter coding (1)	(2)	(3)	(4)	(5)
Birthyear_decades	18-27	452	.000	.000	.000	.000	.000
	28-37	586	1.000	.000	.000	.000	.000
	38-47	665	.000	1.000	.000	.000	.000
	48-57	694	.000	.000	1.000	.000	.000
	58-67	861	.000	.000	.000	1.000	.000
	68-74	391	.000	.000	.000	.000	1.000
Cluster_model_men	1.00	1551	.000	.000	.000		
	2.00	1597	1.000	.000	.000		
	3.00	296	.000	1.000	.000		
	4.00	205	.000	.000	1.000		
utbildningmammapappa_ny	Minst en förälder högre utb än grundskola	1833	.000				
	Båda föräldrarna grundskola	1816	1.000				
fodelselandmammapappa_ny	Minst en förälder född i norden	3415	.000				
	Båda födda utanför Norden	234	1.000				

Block 0: Beginning Block

Classification Table[a,b]

Observed			Predicted: Heavy drinking_ny Nej	Predicted: Heavy drinking_ny Ja	Predicted: Percentage Correct
Step 0	Heavy drinking_ny	Nej	2898	0	100.0
		Ja	751	0	.0
	Overall Percentage				79.4

a. Constant is included in the model.

b. The cut value is ,500

Variables in the Equation

		B	S.E.	Wald	df	Sig.	Exp(B)
Step 0	Constant	-1.350	.041	1087.603	1	<.001	.259

Variables not in the Equation

			Score	df	Sig.
Step 0	Variables	Cluster_model_men	120.702	3	<.001
		Cluster_model_men(1)	23.973	1	<.001
		Cluster_model_men(2)	15.300	1	<.001
		Cluster_model_men(3)	38.313	1	<.001
		fodelselandmammapappa_ny(1)	12.508	1	<.001
		utbildningmammapappa_ny(1)	57.695	1	<.001
		Birthyear_decades	132.429	5	<.001
		Birthyear_decades(1)	14.714	1	<.001
		Birthyear_decades(2)	.884	1	.347
		Birthyear_decades(3)	4.724	1	.030
		Birthyear_decades(4)	19.002	1	<.001
		Birthyear_decades(5)	23.311	1	<.001
	Overall Statistics		255.293	10	<.001

Block 1: Method = Enter

Omnibus Tests of Model Coefficients

	Chi-square	df	Sig.

Step 1	Step	252.590	10	<.001
	Block	252.590	10	<.001
	Model	252.590	10	<.001

Model Summary

Step	-2 Log likelihood	Cox & Snell R Square	Nagelkerke R Square
1	3457.362[a]	.067	.105

a. Estimation terminated at iteration number 5 because parameter estimates changed by less than ,001.

Classification Table[a]

Observed			Predicted: Heavy drinking_ny Nej	Predicted: Heavy drinking_ny Ja	Percentage Correct
Step 1	Heavy drinking_ny	Nej	2881	17	99.4
		Ja	729	22	2.9
	Overall Percentage				79.6

a. The cut value is ,500

Variables in the Equation

		B	S.E.	Wald	df	Sig.	Exp(B)	95% C.I.for EXP(B) Lower	95% C.I.for EXP(B) Upper
Step 1[a]	Cluster_model_men			97.268	3	<.001			
	Cluster_model_men(1)	.667	.099	45.209	1	<.001	1.948	1.604	2.366
	Cluster_model_men(2)	1.048	.153	47.012	1	<.001	2.853	2.114	3.850
	Cluster_model_men(3)	1.381	.168	67.417	1	<.001	3.980	2.862	5.534
	fodelselandmammapappa_ny(1)	-.898	.215	17.498	1	<.001	.407	.267	.620
	utbildningmammapappa_ny(1)	-.193	.101	3.647	1	.056	.824	.676	1.005
	Birthyear_decades			76.821	5	<.001			
	Birthyear_decades(1)	-.626	.139	20.240	1	<.001	.535	.407	.702
	Birthyear_decades(2)	-1.018	.146	48.691	1	<.001	.361	.272	.481
	Birthyear_decades(3)	-1.018	.151	45.651	1	<.001	.361	.269	.485
	Birthyear_decades(4)	-1.069	.154	48.339	1	<.001	.343	.254	.464
	Birthyear_decades(5)	-1.357	.203	44.819	1	<.001	.257	.173	.383
	Constant	-.898	.119	57.271	1	<.001	.407		

a. Variable(s) entered on step 1: Cluster_model_men, fodelselandmammapappa_ny, utbildningmammapappa_ny, Birthyear_decades.

Logistic Regression

Notes

Output Created		26-AUG-2025 11:44:29
Comments		
Input	Data	/Users/stevenlc/Library/CloudStorage/OneDrive-Privat/ICloud filer/Doktorander/Rickard/Artikel 4/Menfour class model.sav
	Active Dataset	DataSet3
	File Label	Scored Data File
	Filter	<none>
	Weight	<none>
	Split File	<none>
	N of Rows in Working Data File	4656
Missing Value Handling	Definition of Missing	User-defined missing values are treated as missing

Syntax		LOGISTIC REGRESSION VARIABLES Drogmissbruk_ny /METHOD=ENTER Cluster_model_men fodelselandmammapappa_ny utbildningmammapappa_ny Birthyear_decades /CONTRAST (Cluster_model_men)=Indicator(1) /CONTRAST (fodelselandmammapappa_ny)=Indicator(1) /CONTRAST (utbildningmammapappa_ny)=Indicator(1) /CONTRAST (Birthyear_decades)=Indicator(1) /PRINT=CI(95) /CRITERIA=PIN(0.05) POUT(0.10) ITERATE(20) CUT(0.5).
Resources	Processor Time	00:00:00,22
	Elapsed Time	00:00:01,00

Case Processing Summary

Unweighted Cases[a]		N	Percent
Selected Cases	Included in Analysis	3741	80.3
	Missing Cases	915	19.7
	Total	4656	100.0
Unselected Cases		0	.0
Total		4656	100.0

a. If weight is in effect, see classification table for the total number of cases.

Dependent Variable Encoding

Original Value	Internal Value
Nej	0
Ja	1

Categorical Variables Codings

		Frequency	Parameter coding (1)	(2)	(3)	(4)	(5)
Birthyear_decades	18-27	458	.000	.000	.000	.000	.000
	28-37	595	1.000	.000	.000	.000	.000
	38-47	678	.000	1.000	.000	.000	.000
	48-57	709	.000	.000	1.000	.000	.000
	58-67	895	.000	.000	.000	1.000	.000
	68-74	406	.000	.000	.000	.000	1.000
Cluster_model_men	1.00	1606	.000	.000	.000		
	2.00	1627	1.000	.000	.000		
	3.00	300	.000	1.000	.000		
	4.00	208	.000	.000	1.000		
utbildningmammapappa_ny	Minst en förälder högre utb än grundskola	1866	.000				
	Båda föräldrarna grundskola	1875	1.000				
fodelselandmammapappa_ny	Minst en förälder född i norden	3504	.000				
	Båda födda utanför Norden	237	1.000				

Block 0: Beginning Block

Classification Table[a,b]

Observed			Predicted Drug abuse_ny Nej	Ja	Percentage Correct
Step 0	Drug abuse_ny	Nej	3720	0	100.0
		Ja	21	0	.0
	Overall Percentage				99.4

a. Constant is included in the model.

b. The cut value is ,500

Variables in the Equation

		B	S.E.	Wald	df	Sig.	Exp(B)
Step 0	Constant	-5.177	.219	559.659	1	<.001	.006

Variables not in the Equation

	Score	df	Sig.

			Score	df	Sig.
Step 0	Variables	Cluster_model_men	31.093	3	<.001
		Cluster_model_men(1)	.887	1	.346
		Cluster_model_men(2)	.304	1	.582
		Cluster_model_men(3)	31.023	1	<.001
		fodelselandmammapappa_ny(1)	.088	1	.767
		utbildningmammapappa_ny(1)	.053	1	.818
		Birthyear_decades	3.543	5	.617
		Birthyear_decades(1)	.156	1	.693
		Birthyear_decades(2)	.210	1	.647
		Birthyear_decades(3)	.000	1	.991
		Birthyear_decades(4)	.276	1	.599
		Birthyear_decades(5)	.810	1	.368
	Overall Statistics		35.865	10	<.001

Block 1: Method = Enter

Omnibus Tests of Model Coefficients

		Chi-square	df	Sig.
Step 1	Step	20.147	10	.028
	Block	20.147	10	.028
	Model	20.147	10	.028

Model Summary

Step	-2 Log likelihood	Cox & Snell R Square	Nagelkerke R Square
1	239.404[a]	.005	.080

a. Estimation terminated at iteration number 9 because parameter estimates changed by less than ,001.

Classification Table[a]

Observed			Predicted: Drug abuse_ny Nej	Predicted: Drug abuse_ny Ja	Predicted: Percentage Correct
Step 1	Drug abuse_ny	Nej	3720	0	100.0
		Ja	21	0	.0
	Overall Percentage				99.4

a. The cut value is ,500

Variables in the Equation

		B	S.E.	Wald	df	Sig.	Exp(B)	95% C.I.for EXP(B) Lower	Upper
Step 1[a]	Cluster_model_men			22.287	3	<.001			
	Cluster_model_men(1)	.092	.566	.027	1	.870	1.097	.362	3.324
	Cluster_model_men(2)	-.092	1.086	.007	1	.932	.912	.108	7.668
	Cluster_model_men(3)	2.298	.578	15.826	1	<.001	9.953	3.208	30.874
	fodelselandmammapappa_ny(1)	-.378	1.036	.133	1	.715	.685	.090	5.220
	utbildningmammapappa_ny(1)	.484	.529	.837	1	.360	1.623	.575	4.581
	Birthyear_decades			4.655	5	.459			
	Birthyear_decades(1)	-.746	.689	1.174	1	.279	.474	.123	1.829
	Birthyear_decades(2)	-1.373	.776	3.129	1	.077	.253	.055	1.160
	Birthyear_decades(3)	-1.046	.740	1.997	1	.158	.351	.082	1.499
	Birthyear_decades(4)	-1.284	.772	2.769	1	.096	.277	.061	1.257
	Birthyear_decades(5)	-1.802	1.171	2.367	1	.124	.165	.017	1.638
	Constant	-4.829	.584	68.355	1	<.001	.008		

a. Variable(s) entered on step 1: Cluster_model_men, fodelselandmammapappa_ny, utbildningmammapappa_ny, Birthyear_decades.

Logistic Regression

Notes

Output Created		26-AUG-2025 11:46:46
Comments		
Input	Data	/Users/stevenlc/Library/CloudStorage/OneDrive-Privat/ICloud filer/Doktorander/Rickard/Artikel 4/Rickardonlywomen7classmodel.sav
	Active Dataset	DataSet4
	File Label	Scored Data File
	Filter	<none>
	Weight	<none>
	Split File	<none>
	N of Rows in Working Data File	5681

Missing Value Handling	Definition of Missing	User-defined missing values are treated as missing
Syntax		LOGISTIC REGRESSION VARIABLES HAD_probable_depression_ny<br>/METHOD=ENTER clu#<br>/CONTRAST (clu#)=INDICATOR(1)<br>/PRINT=CI(95)<br>/CRITERIA=PIN(0.05) POUT(0.10) ITERATE(20) CUT(0.5).
Resources	Processor Time	00:00:00,25
	Elapsed Time	00:00:00,00

```
[DataSet4] /Users/stevenlc/Library/CloudStorage/OneDrive-Privat/ICloud
filer/Doktorander/Rickard/Artikel 4/Rickardonlywomen7classmodel.sav
```

Case Processing Summary

Unweighted Cases[a]		N	Percent
Selected Cases	Included in Analysis	4898	86.2
	Missing Cases	783	13.8
	Total	5681	100.0
Unselected Cases		0	.0
Total		5681	100.0

a. If weight is in effect, see classification table for the total number of cases.

Dependent Variable Encoding

Original Value	Internal Value
Nej	0
Ja	1

Categorical Variables Codings

			Parameter coding					
		Frequency	(1)	(2)	(3)	(4)	(5)	(6)
Cluster modal	1	2251	.000	.000	.000	.000	.000	.000
	2	1028	1.000	.000	.000	.000	.000	.000

	3	503	.000	1.000	.000	.000	.000	.000
	4	371	.000	.000	1.000	.000	.000	.000
	5	272	.000	.000	.000	1.000	.000	.000
	6	276	.000	.000	.000	.000	1.000	.000
	7	197	.000	.000	.000	.000	.000	1.000

Block 0: Beginning Block

Classification Table[a,b]

Observed			Predicted		
			Depression_ny		Percentage Correct
			Nej	Ja	
Step 0	Depression_ny	Nej	4451	0	100.0
		Ja	447	0	.0
	Overall Percentage				90.9

a. Constant is included in the model.
b. The cut value is ,500

Variables in the Equation

		B	S.E.	Wald	df	Sig.	Exp(B)
Step 0	Constant	-2.298	.050	2145.702	1	.000	.100

Variables not in the Equation

			Score	df	Sig.
Step 0	Variables	Cluster modal	192.753	6	<.001
		Cluster modal(1)	1.154	1	.283
		Cluster modal(2)	.000	1	.988
		Cluster modal(3)	56.663	1	<.001
		Cluster modal(4)	3.138	1	.076
		Cluster modal(5)	46.852	1	<.001
		Cluster modal(6)	43.181	1	<.001
	Overall Statistics		192.753	6	<.001

Block 1: Method = Enter

Omnibus Tests of Model Coefficients

		Chi-square	df	Sig.
Step 1	Step	169.007	6	<.001
	Block	169.007	6	<.001
	Model	169.007	6	<.001

Model Summary

Step	-2 Log likelihood	Cox & Snell R Square	Nagelkerke R Square
1	2823.155[a]	.034	.074

a. Estimation terminated at iteration number 6 because parameter estimates changed by less than ,001.

Classification Table[a]

Observed			Predicted Depression_ny Nej	Ja	Percentage Correct
Step 1	Depression_ny	Nej	4451	0	100.0
		Ja	447	0	.0
	Overall Percentage				90.9

a. The cut value is ,500

Variables in the Equation

		B	S.E.	Wald	df	Sig.	Exp(B)	95% C.I.for EXP(B) Lower	Upper
Step 1[a]	Cluster modal			170.360	6	<.001			
	Cluster modal(1)	.581	.150	14.990	1	<.001	1.789	1.333	2.401
	Cluster modal(2)	.692	.183	14.221	1	<.001	1.997	1.394	2.861
	Cluster modal(3)	1.598	.163	95.997	1	<.001	4.944	3.591	6.806
	Cluster modal(4)	1.008	.210	22.975	1	<.001	2.740	1.814	4.137
	Cluster modal(5)	1.642	.178	84.670	1	<.001	5.165	3.640	7.327
	Cluster modal(6)	1.742	.197	77.794	1	<.001	5.706	3.875	8.403
	Constant	-2.988	.099	917.873	1	<.001	.050		

a. Variable(s) entered on step 1: Cluster modal.

Logistic Regression

Notes

Output Created		26-AUG-2025 11:46:46
Comments		
Input	Data	/Users/stevenlc/Library/CloudStorage/OneDrive-Privat/ICloud filer/Doktorander/Rickard/Artikel 4/Rickardonlywomen7classmodel.sav
	Active Dataset	DataSet4
	File Label	Scored Data File
	Filter	<none>
	Weight	<none>
	Split File	<none>
	N of Rows in Working Data File	5681
Missing Value Handling	Definition of Missing	User-defined missing values are treated as missing
Syntax		LOGISTIC REGRESSION VARIABLES HAD_probable_ångest_ny<br>/METHOD=ENTER clu#<br>/CONTRAST (clu#)=INDICATOR(1)<br>/PRINT=CI(95)<br>/CRITERIA=PIN(0.05) POUT(0.10) ITERATE(20) CUT(0.5).
Resources	Processor Time	00:00:00,24
	Elapsed Time	00:00:01,00

Case Processing Summary

Unweighted Cases[a]		N	Percent
Selected Cases	Included in Analysis	4908	86.4
	Missing Cases	773	13.6
	Total	5681	100.0
Unselected Cases		0	.0
Total		5681	100.0

a. If weight is in effect, see classification table for the total number of cases.

Dependent Variable Encoding

Original Value	Internal Value
Nej	0
Ja	1

Categorical Variables Codings

			Parameter coding					
		Frequency	(1)	(2)	(3)	(4)	(5)	(6)
Cluster modal	1	2262	.000	.000	.000	.000	.000	.000
	2	1026	1.000	.000	.000	.000	.000	.000
	3	512	.000	1.000	.000	.000	.000	.000
	4	375	.000	.000	1.000	.000	.000	.000
	5	267	.000	.000	.000	1.000	.000	.000
	6	265	.000	.000	.000	.000	1.000	.000
	7	201	.000	.000	.000	.000	.000	1.000

Block 0: Beginning Block

Classification Table[a,b]

			Predicted		
			Anxiety_ny		Percentage Correct
Observed			Nej	Ja	
Step 0	Anxiety_ny	Nej	4575	0	100.0
		Ja	333	0	.0
	Overall Percentage				93.2

a. Constant is included in the model.

b. The cut value is ,500

Variables in the Equation

		B	S.E.	Wald	df	Sig.	Exp(B)
Step 0	Constant	-2.620	.057	2131.111	1	.000	.073

Variables not in the Equation

			Score	df	Sig.
Step 0	Variables	Cluster modal	235.214	6	<.001
		Cluster modal(1)	.614	1	.433
		Cluster modal(2)	.019	1	.891

	Cluster modal(3)	32.197	1	<.001
	Cluster modal(4)	.049	1	.825
	Cluster modal(5)	72.997	1	<.001
	Cluster modal(6)	85.906	1	<.001
	Overall Statistics	235.214	6	<.001

Block 1: Method = Enter

Omnibus Tests of Model Coefficients

		Chi-square	df	Sig.
Step 1	Step	191.388	6	<.001
	Block	191.388	6	<.001
	Model	191.388	6	<.001

Model Summary

Step	-2 Log likelihood	Cox & Snell R Square	Nagelkerke R Square
1	2243.349[a]	.038	.098

a. Estimation terminated at iteration number 6 because parameter estimates changed by less than ,001.

Classification Table[a]

Observed			Predicted: Anxiety_ny Nej	Predicted: Anxiety_ny Ja	Predicted: Percentage Correct
Step 1	Anxiety_ny	Nej	4575	0	100.0
		Ja	333	0	.0
	Overall Percentage				93.2

a. The cut value is ,500

Variables in the Equation

		B	S.E.	Wald	df	Sig.	Exp(B)	95% C.I.for EXP(B) Lower	95% C.I.for EXP(B) Upper
Step 1[a]	Cluster modal			191.605	6	<.001			
	Cluster modal(1)	.795	.180	19.565	1	<.001	2.214	1.557	3.148
	Cluster modal(2)	.861	.217	15.754	1	<.001	2.367	1.547	3.622
	Cluster modal(3)	1.678	.195	74.256	1	<.001	5.357	3.657	7.846
	Cluster modal(4)	.936	.269	12.116	1	<.001	2.549	1.505	4.317

Cluster modal(5)	2.095	.199	110.990	1	<.001	8.123	5.501	11.994
Cluster modal(6)	2.290	.209	119.730	1	<.001	9.874	6.552	14.882
Constant	-3.505	.125	787.036	1	<.001	.030		

a. Variable(s) entered on step 1: Cluster modal.

Logistic Regression

Notes

Output Created		26-AUG-2025 11:46:47
Comments		
Input	Data	/Users/stevenlc/Library/CloudStorage/OneDrive-Privat/ICloud filer/Doktorander/Rickard/Artikel 4/Rickardonlywomen7classmodel.sav
	Active Dataset	DataSet4
	File Label	Scored Data File
	Filter	<none>
	Weight	<none>
	Split File	<none>
	N of Rows in Working Data File	5681
Missing Value Handling	Definition of Missing	User-defined missing values are treated as missing
Syntax		LOGISTIC REGRESSION VARIABLES PTSD_score_pos_ny /METHOD=ENTER clu# /CONTRAST (clu#)=INDICATOR(1) /PRINT=CI(95) /CRITERIA=PIN(0.05) POUT(0.10) ITERATE(20) CUT(0.5).
Resources	Processor Time	00:00:00,24
	Elapsed Time	00:00:00,00

Case Processing Summary

Unweighted Cases[a]		N	Percent
Selected Cases	Included in Analysis	4836	85.1
	Missing Cases	845	14.9
	Total	5681	100.0
Unselected Cases		0	.0
Total		5681	100.0

a. If weight is in effect, see classification table for the total number of cases.

Dependent Variable Encoding

Original Value	Internal Value
Nej	0
Ja	1

Categorical Variables Codings

		Frequency	Parameter coding (1)	(2)	(3)	(4)	(5)	(6)
Cluster modal	1	2253	.000	.000	.000	.000	.000	.000
	2	1001	1.000	.000	.000	.000	.000	.000
	3	495	.000	1.000	.000	.000	.000	.000
	4	367	.000	.000	1.000	.000	.000	.000
	5	263	.000	.000	.000	1.000	.000	.000
	6	260	.000	.000	.000	.000	1.000	.000
	7	197	.000	.000	.000	.000	.000	1.000

Block 0: Beginning Block

Classification Table[a,b]

Observed			Predicted PTSS_ny Nej	Ja	Percentage Correct
Step 0	PTSS_ny	Nej	4392	0	100.0
		Ja	444	0	.0
	Overall Percentage				90.8

a. Constant is included in the model.
b. The cut value is ,500

Variables in the Equation

		B	S.E.	Wald	df	Sig.	Exp(B)
Step 0	Constant	-2.292	.050	2117.778	1	.000	.101

Variables not in the Equation

			Score	df	Sig.
Step 0	Variables	Cluster modal	448.456	6	<.001
		Cluster modal(1)	1.796	1	.180
		Cluster modal(2)	2.463	1	.117
		Cluster modal(3)	28.332	1	<.001
		Cluster modal(4)	4.682	1	.030
		Cluster modal(5)	74.637	1	<.001
		Cluster modal(6)	251.192	1	<.001
	Overall Statistics		448.456	6	<.001

Block 1: Method = Enter

Omnibus Tests of Model Coefficients

		Chi-square	df	Sig.
Step 1	Step	357.887	6	<.001
	Block	357.887	6	<.001
	Model	357.887	6	<.001

Model Summary

Step	-2 Log likelihood	Cox & Snell R Square	Nagelkerke R Square
1	2608.601[a]	.071	.156

a. Estimation terminated at iteration number 6 because parameter estimates changed by less than ,001.

Classification Table[a]

			Predicted		
			PTSS_ny		Percentage Correct
	Observed		Nej	Ja	
Step 1	PTSS_ny	Nej	4392	0	100.0
		Ja	444	0	.0
	Overall Percentage				90.8

a. The cut value is ,500

		B	S.E.	Wald	df	Sig.	Exp(B)	95% C.I.for EXP(B)	
								Lower	Upper
Step 1[a]	Cluster modal			328.761	6	<.001			
	Cluster modal(1)	1.040	.169	37.819	1	<.001	2.829	2.031	3.941
	Cluster modal(2)	1.390	.189	54.278	1	<.001	4.017	2.775	5.814
	Cluster modal(3)	1.877	.186	101.875	1	<.001	6.532	4.537	9.404
	Cluster modal(4)	1.563	.221	49.884	1	<.001	4.771	3.092	7.360
	Cluster modal(5)	2.330	.190	150.306	1	<.001	10.276	7.080	14.913
	Cluster modal(6)	3.111	.190	267.828	1	<.001	22.437	15.459	32.566
	Constant	-3.470	.123	794.006	1	<.001	.031		

Variables in the Equation

a. Variable(s) entered on step 1: Cluster modal.

Logistic Regression

Notes

Output Created		26-AUG-2025 11:46:47
Comments		
Input	Data	/Users/stevenlc/Library/CloudStorage/OneDrive-Privat/ICloud filer/Doktorander/Rickard/Artikel 4/Rickardonlywomen7classmodel.sav
	Active Dataset	DataSet4
	File Label	Scored Data File
	Filter	<none>
	Weight	<none>
	Split File	<none>
	N of Rows in Working Data File	5681
Missing Value Handling	Definition of Missing	User-defined missing values are treated as missing

Syntax		LOGISTIC REGRESSION VARIABLES Any_selfharm_ny /METHOD=ENTER clu# /CONTRAST (clu#)=INDICATOR(1) /PRINT=CI(95) /CRITERIA=PIN(0.05) POUT(0.10) ITERATE(20) CUT(0.5).
Resources	Processor Time	00:00:00,24
	Elapsed Time	00:00:00,00

Case Processing Summary

Unweighted Cases[a]		N	Percent
Selected Cases	Included in Analysis	5086	89.5
	Missing Cases	595	10.5
	Total	5681	100.0
Unselected Cases		0	.0
Total		5681	100.0

a. If weight is in effect, see classification table for the total number of cases.

Dependent Variable Encoding

Original Value	Internal Value
Nej	0
Ja	1

Categorical Variables Codings

			Parameter coding					
		Frequency	(1)	(2)	(3)	(4)	(5)	(6)
Cluster modal	1	2351	.000	.000	.000	.000	.000	.000
	2	1058	1.000	.000	.000	.000	.000	.000
	3	523	.000	1.000	.000	.000	.000	.000
	4	386	.000	.000	1.000	.000	.000	.000
	5	280	.000	.000	.000	1.000	.000	.000
	6	280	.000	.000	.000	.000	1.000	.000
	7	208	.000	.000	.000	.000	.000	1.000

Block 0: Beginning Block

Classification Table[a,b]

Observed			Predicted: Self-harm_ny Nej	Ja	Percentage Correct
Step 0	Self-harm_ny	Nej	4333	0	100.0
		Ja	753	0	.0
	Overall Percentage				85.2

a. Constant is included in the model.

b. The cut value is ,500

Variables in the Equation

		B	S.E.	Wald	df	Sig.	Exp(B)
Step 0	Constant	-1.750	.039	1964.530	1	.000	.174

Variables not in the Equation

			Score	df	Sig.
Step 0	Variables	Cluster modal	630.470	6	<.001
		Cluster modal(1)	3.546	1	.060
		Cluster modal(2)	1.503	1	.220
		Cluster modal(3)	93.476	1	<.001
		Cluster modal(4)	2.730	1	.098
		Cluster modal(5)	82.731	1	<.001
		Cluster modal(6)	302.219	1	<.001
	Overall Statistics		630.470	6	<.001

Block 1: Method = Enter

Omnibus Tests of Model Coefficients

		Chi-square	df	Sig.
Step 1	Step	548.403	6	<.001
	Block	548.403	6	<.001
	Model	548.403	6	<.001

Model Summary

Step	-2 Log likelihood	Cox & Snell R Square	Nagelkerke R Square
1	3716.897[a]	.102	.180

a. Estimation terminated at iteration number 6 because parameter estimates changed by less than ,001.

Classification Table[a]

Observed			Predicted Self-harm_ny Nej	Ja	Percentage Correct
Step 1	Self-harm_ny	Nej	4243	90	97.9
		Ja	635	118	15.7
	Overall Percentage				85.7

a. The cut value is ,500

Variables in the Equation

		B	S.E.	Wald	df	Sig.	Exp(B)	95% C.I.for EXP(B) Lower	Upper
Step 1[a]	Cluster modal			483.749	6	<.001			
	Cluster modal(1)	1.276	.124	106.284	1	<.001	3.584	2.812	4.568
	Cluster modal(2)	.987	.159	38.353	1	<.001	2.684	1.964	3.669
	Cluster modal(3)	2.116	.143	218.474	1	<.001	8.300	6.269	10.988
	Cluster modal(4)	1.386	.180	59.149	1	<.001	4.000	2.809	5.695
	Cluster modal(5)	2.206	.157	198.344	1	<.001	9.076	6.677	12.337
	Cluster modal(6)	3.159	.168	355.145	1	<.001	23.547	16.953	32.706
	Constant	-2.888	.092	979.766	1	<.001	.056		

a. Variable(s) entered on step 1: Cluster modal.

Logistic Regression

Notes

Output Created	26-AUG-2025 11:46:47
Comments	

Input	Data	/Users/stevenlc/Library/CloudStorage/OneDrive-Privat/ICloud filer/Doktorander/Rickard/Artikel 4/Rickardonlywomen7classmodel.sav
	Active Dataset	DataSet4
	File Label	Scored Data File
	Filter	<none>
	Weight	<none>
	Split File	<none>
	N of Rows in Working Data File	5681
Missing Value Handling	Definition of Missing	User-defined missing values are treated as missing
Syntax		LOGISTIC REGRESSION VARIABLES Symtom_score_måttlig_ny /METHOD=ENTER clu# /CONTRAST (clu#)=INDICATOR(1) /PRINT=CI(95) /CRITERIA=PIN(0.05) POUT(0.10) ITERATE(20) CUT(0.5).
Resources	Processor Time	00:00:00,23
	Elapsed Time	00:00:00,00

Case Processing Summary

Unweighted Cases[a]		N	Percent
Selected Cases	Included in Analysis	4698	82.7
	Missing Cases	983	17.3
	Total	5681	100.0
Unselected Cases		0	.0
Total		5681	100.0

a. If weight is in effect, see classification table for the total number of cases.

Dependent Variable Encoding

Original Value	Internal Value
Nej	0

Ja	1

Categorical Variables Codings

		Frequency	Parameter coding (1)	(2)	(3)	(4)	(5)	(6)
Cluster modal	1	2167	.000	.000	.000	.000	.000	.000
	2	987	1.000	.000	.000	.000	.000	.000
	3	479	.000	1.000	.000	.000	.000	.000
	4	364	.000	.000	1.000	.000	.000	.000
	5	260	.000	.000	.000	1.000	.000	.000
	6	250	.000	.000	.000	.000	1.000	.000
	7	191	.000	.000	.000	.000	.000	1.000

Block 0: Beginning Block

Classification Table[a,b]

Observed			Predicted Somatization_ny Nej	Ja	Percentage Correct
Step 0	Somatization_ny	Nej	4328	0	100.0
		Ja	370	0	.0
	Overall Percentage				92.1

a. Constant is included in the model.

b. The cut value is ,500

Variables in the Equation

		B	S.E.	Wald	df	Sig.	Exp(B)
Step 0	Constant	-2.459	.054	2061.671	1	.000	.085

Variables not in the Equation

			Score	df	Sig.
Step 0	Variables	Cluster modal	146.793	6	<.001
		Cluster modal(1)	.801	1	.371
		Cluster modal(2)	.344	1	.558
		Cluster modal(3)	7.296	1	.007
		Cluster modal(4)	6.214	1	.013
		Cluster modal(5)	26.445	1	<.001

	Cluster modal(6)	76.820	1	<.001
	Overall Statistics	146.793	6	<.001

Block 1: Method = Enter

Omnibus Tests of Model Coefficients

		Chi-square	df	Sig.
Step 1	Step	121.374	6	<.001
	Block	121.374	6	<.001
	Model	121.374	6	<.001

Model Summary

Step	-2 Log likelihood	Cox & Snell R Square	Nagelkerke R Square
1	2469.317[a]	.026	.060

a. Estimation terminated at iteration number 6 because parameter estimates changed by less than ,001.

Classification Table[a]

			Predicted		
			Somatization_ny		Percentage Correct
	Observed		Nej	Ja	
Step 1	Somatization_ny	Nej	4328	0	100.0
		Ja	370	0	.0
	Overall Percentage				92.1

a. The cut value is ,500

Variables in the Equation

		B	S.E.	Wald	df	Sig.	Exp(B)	95% C.I.for EXP(B) Lower	Upper
Step 1[a]	Cluster modal			127.830	6	<.001			
	Cluster modal(1)	.503	.161	9.753	1	.002	1.654	1.206	2.268
	Cluster modal(2)	.692	.194	12.779	1	<.001	1.998	1.367	2.919
	Cluster modal(3)	1.024	.194	27.792	1	<.001	2.784	1.902	4.073
	Cluster modal(4)	1.061	.218	23.734	1	<.001	2.889	1.885	4.427
	Cluster modal(5)	1.432	.200	51.295	1	<.001	4.186	2.829	6.195
	Cluster modal(6)	1.941	.198	96.562	1	<.001	6.965	4.729	10.258
	Constant	-3.061	.104	867.949	1	<.001	.047		

a. Variable(s) entered on step 1: Cluster modal.

Logistic Regression

Notes

Output Created		26-AUG-2025 11:46:47
Comments		
Input	Data	/Users/stevenlc/Library/CloudStorage/OneDrive-Privat/ICloud filer/Doktorander/Rickard/Artikel 4/Rickardonlywomen7classmodel.sav
	Active Dataset	DataSet4
	File Label	Scored Data File
	Filter	<none>
	Weight	<none>
	Split File	<none>
	N of Rows in Working Data File	5681
Missing Value Handling	Definition of Missing	User-defined missing values are treated as missing
Syntax		LOGISTIC REGRESSION VARIABLES IBS_ny /METHOD=ENTER clu# /CONTRAST (clu#)=INDICATOR(1) /PRINT=CI(95) /CRITERIA=PIN(0.05) POUT(0.10) ITERATE(20) CUT(0.5).
Resources	Processor Time	00:00:00,24
	Elapsed Time	00:00:01,00

Case Processing Summary

Unweighted Cases[a]		N	Percent
Selected Cases	Included in Analysis	5107	89.9
	Missing Cases	574	10.1
	Total	5681	100.0

Unselected Cases	0	.0
Total	5681	100.0

a. If weight is in effect, see classification table for the total number of cases.

Dependent Variable Encoding

Original Value	Internal Value
Nej	0
Ja	1

Categorical Variables Codings

			Parameter coding					
		Frequency	(1)	(2)	(3)	(4)	(5)	(6)
Cluster modal	1	2365	.000	.000	.000	.000	.000	.000
	2	1061	1.000	.000	.000	.000	.000	.000
	3	524	.000	1.000	.000	.000	.000	.000
	4	387	.000	.000	1.000	.000	.000	.000
	5	281	.000	.000	.000	1.000	.000	.000
	6	281	.000	.000	.000	.000	1.000	.000
	7	208	.000	.000	.000	.000	.000	1.000

Block 0: Beginning Block

Classification Table[a,b]

			Predicted		
			IBS_ny		Percentage Correct
	Observed		Nej	Ja	
Step 0	IBS_ny	Nej	4795	0	100.0
		Ja	312	0	.0
	Overall Percentage				93.9

a. Constant is included in the model.
b. The cut value is ,500

Variables in the Equation

		B	S.E.	Wald	df	Sig.	Exp(B)
Step 0	Constant	-2.732	.058	2186.967	1	.000	.065

Variables not in the Equation

			Score	df	Sig.
Step 0	Variables	Cluster modal	37.722	6	<.001
		Cluster modal(1)	.482	1	.488
		Cluster modal(2)	2.365	1	.124
		Cluster modal(3)	1.970	1	.160
		Cluster modal(4)	2.234	1	.135
		Cluster modal(5)	4.028	1	.045
		Cluster modal(6)	17.849	1	<.001
	Overall Statistics		37.722	6	<.001

Block 1: Method = Enter

Omnibus Tests of Model Coefficients

		Chi-square	df	Sig.
Step 1	Step	33.844	6	<.001
	Block	33.844	6	<.001
	Model	33.844	6	<.001

Model Summary

Step	-2 Log likelihood	Cox & Snell R Square	Nagelkerke R Square
1	2315.002[a]	.007	.018

a. Estimation terminated at iteration number 6 because parameter estimates changed by less than ,001.

Classification Table[a]

Observed			Predicted: IBS_ny Nej	Predicted: IBS_ny Ja	Predicted: Percentage Correct
Step 1	IBS_ny	Nej	4795	0	100.0
		Ja	312	0	.0
	Overall Percentage				93.9

a. The cut value is ,500

Variables in the Equation

		B	S.E.	Wald	df	Sig.	Exp(B)	95% C.I.for EXP(B) Lower	Upper
Step 1[a]	Cluster modal			35.897	6	<.001			
	Cluster modal(1)	.235	.166	2.011	1	.156	1.265	.914	1.750
	Cluster modal(2)	.556	.192	8.394	1	.004	1.744	1.197	2.541
	Cluster modal(3)	.573	.214	7.146	1	.008	1.773	1.165	2.699
	Cluster modal(4)	.632	.239	6.989	1	.008	1.881	1.178	3.006
	Cluster modal(5)	.723	.232	9.738	1	.002	2.061	1.309	3.246
	Cluster modal(6)	1.147	.229	25.120	1	<.001	3.148	2.010	4.929
	Constant	-3.049	.099	949.964	1	<.001	.047		

a. Variable(s) entered on step 1: Cluster modal.

Logistic Regression

Notes

Output Created		26-AUG-2025 11:46:48
Comments		
Input	Data	/Users/stevenlc/Library/CloudStorage/OneDrive-Privat/ICloud filer/Doktorander/Rickard/Artikel 4/Rickardonlywomen7classmodel.sav
	Active Dataset	DataSet4
	File Label	Scored Data File
	Filter	<none>
	Weight	<none>
	Split File	<none>
	N of Rows in Working Data File	5681
Missing Value Handling	Definition of Missing	User-defined missing values are treated as missing

Syntax		LOGISTIC REGRESSION VARIABLES Fibromyalgi_ny<br>/METHOD=ENTER clu#<br>/CONTRAST (clu#)=INDICATOR(1)<br>/PRINT=CI(95)<br>/CRITERIA=PIN(0.05) POUT(0.10) ITERATE(20) CUT(0.5).
Resources	Processor Time	00:00:00,23
	Elapsed Time	00:00:00,00

Case Processing Summary

Unweighted Cases[a]		N	Percent
Selected Cases	Included in Analysis	5107	89.9
	Missing Cases	574	10.1
	Total	5681	100.0
Unselected Cases		0	.0
Total		5681	100.0

a. If weight is in effect, see classification table for the total number of cases.

Dependent Variable Encoding

Original Value	Internal Value
Nej	0
Ja	1

Categorical Variables Codings

		Frequency	Parameter coding					
			(1)	(2)	(3)	(4)	(5)	(6)
Cluster modal	1	2365	.000	.000	.000	.000	.000	.000
	2	1061	1.000	.000	.000	.000	.000	.000
	3	524	.000	1.000	.000	.000	.000	.000
	4	387	.000	.000	1.000	.000	.000	.000
	5	281	.000	.000	.000	1.000	.000	.000
	6	281	.000	.000	.000	.000	1.000	.000
	7	208	.000	.000	.000	.000	.000	1.000

Block 0: Beginning Block

Classification Table[a,b]

Observed			Predicted: Fibromyalgia_ny Nej	Predicted: Fibromyalgia_ny Ja	Predicted: Percentage Correct
Step 0	Fibromyalgia_ny	Nej	4944	0	100.0
		Ja	163	0	.0
	Overall Percentage				96.8

a. Constant is included in the model.

b. The cut value is ,500

Variables in the Equation

		B	S.E.	Wald	df	Sig.	Exp(B)
Step 0	Constant	-3.412	.080	1837.232	1	.000	.033

Variables not in the Equation

			Score	df	Sig.
Step 0	Variables	Cluster modal	32.365	6	<.001
		Cluster modal(1)	2.381	1	.123
		Cluster modal(2)	.511	1	.475
		Cluster modal(3)	.635	1	.426
		Cluster modal(4)	6.026	1	.014
		Cluster modal(5)	12.265	1	<.001
		Cluster modal(6)	8.790	1	.003
	Overall Statistics		32.365	6	<.001

Block 1: Method = Enter

Omnibus Tests of Model Coefficients

		Chi-square	df	Sig.
Step 1	Step	27.018	6	<.001
	Block	27.018	6	<.001
	Model	27.018	6	<.001

Model Summary

Step	-2 Log likelihood	Cox & Snell R Square	Nagelkerke R Square
1	1416.668[a]	.005	.021

a. Estimation terminated at iteration number 6 because parameter estimates changed by less than ,001.

Classification Table[a]

	Observed		Predicted Fibromyalgia_ny Nej	Predicted Fibromyalgia_ny Ja	Predicted Percentage Correct
Step 1	Fibromyalgia_ny	Nej	4944	0	100.0
		Ja	163	0	.0
	Overall Percentage				96.8

a. The cut value is ,500

Variables in the Equation

		B	S.E.	Wald	df	Sig.	Exp(B)	95% C.I.for EXP(B) Lower	95% C.I.for EXP(B) Upper
Step 1[a]	Cluster modal			30.203	6	<.001			
	Cluster modal(1)	-.018	.238	.006	1	.939	.982	.615	1.566
	Cluster modal(2)	.070	.301	.055	1	.815	1.073	.594	1.937
	Cluster modal(3)	.455	.295	2.386	1	.122	1.576	.885	2.807
	Cluster modal(4)	.859	.289	8.812	1	.003	2.360	1.339	4.160
	Cluster modal(5)	1.042	.272	14.701	1	<.001	2.834	1.664	4.828
	Cluster modal(6)	1.037	.307	11.443	1	<.001	2.821	1.547	5.144
	Constant	-3.666	.132	773.039	1	<.001	.026		

a. Variable(s) entered on step 1: Cluster modal.

Logistic Regression

Notes

Output Created	26-AUG-2025 11:46:48
Comments	

Input	Data	/Users/stevenlc/Library/CloudStorage/OneDrive-Privat/ICloud filer/Doktorander/Rickard/Artikel 4/Rickardonlywomen7classmodel.sav
	Active Dataset	DataSet4
	File Label	Scored Data File
	Filter	<none>
	Weight	<none>
	Split File	<none>
	N of Rows in Working Data File	5681
Missing Value Handling	Definition of Missing	User-defined missing values are treated as missing
Syntax		LOGISTIC REGRESSION VARIABLES Ischemic_HD_ny /METHOD=ENTER clu# /CONTRAST (clu#)=INDICATOR(1) /PRINT=CI(95) /CRITERIA=PIN(0.05) POUT(0.10) ITERATE(20) CUT(0.5).
Resources	Processor Time	00:00:00,24
	Elapsed Time	00:00:00,00

Case Processing Summary

Unweighted Cases[a]		N	Percent
Selected Cases	Included in Analysis	5107	89.9
	Missing Cases	574	10.1
	Total	5681	100.0
Unselected Cases		0	.0
Total		5681	100.0

a. If weight is in effect, see classification table for the total number of cases.

Dependent Variable Encoding

Original Value	Internal Value
Nej	0

Ja	1

Categorical Variables Codings

		Frequency	Parameter coding (1)	(2)	(3)	(4)	(5)	(6)
Cluster modal	1	2365	.000	.000	.000	.000	.000	.000
	2	1061	1.000	.000	.000	.000	.000	.000
	3	524	.000	1.000	.000	.000	.000	.000
	4	387	.000	.000	1.000	.000	.000	.000
	5	281	.000	.000	.000	1.000	.000	.000
	6	281	.000	.000	.000	.000	1.000	.000
	7	208	.000	.000	.000	.000	.000	1.000

Block 0: Beginning Block

Classification Table[a,b]

Observed			Predicted IHD_ny Nej	Ja	Percentage Correct
Step 0	IHD_ny	Nej	4949	0	100.0
		Ja	158	0	.0
	Overall Percentage				96.9

a. Constant is included in the model.

b. The cut value is ,500

Variables in the Equation

		B	S.E.	Wald	df	Sig.	Exp(B)
Step 0	Constant	-3.444	.081	1816.445	1	.000	.032

Variables not in the Equation

			Score	df	Sig.
Step 0	Variables	Cluster modal	19.349	6	.004
		Cluster modal(1)	5.549	1	.018
		Cluster modal(2)	1.926	1	.165
		Cluster modal(3)	3.387	1	.066
		Cluster modal(4)	4.072	1	.044
		Cluster modal(5)	3.537	1	.060

Cluster modal(6)	2.124	1	.145
Overall Statistics	19.349	6	.004

Block 1: Method = Enter

Omnibus Tests of Model Coefficients

		Chi-square	df	Sig.
Step 1	Step	20.232	6	.003
	Block	20.232	6	.003
	Model	20.232	6	.003

Model Summary

Step	-2 Log likelihood	Cox & Snell R Square	Nagelkerke R Square
1	1389.173[a]	.004	.016

a. Estimation terminated at iteration number 7 because parameter estimates changed by less than ,001.

Classification Table[a]

			Predicted		
			IHD_ny		Percentage Correct
Observed			Nej	Ja	
Step 1	IHD_ny	Nej	4949	0	100.0
		Ja	158	0	.0
	Overall Percentage				96.9

a. The cut value is ,500

Variables in the Equation

		B	S.E.	Wald	df	Sig.	Exp(B)	95% C.I.for EXP(B) Lower	Upper
Step 1[a]	Cluster modal			18.148	6	.006			
	Cluster modal(1)	-.563	.248	5.169	1	.023	.569	.350	.925
	Cluster modal(2)	-.503	.325	2.396	1	.122	.605	.320	1.143
	Cluster modal(3)	.319	.267	1.431	1	.232	1.375	.816	2.319
	Cluster modal(4)	-1.190	.591	4.048	1	.044	.304	.095	.970
	Cluster modal(5)	.391	.297	1.739	1	.187	1.479	.827	2.644
	Cluster modal(6)	.354	.343	1.061	1	.303	1.424	.727	2.791
	Constant	-3.339	.113	872.256	1	<.001	.035		

a. Variable(s) entered on step 1: Cluster modal.

Logistic Regression

Notes

Output Created		26-AUG-2025 11:46:48
Comments		
Input	Data	/Users/stevenlc/Library/CloudStorage/OneDrive-Privat/ICloud filer/Doktorander/Rickard/Artikel 4/Rickardonlywomen7classmodel.sav
	Active Dataset	DataSet4
	File Label	Scored Data File
	Filter	<none>
	Weight	<none>
	Split File	<none>
	N of Rows in Working Data File	5681
Missing Value Handling	Definition of Missing	User-defined missing values are treated as missing
Syntax		LOGISTIC REGRESSION VARIABLES KOL_ny /METHOD=ENTER clu# /CONTRAST (clu#)=INDICATOR(1) /PRINT=CI(95) /CRITERIA=PIN(0.05) POUT(0.10) ITERATE(20) CUT(0.5).
Resources	Processor Time	00:00:00,24
	Elapsed Time	00:00:00,00

Case Processing Summary

Unweighted Cases[a]		N	Percent
Selected Cases	Included in Analysis	5107	89.9
	Missing Cases	574	10.1
	Total	5681	100.0

Unselected Cases	0	.0
Total	5681	100.0

a. If weight is in effect, see classification table for the total number of cases.

Dependent Variable Encoding

Original Value	Internal Value
Nej	0
Ja	1

Categorical Variables Codings

			Parameter coding					
		Frequency	(1)	(2)	(3)	(4)	(5)	(6)
Cluster modal	1	2365	.000	.000	.000	.000	.000	.000
	2	1061	1.000	.000	.000	.000	.000	.000
	3	524	.000	1.000	.000	.000	.000	.000
	4	387	.000	.000	1.000	.000	.000	.000
	5	281	.000	.000	.000	1.000	.000	.000
	6	281	.000	.000	.000	.000	1.000	.000
	7	208	.000	.000	.000	.000	.000	1.000

Block 0: Beginning Block

Classification Table[a,b]

			Predicted		
			COPD_ny		Percentage Correct
Observed			Nej	Ja	
Step 0	COPD_ny	Nej	5021	0	100.0
		Ja	86	0	.0
	Overall Percentage				98.3

a. Constant is included in the model.
b. The cut value is ,500

Variables in the Equation

		B	S.E.	Wald	df	Sig.	Exp(B)
Step 0	Constant	-4.067	.109	1398.553	1	<.001	.017

Variables not in the Equation

			Score	df	Sig.
Step 0	Variables	Cluster modal	22.458	6	<.001
		Cluster modal(1)	1.702	1	.192
		Cluster modal(2)	.087	1	.768
		Cluster modal(3)	2.089	1	.148
		Cluster modal(4)	8.937	1	.003
		Cluster modal(5)	4.144	1	.042
		Cluster modal(6)	6.123	1	.013
	Overall Statistics		22.458	6	<.001

Block 1: Method = Enter

Omnibus Tests of Model Coefficients

		Chi-square	df	Sig.
Step 1	Step	18.480	6	.005
	Block	18.480	6	.005
	Model	18.480	6	.005

Model Summary

Step	-2 Log likelihood	Cox & Snell R Square	Nagelkerke R Square
1	854.515[a]	.004	.023

a. Estimation terminated at iteration number 8 because parameter estimates changed by less than ,001.

Classification Table[a]

	Observed		Predicted COPD_ny Nej	Predicted COPD_ny Ja	Predicted Percentage Correct
Step 1	COPD_ny	Nej	5021	0	100.0
		Ja	86	0	.0
	Overall Percentage				98.3

a. The cut value is ,500

Variables in the Equation

		B	S.E.	Wald	df	Sig.	Exp(B)	95% C.I.for EXP(B) Lower	Upper
Step 1[a]	Cluster modal			20.447	6	.002			
	Cluster modal(1)	-.162	.328	.244	1	.622	.850	.447	1.618
	Cluster modal(2)	.061	.396	.024	1	.878	1.063	.489	2.310
	Cluster modal(3)	-.624	.605	1.066	1	.302	.536	.164	1.752
	Cluster modal(4)	1.027	.353	8.478	1	.004	2.793	1.399	5.577
	Cluster modal(5)	.819	.380	4.639	1	.031	2.268	1.077	4.780
	Cluster modal(6)	1.009	.400	6.367	1	.012	2.742	1.253	6.004
	Constant	-4.228	.173	598.959	1	<.001	.015		

a. Variable(s) entered on step 1: Cluster modal.

Logistic Regression

Notes

Output Created		26-AUG-2025 11:46:48
Comments		
Input	Data	/Users/stevenlc/Library/CloudStorage/OneDrive-Privat/ICloud filer/Doktorander/Rickard/Artikel 4/Rickardonlywomen7classmodel.sav
	Active Dataset	DataSet4
	File Label	Scored Data File
	Filter	<none>
	Weight	<none>
	Split File	<none>
	N of Rows in Working Data File	5681
Missing Value Handling	Definition of Missing	User-defined missing values are treated as missing

Syntax		LOGISTIC REGRESSION VARIABLES Diabetes_II_ny<br>/METHOD=ENTER clu#<br>/CONTRAST (clu#)=INDICATOR(1)<br>/PRINT=CI(95)<br>/CRITERIA=PIN(0.05) POUT(0.10) ITERATE(20) CUT(0.5).
Resources	Processor Time	00:00:00,24
	Elapsed Time	00:00:01,00

Case Processing Summary

Unweighted Cases[a]		N	Percent
Selected Cases	Included in Analysis	5107	89.9
	Missing Cases	574	10.1
	Total	5681	100.0
Unselected Cases		0	.0
Total		5681	100.0

a. If weight is in effect, see classification table for the total number of cases.

Dependent Variable Encoding

Original Value	Internal Value
Nej	0
Ja	1

Categorical Variables Codings

			Parameter coding					
		Frequency	(1)	(2)	(3)	(4)	(5)	(6)
Cluster modal	1	2365	.000	.000	.000	.000	.000	.000
	2	1061	1.000	.000	.000	.000	.000	.000
	3	524	.000	1.000	.000	.000	.000	.000
	4	387	.000	.000	1.000	.000	.000	.000
	5	281	.000	.000	.000	1.000	.000	.000
	6	281	.000	.000	.000	.000	1.000	.000
	7	208	.000	.000	.000	.000	.000	1.000

Block 0: Beginning Block

Classification Table[a,b]

Observed			Predicted: Diabetes type II_ny Nej	Predicted: Diabetes type II_ny Ja	Predicted: Percentage Correct
Step 0	Diabetes type II_ny	Nej	4977	0	100.0
		Ja	130	0	.0
	Overall Percentage				97.5

a. Constant is included in the model.
b. The cut value is ,500

Variables in the Equation

		B	S.E.	Wald	df	Sig.	Exp(B)
Step 0	Constant	-3.645	.089	1683.262	1	.000	.026

Variables not in the Equation

			Score	df	Sig.
Step 0	Variables	Cluster modal	21.152	6	.002
		Cluster modal(1)	6.915	1	.009
		Cluster modal(2)	3.444	1	.063
		Cluster modal(3)	.916	1	.338
		Cluster modal(4)	1.231	1	.267
		Cluster modal(5)	2.618	1	.106
		Cluster modal(6)	1.479	1	.224
	Overall Statistics		21.152	6	.002

Block 1: Method = Enter

Omnibus Tests of Model Coefficients

		Chi-square	df	Sig.
Step 1	Step	22.669	6	<.001
	Block	22.669	6	<.001
	Model	22.669	6	<.001

Model Summary

Step	-2 Log likelihood	Cox & Snell R Square	Nagelkerke R Square
1	1188.410[a]	.004	.021

a. Estimation terminated at iteration number 7 because parameter estimates changed by less than ,001.

Classification Table[a]

Observed			Predicted: Diabetes type II_ny Nej	Ja	Percentage Correct
Step 1	Diabetes type II_ny	Nej	4977	0	100.0
		Ja	130	0	.0
	Overall Percentage				97.5

a. The cut value is ,500

Variables in the Equation

		B	S.E.	Wald	df	Sig.	Exp(B)	95% C.I.for EXP(B) Lower	Upper
Step 1[a]	Cluster modal			19.710	6	.003			
	Cluster modal(1)	-.893	.284	9.890	1	.002	.410	.235	.714
	Cluster modal(2)	-.950	.397	5.722	1	.017	.387	.178	.842
	Cluster modal(3)	-.642	.398	2.603	1	.107	.526	.241	1.148
	Cluster modal(4)	.053	.342	.024	1	.878	1.054	.540	2.058
	Cluster modal(5)	-1.177	.591	3.959	1	.047	.308	.097	.983
	Cluster modal(6)	.133	.378	.124	1	.725	1.142	.545	2.397
	Constant	-3.352	.114	868.516	1	<.001	.035		

a. Variable(s) entered on step 1: Cluster modal.

Logistic Regression

Notes

Output Created	26-AUG-2025 11:46:49
Comments	

Input	Data	/Users/stevenlc/Library/CloudStorage/OneDrive-Privat/ICloud filer/Doktorander/Rickard/Artikel 4/Rickardonlywomen7classmodel.sav
	Active Dataset	DataSet4
	File Label	Scored Data File
	Filter	<none>
	Weight	<none>
	Split File	<none>
	N of Rows in Working Data File	5681
Missing Value Handling	Definition of Missing	User-defined missing values are treated as missing
Syntax		LOGISTIC REGRESSION VARIABLES Tumörsjd_ny /METHOD=ENTER clu# /CONTRAST (clu#)=INDICATOR(1) /PRINT=CI(95) /CRITERIA=PIN(0.05) POUT(0.10) ITERATE(20) CUT(0.5).
Resources	Processor Time	00:00:00,24
	Elapsed Time	00:00:00,00

Case Processing Summary

Unweighted Cases[a]		N	Percent
Selected Cases	Included in Analysis	5107	89.9
	Missing Cases	574	10.1
	Total	5681	100.0
Unselected Cases		0	.0
Total		5681	100.0

a. If weight is in effect, see classification table for the total number of cases.

Dependent Variable Encoding

Original Value	Internal Value
Nej	0
Ja	1

Categorical Variables Codings

		Frequency	Parameter coding (1)	(2)	(3)	(4)	(5)	(6)
Cluster modal	1	2365	.000	.000	.000	.000	.000	.000
	2	1061	1.000	.000	.000	.000	.000	.000
	3	524	.000	1.000	.000	.000	.000	.000
	4	387	.000	.000	1.000	.000	.000	.000
	5	281	.000	.000	.000	1.000	.000	.000
	6	281	.000	.000	.000	.000	1.000	.000
	7	208	.000	.000	.000	.000	.000	1.000

Block 0: Beginning Block

Classification Table[a,b]

Observed			Predicted Cancer_ny Nej	Ja	Percentage Correct
Step 0	Cancer_ny	Nej	4861	0	100.0
		Ja	246	0	.0
	Overall Percentage				95.2

a. Constant is included in the model.

b. The cut value is ,500

Variables in the Equation

		B	S.E.	Wald	df	Sig.	Exp(B)
Step 0	Constant	-2.984	.065	2084.471	1	.000	.051

Variables not in the Equation

			Score	df	Sig.
Step 0	Variables	Cluster modal	2.525	6	.866
		Cluster modal(1)	.438	1	.508
		Cluster modal(2)	.027	1	.870
		Cluster modal(3)	.008	1	.929
		Cluster modal(4)	.528	1	.467
		Cluster modal(5)	1.637	1	.201
		Cluster modal(6)	.105	1	.746

	Overall Statistics	2.525	6	.866

Block 1: Method = Enter

Omnibus Tests of Model Coefficients

		Chi-square	df	Sig.
Step 1	Step	2.425	6	.877
	Block	2.425	6	.877
	Model	2.425	6	.877

Model Summary

Step	-2 Log likelihood	Cox & Snell R Square	Nagelkerke R Square
1	1969.784[a]	.000	.001

a. Estimation terminated at iteration number 6 because parameter estimates changed by less than ,001.

Classification Table[a]

Observed			Predicted Cancer_ny Nej	Ja	Percentage Correct
Step 1	Cancer_ny	Nej	4861	0	100.0
		Ja	246	0	.0
	Overall Percentage				95.2

a. The cut value is ,500

Variables in the Equation

		B	S.E.	Wald	df	Sig.	Exp(B)	95% C.I.for EXP(B) Lower	Upper
Step 1[a]	Cluster modal			2.506	6	.868			
	Cluster modal(1)	-.089	.177	.249	1	.618	.915	.646	1.296
	Cluster modal(2)	.030	.223	.019	1	.891	1.031	.666	1.596
	Cluster modal(3)	.019	.254	.006	1	.939	1.019	.620	1.678
	Cluster modal(4)	-.218	.322	.456	1	.499	.804	.428	1.513
	Cluster modal(5)	.301	.262	1.323	1	.250	1.351	.809	2.258
	Cluster modal(6)	.098	.324	.091	1	.763	1.103	.584	2.082
	Constant	-2.983	.096	965.463	1	<.001	.051		

a. Variable(s) entered on step 1: Cluster modal.

Logistic Regression

Notes

Output Created		26-AUG-2025 11:46:49
Comments		
Input	Data	/Users/stevenlc/Library/CloudStorage/OneDrive-Privat/ICloud filer/Doktorander/Rickard/Artikel 4/Rickardonlywomen7classmodel.sav
	Active Dataset	DataSet4
	File Label	Scored Data File
	Filter	<none>
	Weight	<none>
	Split File	<none>
	N of Rows in Working Data File	5681
Missing Value Handling	Definition of Missing	User-defined missing values are treated as missing
Syntax		LOGISTIC REGRESSION VARIABLES Fetma_ny /METHOD=ENTER clu# /CONTRAST (clu#)=INDICATOR(1) /PRINT=CI(95) /CRITERIA=PIN(0.05) POUT(0.10) ITERATE(20) CUT(0.5).
Resources	Processor Time	00:00:00,23
	Elapsed Time	00:00:00,00

Case Processing Summary

Unweighted Cases[a]		N	Percent
Selected Cases	Included in Analysis	5002	88.0
	Missing Cases	679	12.0
	Total	5681	100.0
Unselected Cases		0	.0

Total	5681	100.0

a. If weight is in effect, see classification table for the total number of cases.

Dependent Variable Encoding

Original Value	Internal Value
Nej	0
Ja	1

Categorical Variables Codings

			Parameter coding					
		Frequency	(1)	(2)	(3)	(4)	(5)	(6)
Cluster modal	1	2314	.000	.000	.000	.000	.000	.000
	2	1038	1.000	.000	.000	.000	.000	.000
	3	515	.000	1.000	.000	.000	.000	.000
	4	379	.000	.000	1.000	.000	.000	.000
	5	276	.000	.000	.000	1.000	.000	.000
	6	278	.000	.000	.000	.000	1.000	.000
	7	202	.000	.000	.000	.000	.000	1.000

Block 0: Beginning Block

Classification Table[a,b]

			Predicted		
			Obesity_ny		Percentage Correct
Observed			Nej	Ja	
Step 0	Obesity_ny	Nej	4369	0	100.0
		Ja	633	0	.0
	Overall Percentage				87.3

a. Constant is included in the model.

b. The cut value is ,500

Variables in the Equation

		B	S.E.	Wald	df	Sig.	Exp(B)
Step 0	Constant	-1.932	.043	2063.360	1	.000	.145

Variables not in the Equation

			Score	df	Sig.
Step 0	Variables	Cluster modal	19.873	6	.003
		Cluster modal(1)	6.525	1	.011
		Cluster modal(2)	2.027	1	.155
		Cluster modal(3)	4.390	1	.036
		Cluster modal(4)	.297	1	.586
		Cluster modal(5)	1.602	1	.206
		Cluster modal(6)	7.219	1	.007
	Overall Statistics		19.873	6	.003

Block 1: Method = Enter

Omnibus Tests of Model Coefficients

		Chi-square	df	Sig.
Step 1	Step	19.143	6	.004
	Block	19.143	6	.004
	Model	19.143	6	.004

Model Summary

Step	-2 Log likelihood	Cox & Snell R Square	Nagelkerke R Square
1	3780.118[a]	.004	.007

a. Estimation terminated at iteration number 5 because parameter estimates changed by less than ,001.

Classification Table[a]

Observed			Predicted: Obesity_ny Nej	Predicted: Obesity_ny Ja	Predicted: Percentage Correct
Step 1	Obesity_ny	Nej	4369	0	100.0
		Ja	633	0	.0
	Overall Percentage				87.3

a. The cut value is ,500

Variables in the Equation

		B	S.E.	Wald	df	Sig.	Exp(B)	95% C.I.for EXP(B) Lower	Upper
Step 1[a]	Cluster modal			19.600	6	.003			
	Cluster modal(1)	-.252	.119	4.437	1	.035	.778	.615	.983
	Cluster modal(2)	-.212	.156	1.859	1	.173	.809	.596	1.097
	Cluster modal(3)	.261	.153	2.903	1	.088	1.298	.962	1.751
	Cluster modal(4)	-.120	.198	.365	1	.546	.887	.602	1.308
	Cluster modal(5)	.186	.179	1.080	1	.299	1.204	.848	1.709
	Cluster modal(6)	.449	.190	5.571	1	.018	1.568	1.079	2.277
	Constant	-1.912	.062	948.895	1	<.001	.148		

a. Variable(s) entered on step 1: Cluster modal.

Logistic Regression

Notes

Output Created		26-AUG-2025 11:46:49
Comments		
Input	Data	/Users/stevenlc/Library/CloudStorage/OneDrive-Privat/ICloud filer/Doktorander/Rickard/Artikel 4/Rickardonlywomen7classmodel.sav
	Active Dataset	DataSet4
	File Label	Scored Data File
	Filter	<none>
	Weight	<none>
	Split File	<none>
	N of Rows in Working Data File	5681
Missing Value Handling	Definition of Missing	User-defined missing values are treated as missing

Syntax		LOGISTIC REGRESSION VARIABLES Storrökare_ny<br>/METHOD=ENTER clu#<br>/CONTRAST (clu#)=INDICATOR(1)<br>/PRINT=CI(95)<br>/CRITERIA=PIN(0.05) POUT(0.10) ITERATE(20) CUT(0.5).
Resources	Processor Time	00:00:00,24
	Elapsed Time	00:00:00,00

Case Processing Summary

Unweighted Cases[a]		N	Percent
Selected Cases	Included in Analysis	5048	88.9
	Missing Cases	633	11.1
	Total	5681	100.0
Unselected Cases		0	.0
Total		5681	100.0

a. If weight is in effect, see classification table for the total number of cases.

Dependent Variable Encoding

Original Value	Internal Value
Nej	0
Ja	1

Categorical Variables Codings

			Parameter coding					
		Frequency	(1)	(2)	(3)	(4)	(5)	(6)
Cluster modal	1	2343	.000	.000	.000	.000	.000	.000
	2	1050	1.000	.000	.000	.000	.000	.000
	3	517	.000	1.000	.000	.000	.000	.000
	4	384	.000	.000	1.000	.000	.000	.000
	5	277	.000	.000	.000	1.000	.000	.000
	6	276	.000	.000	.000	.000	1.000	.000
	7	201	.000	.000	.000	.000	.000	1.000

Block 0: Beginning Block

Classification Table[a,b]

Observed			Predicted: Heavy smoking_ny Nej	Predicted: Heavy smoking_ny Ja	Predicted: Percentage Correct
Step 0	Heavy smoking_ny	Nej	4907	0	100.0
		Ja	141	0	.0
	Overall Percentage				97.2

a. Constant is included in the model.

b. The cut value is ,500

Variables in the Equation

		B	S.E.	Wald	df	Sig.	Exp(B)
Step 0	Constant	-3.550	.085	1726.986	1	.000	.029

Variables not in the Equation

			Score	df	Sig.
Step 0	Variables	Cluster modal	36.537	6	<.001
		Cluster modal(1)	4.725	1	.030
		Cluster modal(2)	.193	1	.660
		Cluster modal(3)	8.929	1	.003
		Cluster modal(4)	3.897	1	.048
		Cluster modal(5)	2.599	1	.107
		Cluster modal(6)	13.419	1	<.001
	Overall Statistics		36.537	6	<.001

Block 1: Method = Enter

Omnibus Tests of Model Coefficients

		Chi-square	df	Sig.
Step 1	Step	31.313	6	<.001
	Block	31.313	6	<.001
	Model	31.313	6	<.001

Model Summary

Step	-2 Log likelihood	Cox & Snell R Square	Nagelkerke R Square
1	1255.704[a]	.006	.027

a. Estimation terminated at iteration number 7 because parameter estimates changed by less than ,001.

Classification Table[a]

Observed			Predicted: Heavy smoking_ny Nej	Predicted: Heavy smoking_ny Ja	Predicted: Percentage Correct
Step 1	Heavy smoking_ny	Nej	4907	0	100.0
		Ja	141	0	.0
	Overall Percentage				97.2

a. The cut value is ,500

Variables in the Equation

		B	S.E.	Wald	df	Sig.	Exp(B)	95% C.I.for EXP(B) Lower	95% C.I.for EXP(B) Upper
Step 1[a]	Cluster modal			33.539	6	<.001			
	Cluster modal(1)	-.105	.274	.147	1	.702	.900	.526	1.542
	Cluster modal(2)	.445	.294	2.295	1	.130	1.560	.877	2.774
	Cluster modal(3)	.987	.273	13.093	1	<.001	2.684	1.572	4.582
	Cluster modal(4)	.878	.320	7.523	1	.006	2.406	1.285	4.504
	Cluster modal(5)	.798	.330	5.847	1	.016	2.221	1.163	4.239
	Cluster modal(6)	1.297	.314	17.073	1	<.001	3.657	1.977	6.765
	Constant	-3.889	.147	696.504	1	<.001	.020		

a. Variable(s) entered on step 1: Cluster modal.

Logistic Regression

Notes

Output Created	26-AUG-2025 11:46:49
Comments	

Input	Data	/Users/stevenlc/Library/CloudStorage/OneDrive-Privat/ICloud filer/Doktorander/Rickard/Artikel 4/Rickardonlywomen7classmodel.sav
	Active Dataset	DataSet4
	File Label	Scored Data File
	Filter	<none>
	Weight	<none>
	Split File	<none>
	N of Rows in Working Data File	5681
Missing Value Handling	Definition of Missing	User-defined missing values are treated as missing
Syntax		LOGISTIC REGRESSION VARIABLES AUDITRISK_inknykt_ny /METHOD=ENTER clu# /CONTRAST (clu#)=INDICATOR(1) /PRINT=CI(95) /CRITERIA=PIN(0.05) POUT(0.10) ITERATE(20) CUT(0.5).
Resources	Processor Time	00:00:00,23
	Elapsed Time	00:00:00,00

Case Processing Summary

Unweighted Cases[a]		N	Percent
Selected Cases	Included in Analysis	4950	87.1
	Missing Cases	731	12.9
	Total	5681	100.0
Unselected Cases		0	.0
Total		5681	100.0

a. If weight is in effect, see classification table for the total number of cases.

Dependent Variable Encoding

Original Value	Internal Value
Nej	0

Ja	1

Categorical Variables Codings

		Frequency	Parameter coding (1)	(2)	(3)	(4)	(5)	(6)
Cluster modal	1	2295	.000	.000	.000	.000	.000	.000
	2	1028	1.000	.000	.000	.000	.000	.000
	3	511	.000	1.000	.000	.000	.000	.000
	4	374	.000	.000	1.000	.000	.000	.000
	5	272	.000	.000	.000	1.000	.000	.000
	6	269	.000	.000	.000	.000	1.000	.000
	7	201	.000	.000	.000	.000	.000	1.000

Block 0: Beginning Block

Classification Table[a,b]

Observed			Predicted: Heavy drinking_ny Nej	Ja	Percentage Correct
Step 0	Heavy drinking_ny	Nej	4098	0	100.0
		Ja	852	0	.0
	Overall Percentage				82.8

a. Constant is included in the model.
b. The cut value is ,500

Variables in the Equation

		B	S.E.	Wald	df	Sig.	Exp(B)
Step 0	Constant	-1.571	.038	1740.103	1	.000	.208

Variables not in the Equation

			Score	df	Sig.
Step 0	Variables	Cluster modal	121.511	6	<.001
		Cluster modal(1)	13.145	1	<.001
		Cluster modal(2)	.134	1	.715
		Cluster modal(3)	7.819	1	.005
		Cluster modal(4)	2.302	1	.129
		Cluster modal(5)	12.990	1	<.001

	Cluster modal(6)	45.614	1	<.001
Overall Statistics		121.511	6	<.001

Block 1: Method = Enter

Omnibus Tests of Model Coefficients

		Chi-square	df	Sig.
Step 1	Step	115.824	6	<.001
	Block	115.824	6	<.001
	Model	115.824	6	<.001

Model Summary

Step	-2 Log likelihood	Cox & Snell R Square	Nagelkerke R Square
1	4430.590[a]	.023	.038

a. Estimation terminated at iteration number 5 because parameter estimates changed by less than ,001.

Classification Table[a]

Observed			Predicted: Heavy drinking_ny Nej	Predicted: Heavy drinking_ny Ja	Predicted: Percentage Correct
Step 1	Heavy drinking_ny	Nej	4098	0	100.0
		Ja	852	0	.0
	Overall Percentage				82.8

a. The cut value is ,500

Variables in the Equation

		B	S.E.	Wald	df	Sig.	Exp(B)	95% C.I.for EXP(B) Lower	95% C.I.for EXP(B) Upper
Step 1[a]	Cluster modal			115.602	6	<.001			
	Cluster modal(1)	.678	.100	45.903	1	<.001	1.970	1.619	2.397
	Cluster modal(2)	.391	.135	8.350	1	.004	1.478	1.134	1.926
	Cluster modal(3)	.763	.140	29.863	1	<.001	2.145	1.632	2.821
	Cluster modal(4)	.652	.163	15.976	1	<.001	1.920	1.394	2.644
	Cluster modal(5)	.919	.154	35.395	1	<.001	2.506	1.851	3.391
	Cluster modal(6)	1.376	.161	72.573	1	<.001	3.958	2.884	5.431
	Constant	-2.002	.064	964.384	1	<.001	.135		

a. Variable(s) entered on step 1: Cluster modal.

Logistic Regression

Notes

Output Created		26-AUG-2025 11:46:49
Comments		
Input	Data	/Users/stevenlc/Library/CloudStorage/OneDrive-Privat/ICloud filer/Doktorander/Rickard/Artikel 4/Rickardonlywomen7classmodel.sav
	Active Dataset	DataSet4
	File Label	Scored Data File
	Filter	<none>
	Weight	<none>
	Split File	<none>
	N of Rows in Working Data File	5681
Missing Value Handling	Definition of Missing	User-defined missing values are treated as missing
Syntax		LOGISTIC REGRESSION VARIABLES Drogmissbruk_ny<br>/METHOD=ENTER clu#<br>/CONTRAST (clu#)=INDICATOR(1)<br>/PRINT=CI(95)<br>/CRITERIA=PIN(0.05) POUT(0.10) ITERATE(20) CUT(0.5).
Resources	Processor Time	00:00:00,26
	Elapsed Time	00:00:01,00

Case Processing Summary

Unweighted Cases[a]		N	Percent
Selected Cases	Included in Analysis	5107	89.9
	Missing Cases	574	10.1

	Total	5681	100.0
Unselected Cases		0	.0
Total		5681	100.0

a. If weight is in effect, see classification table for the total number of cases.

Dependent Variable Encoding

Original Value	Internal Value
Nej	0
Ja	1

Categorical Variables Codings

		Frequency	Parameter coding (1)	(2)	(3)	(4)	(5)	(6)
Cluster modal	1	2365	.000	.000	.000	.000	.000	.000
	2	1061	1.000	.000	.000	.000	.000	.000
	3	524	.000	1.000	.000	.000	.000	.000
	4	387	.000	.000	1.000	.000	.000	.000
	5	281	.000	.000	.000	1.000	.000	.000
	6	281	.000	.000	.000	.000	1.000	.000
	7	208	.000	.000	.000	.000	.000	1.000

Block 0: Beginning Block

Classification Table[a,b]

	Observed		Predicted: Drug abuse_ny: Nej	Ja	Percentage Correct
Step 0	Drug abuse_ny	Nej	5091	0	100.0
		Ja	16	0	.0
	Overall Percentage				99.7

a. Constant is included in the model.

b. The cut value is ,500

Variables in the Equation

		B	S.E.	Wald	df	Sig.	Exp(B)
Step 0	Constant	-5.763	.250	529.664	1	<.001	.003

Variables not in the Equation

			Score	df	Sig.
Step 0	Variables	Cluster modal	38.591	6	<.001
		Cluster modal(1)	.040	1	.841
		Cluster modal(2)	1.835	1	.176
		Cluster modal(3)	.040	1	.841
		Cluster modal(4)	5.418	1	.020
		Cluster modal(5)	.017	1	.895
		Cluster modal(6)	30.343	1	<.001
	Overall Statistics		38.591	6	<.001

Block 1: Method = Enter

Omnibus Tests of Model Coefficients

		Chi-square	df	Sig.
Step 1	Step	21.684	6	.001
	Block	21.684	6	.001
	Model	21.684	6	.001

Model Summary

Step	-2 Log likelihood	Cox & Snell R Square	Nagelkerke R Square
1	194.771[a]	.004	.102

a. Estimation terminated at iteration number 20 because maximum iterations has been reached. Final solution cannot be found.

Classification Table[a]

Observed			Predicted: Drug abuse_ny Nej	Predicted: Drug abuse_ny Ja	Predicted: Percentage Correct
Step 1	Drug abuse_ny	Nej	5091	0	100.0
		Ja	16	0	.0
	Overall Percentage				99.7

a. The cut value is ,500

Variables in the Equation

		B	S.E.	Wald	df	Sig.	Exp(B)	95% C.I.for EXP(B) Lower	Upper
Step 1[a]	Cluster modal			20.621	6	.002			
	Cluster modal(1)	.803	.817	.966	1	.326	2.233	.450	11.079
	Cluster modal(2)	-14.534	1755.838	.000	1	.993	.000	.000	.
	Cluster modal(3)	.713	1.156	.380	1	.537	2.040	.212	19.659
	Cluster modal(4)	2.140	.819	6.826	1	.009	8.496	1.707	42.299
	Cluster modal(5)	1.034	1.156	.799	1	.371	2.812	.292	27.124
	Cluster modal(6)	2.965	.734	16.319	1	<.001	19.392	4.601	81.728
	Constant	-6.669	.578	133.244	1	<.001	.001		

a. Variable(s) entered on step 1: Cluster modal.

Logistic Regression

Notes

Output Created		26-AUG-2025 11:46:50
Comments		
Input	Data	/Users/stevenlc/Library/CloudStorage/OneDrive-Privat/ICloud filer/Doktorander/Rickard/Artikel 4/Rickardonlywomen7classmodel.sav
	Active Dataset	DataSet4
	File Label	Scored Data File
	Filter	<none>
	Weight	<none>
	Split File	<none>
	N of Rows in Working Data File	5681
Missing Value Handling	Definition of Missing	User-defined missing values are treated as missing

Syntax		LOGISTIC REGRESSION VARIABLES HAD_probable_depression_ny<br>/METHOD=ENTER clu# fodelselandmammapappa_ny utbildningmammapappa_ny Birthyear_decades<br>/CONTRAST (clu#)=Indicator(1)<br>/CONTRAST (fodelselandmammapappa_ny)=Indicator(1)<br>/CONTRAST (utbildningmammapappa_ny)=Indicator(1)<br>/CONTRAST (Birthyear_decades)=Indicator(1)<br>/PRINT=CI(95)<br>/CRITERIA=PIN(0.05) POUT(0.10) ITERATE(20) CUT(0.5).
Resources	Processor Time	00:00:00,26
	Elapsed Time	00:00:00,00

Case Processing Summary

Unweighted Cases[a]		N	Percent
Selected Cases	Included in Analysis	4380	77.1
	Missing Cases	1301	22.9
	Total	5681	100.0
Unselected Cases		0	.0
Total		5681	100.0

a. If weight is in effect, see classification table for the total number of cases.

Dependent Variable Encoding

Original Value	Internal Value
Nej	0
Ja	1

Categorical Variables Codings

		Frequency	Parameter coding (1)	(2)	(3)	(4)	(5)	(6)
Cluster modal	1	2045	.000	.000	.000	.000	.000	.000
	2	927	1.000	.000	.000	.000	.000	.000
	3	444	.000	1.000	.000	.000	.000	.000
	4	326	.000	.000	1.000	.000	.000	.000
	5	238	.000	.000	.000	1.000	.000	.000
	6	240	.000	.000	.000	.000	1.000	.000
	7	160	.000	.000	.000	.000	.000	1.000
Birthyear_decades	18-27	699	.000	.000	.000	.000	.000	
	28-37	737	1.000	.000	.000	.000	.000	
	38-47	834	.000	1.000	.000	.000	.000	
	48-57	879	.000	.000	1.000	.000	.000	
	58-67	881	.000	.000	.000	1.000	.000	
	68-74	350	.000	.000	.000	.000	1.000	
utbildningmammapappa_ny	Minst en förälder högre utb än grundskola	2363	.000					
	Båda föräldrarna grundskola	2017	1.000					
fodelselandmammapappa_ny	Minst en förälder född i norden	4107	.000					
	Båda födda utanför Norden	273	1.000					

Block 0: Beginning Block

Classification Table[a,b]

Observed			Predicted Depression_ny Nej	Ja	Percentage Correct
Step 0	Depression_ny	Nej	4017	0	100.0
		Ja	363	0	.0
	Overall Percentage				91.7

a. Constant is included in the model.

b. The cut value is ,500

Variables in the Equation

		B	S.E.	Wald	df	Sig.	Exp(B)
Step 0	Constant	-2.404	.055	1923.812	1	.000	.090

Variables not in the Equation

			Score	df	Sig.
Step 0	Variables	Cluster modal	176.742	6	<.001
		Cluster modal(1)	.060	1	.806
		Cluster modal(2)	2.552	1	.110
		Cluster modal(3)	59.635	1	<.001
		Cluster modal(4)	2.302	1	.129
		Cluster modal(5)	45.826	1	<.001
		Cluster modal(6)	33.256	1	<.001
		fodelselandmammapappa_ny(1)	12.149	1	<.001
		utbildningmammapappa_ny(1)	.620	1	.431
		Birthyear_decades	17.208	5	.004
		Birthyear_decades(1)	1.346	1	.246
		Birthyear_decades(2)	.674	1	.412
		Birthyear_decades(3)	.000	1	.983
		Birthyear_decades(4)	5.412	1	.020
		Birthyear_decades(5)	6.912	1	.009
	Overall Statistics		197.386	13	<.001

Block 1: Method = Enter

Omnibus Tests of Model Coefficients

		Chi-square	df	Sig.
Step 1	Step	170.826	13	<.001
	Block	170.826	13	<.001
	Model	170.826	13	<.001

Model Summary

Step	-2 Log likelihood	Cox & Snell R Square	Nagelkerke R Square
1	2332.254[a]	.038	.088

a. Estimation terminated at iteration number 6 because parameter estimates changed by less than ,001.

Classification Table[a]

	Observed	Predicted	
		Depression_ny	Percentage

			Nej	Ja	Correct
Step 1	Depression_ny	Nej	4017	0	100.0
		Ja	363	0	.0
	Overall Percentage				91.7

a. The cut value is ,500

Variables in the Equation

		B	S.E.	Wald	df	Sig.	Exp(B)	95% C.I.for EXP(B) Lower	Upper
Step 1[a]	Cluster modal			145.047	6	<.001			
	Cluster modal(1)	.624	.164	14.421	1	<.001	1.867	1.353	2.577
	Cluster modal(2)	.381	.224	2.901	1	.089	1.464	.944	2.271
	Cluster modal(3)	1.638	.179	84.160	1	<.001	5.147	3.627	7.304
	Cluster modal(4)	1.005	.236	18.077	1	<.001	2.732	1.719	4.342
	Cluster modal(5)	1.686	.198	72.862	1	<.001	5.399	3.666	7.951
	Cluster modal(6)	1.721	.226	57.924	1	<.001	5.588	3.588	8.704
	fodelselandmammapappa_ny(1)	.516	.192	7.228	1	.007	1.676	1.150	2.442
	utbildningmammapappa_ny(1)	.271	.129	4.442	1	.035	1.311	1.019	1.687
	Birthyear_decades			12.172	5	.033			
	Birthyear_decades(1)	-.276	.182	2.290	1	.130	.759	.531	1.085
	Birthyear_decades(2)	-.346	.182	3.595	1	.058	.708	.495	1.012
	Birthyear_decades(3)	-.478	.190	6.340	1	.012	.620	.427	.899
	Birthyear_decades(4)	-.610	.207	8.728	1	.003	.543	.362	.814
	Birthyear_decades(5)	-.798	.302	7.003	1	.008	.450	.249	.813
	Constant	-2.864	.161	315.081	1	<.001	.057		

a. Variable(s) entered on step 1: Cluster modal, fodelselandmammapappa_ny, utbildningmammapappa_ny, Birthyear_decades.

Logistic Regression

Notes

Output Created	26-AUG-2025 11:46:50
Comments	

Input	Data	/Users/stevenlc/Library/CloudStorage/OneDrive-Privat/ICloud filer/Doktorander/Rickard/Artikel 4/Rickardonlywomen7classmodel.sav
	Active Dataset	DataSet4
	File Label	Scored Data File
	Filter	<none>
	Weight	<none>
	Split File	<none>
	N of Rows in Working Data File	5681
Missing Value Handling	Definition of Missing	User-defined missing values are treated as missing
Syntax		LOGISTIC REGRESSION VARIABLES HAD_probable_ångest_ny /METHOD=ENTER clu# fodelselandmammapappa_ny utbildningmammapappa_ny Birthyear_decades /CONTRAST (clu#)=Indicator(1) /CONTRAST (fodelselandmammapappa_ny)=Indicator(1) /CONTRAST (utbildningmammapappa_ny)=Indicator(1) /CONTRAST (Birthyear_decades)=Indicator(1) /PRINT=CI(95) /CRITERIA=PIN(0.05) POUT(0.10) ITERATE(20) CUT(0.5).
Resources	Processor Time	00:00:00,26
	Elapsed Time	00:00:00,00

Case Processing Summary

Unweighted Cases[a]		N	Percent
Selected Cases	Included in Analysis	4385	77.2

		N	Percent
	Missing Cases	1296	22.8
	Total	5681	100.0
Unselected Cases		0	.0
Total		5681	100.0

a. If weight is in effect, see classification table for the total number of cases.

Dependent Variable Encoding

Original Value	Internal Value
Nej	0
Ja	1

Categorical Variables Codings

		Frequency	Parameter coding (1)	(2)	(3)	(4)	(5)	(6)
Cluster modal	1	2053	.000	.000	.000	.000	.000	.000
	2	925	1.000	.000	.000	.000	.000	.000
	3	450	.000	1.000	.000	.000	.000	.000
	4	329	.000	.000	1.000	.000	.000	.000
	5	233	.000	.000	.000	1.000	.000	.000
	6	231	.000	.000	.000	.000	1.000	.000
	7	164	.000	.000	.000	.000	.000	1.000
Birthyear_decades	18-27	689	.000	.000	.000	.000	.000	
	28-37	732	1.000	.000	.000	.000	.000	
	38-47	834	.000	1.000	.000	.000	.000	
	48-57	877	.000	.000	1.000	.000	.000	
	58-67	892	.000	.000	.000	1.000	.000	
	68-74	361	.000	.000	.000	.000	1.000	
utbildningmammapappa_ny	Minst en förälder högre utb än grundskola	2366	.000					
	Båda föräldrarna grundskola	2019	1.000					
fodelselandmammapappa_ny	Minst en förälder född i norden	4106	.000					
	Båda födda utanför Norden	279	1.000					

Block 0: Beginning Block

Classification Table[a,b]

	Observed		Predicted: Anxiety_ny Nej	Predicted: Anxiety_ny Ja	Predicted: Percentage Correct
Step 0	Anxiety_ny	Nej	4111	0	100.0
		Ja	274	0	.0
	Overall Percentage				93.8

a. Constant is included in the model.

b. The cut value is ,500

Variables in the Equation

		B	S.E.	Wald	df	Sig.	Exp(B)
Step 0	Constant	-2.708	.062	1884.169	1	.000	.067

Variables not in the Equation

			Score	df	Sig.
Step 0	Variables	Cluster modal	201.726	6	<.001
		Cluster modal(1)	.076	1	.783
		Cluster modal(2)	1.583	1	.208
		Cluster modal(3)	30.826	1	<.001
		Cluster modal(4)	.015	1	.902
		Cluster modal(5)	59.275	1	<.001
		Cluster modal(6)	77.388	1	<.001
		fodelselandmammapappa_ny(1)	7.295	1	.007
		utbildningmammapappa_ny(1)	10.722	1	.001
		Birthyear_decades	66.571	5	<.001
		Birthyear_decades(1)	1.910	1	.167
		Birthyear_decades(2)	1.279	1	.258
		Birthyear_decades(3)	.035	1	.852
		Birthyear_decades(4)	21.245	1	<.001
		Birthyear_decades(5)	8.125	1	.004
	Overall Statistics		261.068	13	<.001

Block 1: Method = Enter

Omnibus Tests of Model Coefficients

		Chi-square	df	Sig.
Step 1	Step	216.446	13	<.001

	Block	216.446	13	<.001
	Model	216.446	13	<.001

Model Summary

Step	-2 Log likelihood	Cox & Snell R Square	Nagelkerke R Square
1	1833.568[a]	.048	.129

a. Estimation terminated at iteration number 6 because parameter estimates changed by less than ,001.

Classification Table[a]

Observed			Predicted Anxiety_ny Nej	Ja	Percentage Correct
Step 1	Anxiety_ny	Nej	4111	0	100.0
		Ja	272	2	.7
	Overall Percentage				93.8

a. The cut value is ,500

Variables in the Equation

		B	S.E.	Wald	df	Sig.	Exp(B)	95% C.I.for EXP(B) Lower	Upper
Step 1[a]	Cluster modal			152.829	6	<.001			
	Cluster modal(1)	.653	.195	11.238	1	<.001	1.921	1.312	2.814
	Cluster modal(2)	.550	.258	4.555	1	.033	1.733	1.046	2.873
	Cluster modal(3)	1.596	.213	55.990	1	<.001	4.935	3.248	7.496
	Cluster modal(4)	.929	.302	9.462	1	.002	2.531	1.401	4.575
	Cluster modal(5)	1.988	.221	80.544	1	<.001	7.297	4.728	11.263
	Cluster modal(6)	2.280	.235	94.341	1	<.001	9.778	6.172	15.491
	fodelselandmammapappa_ny(1)	.376	.221	2.895	1	.089	1.456	.944	2.245
	utbildningmammapappa_ny(1)	.159	.152	1.098	1	.295	1.172	.871	1.579
	Birthyear_decades			49.839	5	<.001			
	Birthyear_decades(1)	-.672	.191	12.330	1	<.001	.511	.351	.743
	Birthyear_decades(2)	-1.055	.205	26.422	1	<.001	.348	.233	.521
	Birthyear_decades(3)	-.912	.203	20.275	1	<.001	.402	.270	.598
	Birthyear_decades(4)	-1.527	.254	36.163	1	<.001	.217	.132	.357
	Birthyear_decades(5)	-1.391	.362	14.794	1	<.001	.249	.123	.506
	Constant	-2.779	.171	263.080	1	<.001	.062		

a. Variable(s) entered on step 1: Cluster modal, fodelselandmammapappa_ny, utbildningmammapappa_ny, Birthyear_decades.

Logistic Regression

Notes

Output Created		26-AUG-2025 11:46:50
Comments		
Input	Data	/Users/stevenlc/Library/CloudStorage/OneDrive-Privat/ICloud filer/Doktorander/Rickard/Artikel 4/Rickardonlywomen7classmodel.sav
	Active Dataset	DataSet4
	File Label	Scored Data File
	Filter	<none>
	Weight	<none>
	Split File	<none>
	N of Rows in Working Data File	5681
Missing Value Handling	Definition of Missing	User-defined missing values are treated as missing

Syntax		LOGISTIC REGRESSION VARIABLES PTSD_score_pos_ny<br>/METHOD=ENTER clu# fodelselandmammapappa_ny utbildningmammapappa_ny Birthyear_decades<br>/CONTRAST (clu#)=Indicator(1)<br>/CONTRAST (fodelselandmammapappa_ny)=Indicator(1)<br>/CONTRAST (utbildningmammapappa_ny)=Indicator(1)<br>/CONTRAST (Birthyear_decades)=Indicator(1)<br>/PRINT=CI(95)<br>/CRITERIA=PIN(0.05) POUT(0.10) ITERATE(20) CUT(0.5).
Resources	Processor Time	00:00:00,26
	Elapsed Time	00:00:01,00

Case Processing Summary

Unweighted Cases[a]		N	Percent
Selected Cases	Included in Analysis	4325	76.1
	Missing Cases	1356	23.9
	Total	5681	100.0
Unselected Cases		0	.0
Total		5681	100.0

a. If weight is in effect, see classification table for the total number of cases.

Dependent Variable Encoding

Original Value	Internal Value
Nej	0
Ja	1

Categorical Variables Codings

		Frequency	Parameter coding (1)	(2)	(3)	(4)	(5)	(6)
Cluster modal	1	2043	.000	.000	.000	.000	.000	.000
	2	905	1.000	.000	.000	.000	.000	.000
	3	436	.000	1.000	.000	.000	.000	.000
	4	322	.000	.000	1.000	.000	.000	.000
	5	231	.000	.000	.000	1.000	.000	.000
	6	225	.000	.000	.000	.000	1.000	.000
	7	163	.000	.000	.000	.000	.000	1.000
Birthyear_decades	18-27	696	.000	.000	.000	.000	.000	
	28-37	735	1.000	.000	.000	.000	.000	
	38-47	819	.000	1.000	.000	.000	.000	
	48-57	859	.000	.000	1.000	.000	.000	
	58-67	864	.000	.000	.000	1.000	.000	
	68-74	352	.000	.000	.000	.000	1.000	
utbildningmammapappa_ny	Minst en förälder högre utb än grundskola	2338	.000					
	Båda föräldrarna grundskola	1987	1.000					
fodelselandmammapappa_ny	Minst en förälder född i norden	4053	.000					
	Båda födda utanför Norden	272	1.000					

Block 0: Beginning Block

Classification Table[a,b]

Observed			Predicted PTSS_ny Nej	Ja	Percentage Correct
Step 0	PTSS_ny	Nej	3953	0	100.0
		Ja	372	0	.0
	Overall Percentage				91.4

a. Constant is included in the model.

b. The cut value is ,500

Variables in the Equation

		B	S.E.	Wald	df	Sig.	Exp(B)
Step 0	Constant	-2.363	.054	1899.042	1	.000	.094

Variables not in the Equation

			Score	df	Sig.
Step 0	Variables	Cluster modal	397.158	6	<.001
		Cluster modal(1)	.832	1	.362
		Cluster modal(2)	1.370	1	.242
		Cluster modal(3)	23.180	1	<.001
		Cluster modal(4)	2.187	1	.139
		Cluster modal(5)	71.592	1	<.001
		Cluster modal(6)	227.627	1	<.001
		fodelselandmammapappa_ny(1)	10.645	1	.001
		utbildningmammapappa_ny (1)	13.614	1	<.001
		Birthyear_decades	52.235	5	<.001
		Birthyear_decades(1)	1.995	1	.158
		Birthyear_decades(2)	4.570	1	.033
		Birthyear_decades(3)	.179	1	.672
		Birthyear_decades(4)	11.789	1	<.001
		Birthyear_decades(5)	6.934	1	.008
	Overall Statistics		446.963	13	<.001

Block 1: Method = Enter

Omnibus Tests of Model Coefficients

		Chi-square	df	Sig.
Step 1	Step	356.096	13	<.001
	Block	356.096	13	<.001
	Model	356.096	13	<.001

Model Summary

Step	-2 Log likelihood	Cox & Snell R Square	Nagelkerke R Square
1	2180.184[a]	.079	.178

a. Estimation terminated at iteration number 6 because parameter estimates changed by less than ,001.

Classification Table[a]

Observed		Predicted		
		PTSS_ny		Percentage Correct
		Nej	Ja	

Step 1	PTSS_ny	Nej	3941	12	99.7
		Ja	355	17	4.6
	Overall Percentage				91.5

a. The cut value is ,500

Variables in the Equation

		B	S.E.	Wald	df	Sig.	Exp(B)	95% C.I.for EXP(B) Lower	Upper
Step 1[a]	Cluster modal			281.532	6	<.001			
	Cluster modal(1)	.958	.184	27.087	1	<.001	2.606	1.817	3.738
	Cluster modal(2)	1.321	.208	40.246	1	<.001	3.746	2.491	5.633
	Cluster modal(3)	1.796	.205	76.710	1	<.001	6.024	4.030	9.003
	Cluster modal(4)	1.537	.250	37.778	1	<.001	4.651	2.849	7.593
	Cluster modal(5)	2.372	.209	128.420	1	<.001	10.722	7.114	16.161
	Cluster modal(6)	3.198	.212	227.811	1	<.001	24.485	16.164	37.091
	fodelselandmammapappa_ny(1)	.486	.198	6.009	1	.014	1.626	1.102	2.398
	utbildningmammapappa_ny(1)	.063	.135	.216	1	.642	1.065	.817	1.389
	Birthyear_decades			40.927	5	<.001			
	Birthyear_decades(1)	-.579	.175	10.946	1	<.001	.560	.398	.790
	Birthyear_decades(2)	-1.094	.192	32.358	1	<.001	.335	.230	.488
	Birthyear_decades(3)	-.792	.185	18.310	1	<.001	.453	.315	.651
	Birthyear_decades(4)	-1.014	.210	23.327	1	<.001	.363	.240	.548
	Birthyear_decades(5)	-.929	.296	9.830	1	.002	.395	.221	.706
	Constant	-2.880	.168	295.551	1	<.001	.056		

a. Variable(s) entered on step 1: Cluster modal, fodelselandmammapappa_ny, utbildningmammapappa_ny, Birthyear_decades.

Logistic Regression

Notes

Output Created		26-AUG-2025 11:46:51
Comments		
Input	Data	/Users/stevenlc/Library/CloudStorage/OneDrive-Privat/ICloud filer/Doktorander/Rickard/Artikel 4/Rickardonlywomen7classmodel.sav

	Active Dataset	DataSet4
	File Label	Scored Data File
	Filter	<none>
	Weight	<none>
	Split File	<none>
	N of Rows in Working Data File	5681
Missing Value Handling	Definition of Missing	User-defined missing values are treated as missing
Syntax		LOGISTIC REGRESSION VARIABLES Any_selfharm_ny /METHOD=ENTER clu# fodelselandmammapappa_ny utbildningmammapappa_ny Birthyear_decades /CONTRAST (clu#)=Indicator(1) /CONTRAST (fodelselandmammapappa_ny)=Indicator(1) /CONTRAST (utbildningmammapappa_ny)=Indicator(1) /CONTRAST (Birthyear_decades)=Indicator(1) /PRINT=CI(95) /CRITERIA=PIN(0.05) POUT(0.10) ITERATE(20) CUT(0.5).
Resources	Processor Time	00:00:00,26
	Elapsed Time	00:00:00,00

Case Processing Summary

Unweighted Cases[a]		N	Percent
Selected Cases	Included in Analysis	4536	79.8
	Missing Cases	1145	20.2
	Total	5681	100.0
Unselected Cases		0	.0
Total		5681	100.0

a. If weight is in effect, see classification table for the total number of cases.

Dependent Variable Encoding

Original Value	Internal Value
Nej	0
Ja	1

Categorical Variables Codings

		Frequency	Parameter coding (1)	(2)	(3)	(4)	(5)	(6)
Cluster modal	1	2125	.000	.000	.000	.000	.000	.000
	2	954	1.000	.000	.000	.000	.000	.000
	3	460	.000	1.000	.000	.000	.000	.000
	4	338	.000	.000	1.000	.000	.000	.000
	5	246	.000	.000	.000	1.000	.000	.000
	6	243	.000	.000	.000	.000	1.000	.000
	7	170	.000	.000	.000	.000	.000	1.000
Birthyear_decades	18-27	714	.000	.000	.000	.000	.000	
	28-37	759	1.000	.000	.000	.000	.000	
	38-47	861	.000	1.000	.000	.000	.000	
	48-57	903	.000	.000	1.000	.000	.000	
	58-67	924	.000	.000	.000	1.000	.000	
	68-74	375	.000	.000	.000	.000	1.000	
utbildningmammapappa_ny	Minst en förälder högre utb än grundskola	2438	.000					
	Båda föräldrarna grundskola	2098	1.000					
fodelselandmammapappa_ny	Minst en förälder född i norden	4246	.000					
	Båda födda utanför Norden	290	1.000					

Block 0: Beginning Block

Classification Table[a,b]

Observed			Predicted Self-harm_ny Nej	Ja	Percentage Correct
Step 0	Self-harm_ny	Nej	3902	0	100.0
		Ja	634	0	.0
	Overall Percentage				86.0

a. Constant is included in the model.

b. The cut value is ,500

Variables in the Equation

		B	S.E.	Wald	df	Sig.	Exp(B)
Step 0	Constant	-1.817	.043	1800.971	1	.000	.162

Variables not in the Equation

			Score	df	Sig.
Step 0	Variables	Cluster modal	567.153	6	<.001
		Cluster modal(1)	3.443	1	.064
		Cluster modal(2)	3.556	1	.059
		Cluster modal(3)	76.835	1	<.001
		Cluster modal(4)	2.654	1	.103
		Cluster modal(5)	70.127	1	<.001
		Cluster modal(6)	295.436	1	<.001
		fodelselandmammapappa_ny(1)	.072	1	.788
		utbildningmammapappa_ny(1)	83.248	1	<.001
		Birthyear_decades	201.612	5	<.001
		Birthyear_decades(1)	24.235	1	<.001
		Birthyear_decades(2)	.340	1	.560
		Birthyear_decades(3)	11.933	1	<.001
		Birthyear_decades(4)	47.976	1	<.001
		Birthyear_decades(5)	30.322	1	<.001
	Overall Statistics		738.567	13	<.001

Block 1: Method = Enter

Omnibus Tests of Model Coefficients

		Chi-square	df	Sig.
Step 1	Step	662.508	13	<.001
	Block	662.508	13	<.001
	Model	662.508	13	<.001

Model Summary

Step	-2 Log likelihood	Cox & Snell R Square	Nagelkerke R Square
1	3007.543[a]	.136	.245

a. Estimation terminated at iteration number 6 because parameter estimates changed by less than ,001.

Classification Table[a]

Observed			Predicted Self-harm_ny Nej	Ja	Percentage Correct
Step 1	Self-harm_ny	Nej	3827	75	98.1
		Ja	525	109	17.2
	Overall Percentage				86.8

a. The cut value is ,500

Variables in the Equation

		B	S.E.	Wald	df	Sig.	Exp(B)	95% C.I.for EXP(B) Lower	Upper
Step 1[a]	Cluster modal			409.855	6	<.001			
	Cluster modal(1)	1.023	.136	56.836	1	<.001	2.781	2.132	3.628
	Cluster modal(2)	.856	.182	22.106	1	<.001	2.353	1.647	3.362
	Cluster modal(3)	2.045	.160	163.831	1	<.001	7.733	5.654	10.577
	Cluster modal(4)	1.515	.201	56.933	1	<.001	4.548	3.069	6.741
	Cluster modal(5)	2.130	.176	147.045	1	<.001	8.412	5.962	11.868
	Cluster modal(6)	3.408	.193	311.112	1	<.001	30.205	20.683	44.111
	fodelselandmammapappa_ny(1)	-.401	.194	4.268	1	.039	.670	.458	.980
	utbildningmammapappa_ny(1)	-.204	.113	3.219	1	.073	.816	.653	1.019
	Birthyear_decades			123.961	5	<.001			
	Birthyear_decades(1)	-.537	.136	15.666	1	<.001	.584	.448	.763
	Birthyear_decades(2)	-1.071	.147	53.130	1	<.001	.343	.257	.457
	Birthyear_decades(3)	-1.409	.161	76.711	1	<.001	.244	.178	.335
	Birthyear_decades(4)	-1.574	.180	76.644	1	<.001	.207	.146	.295
	Birthyear_decades(5)	-1.743	.284	37.576	1	<.001	.175	.100	.306
	Constant	-1.870	.124	225.930	1	<.001	.154		

a. Variable(s) entered on step 1: Cluster modal, fodelselandmammapappa_ny, utbildningmammapappa_ny, Birthyear_decades.

Logistic Regression

Notes

Output Created		26-AUG-2025 11:46:51
Comments		
Input	Data	/Users/stevenlc/Library/CloudStorage/OneDrive-Privat/ICloud filer/Doktorander/Rickard/Artikel 4/Rickardonlywomen7classmodel.sav
	Active Dataset	DataSet4
	File Label	Scored Data File
	Filter	<none>
	Weight	<none>
	Split File	<none>
	N of Rows in Working Data File	5681
Missing Value Handling	Definition of Missing	User-defined missing values are treated as missing
Syntax		LOGISTIC REGRESSION VARIABLES Symtom_score_måttlig_ny<br>/METHOD=ENTER clu# fodelselandmammapappa_ny utbildningmammapappa_ny Birthyear_decades<br>/CONTRAST (clu#)=Indicator(1)<br>/CONTRAST (fodelselandmammapappa_ny)=Indicator(1)<br>/CONTRAST (utbildningmammapappa_ny)=Indicator(1)<br>/CONTRAST (Birthyear_decades)=Indicator(1)<br>/PRINT=CI(95)<br>/CRITERIA=PIN(0.05) POUT(0.10) ITERATE(20) CUT(0.5).
Resources	Processor Time	00:00:00,26
	Elapsed Time	00:00:00,00

Case Processing Summary

Unweighted Cases[a]		N	Percent
Selected Cases	Included in Analysis	4192	73.8
	Missing Cases	1489	26.2
	Total	5681	100.0
Unselected Cases		0	.0
Total		5681	100.0

a. If weight is in effect, see classification table for the total number of cases.

Dependent Variable Encoding

Original Value	Internal Value
Nej	0
Ja	1

Categorical Variables Codings

			Parameter coding					
		Frequency	(1)	(2)	(3)	(4)	(5)	(6)
Cluster modal	1	1960	.000	.000	.000	.000	.000	.000
	2	888	1.000	.000	.000	.000	.000	.000
	3	422	.000	1.000	.000	.000	.000	.000
	4	317	.000	.000	1.000	.000	.000	.000
	5	230	.000	.000	.000	1.000	.000	.000
	6	216	.000	.000	.000	.000	1.000	.000
	7	159	.000	.000	.000	.000	.000	1.000
Birthyear_decades	18-27	677	.000	.000	.000	.000	.000	
	28-37	717	1.000	.000	.000	.000	.000	
	38-47	811	.000	1.000	.000	.000	.000	
	48-57	836	.000	.000	1.000	.000	.000	
	58-67	825	.000	.000	.000	1.000	.000	
	68-74	326	.000	.000	.000	.000	1.000	
utbildningmammapappa_ny	Minst en förälder högre utb än grundskola	2284	.000					
	Båda föräldrarna grundskola	1908	1.000					
fodelselandmammapappa_ny	Minst en förälder född i norden	3930	.000					
	Båda födda utanför Norden	262	1.000					

Block 0: Beginning Block

Classification Table[a,b]

Observed			Predicted Somatization_ny Nej	Predicted Somatization_ny Ja	Percentage Correct
Step 0	Somatization_ny	Nej	3877	0	100.0
		Ja	315	0	.0
	Overall Percentage				92.5

a. Constant is included in the model.

b. The cut value is ,500

Variables in the Equation

		B	S.E.	Wald	df	Sig.	Exp(B)
Step 0	Constant	-2.510	.059	1835.765	1	.000	.081

Variables not in the Equation

			Score	df	Sig.
Step 0	Variables	Cluster modal	143.826	6	<.001
		Cluster modal(1)	.459	1	.498
		Cluster modal(2)	.410	1	.522
		Cluster modal(3)	11.315	1	<.001
		Cluster modal(4)	3.942	1	.047
		Cluster modal(5)	24.742	1	<.001
		Cluster modal(6)	74.023	1	<.001
		fodelselandmammapappa_ny(1)	15.589	1	<.001
		utbildningmammapappa_ny(1)	5.889	1	.015
		Birthyear_decades	11.531	5	.042
		Birthyear_decades(1)	4.015	1	.045
		Birthyear_decades(2)	.537	1	.464
		Birthyear_decades(3)	7.909	1	.005
		Birthyear_decades(4)	.196	1	.658
		Birthyear_decades(5)	.300	1	.584
	Overall Statistics		178.414	13	<.001

Block 1: Method = Enter

Omnibus Tests of Model Coefficients

		Chi-square	df	Sig.
Step 1	Step	151.890	13	<.001
	Block	151.890	13	<.001
	Model	151.890	13	<.001

Model Summary

Step	-2 Log likelihood	Cox & Snell R Square	Nagelkerke R Square
1	2084.491[a]	.036	.086

a. Estimation terminated at iteration number 6 because parameter estimates changed by less than ,001.

Classification Table[a]

	Observed		Predicted Somatization_ny Nej	Ja	Percentage Correct
Step 1	Somatization_ny	Nej	3876	1	100.0
		Ja	315	0	.0
	Overall Percentage				92.5

a. The cut value is ,500

Variables in the Equation

		B	S.E.	Wald	df	Sig.	Exp(B)	95% C.I.for EXP(B) Lower	Upper
Step 1[a]	Cluster modal			125.416	6	<.001			
	Cluster modal(1)	.714	.178	16.019	1	<.001	2.043	1.440	2.899
	Cluster modal(2)	.754	.212	12.689	1	<.001	2.126	1.404	3.220
	Cluster modal(3)	1.276	.210	37.004	1	<.001	3.582	2.374	5.403
	Cluster modal(4)	1.080	.244	19.544	1	<.001	2.944	1.824	4.752
	Cluster modal(5)	1.661	.223	55.403	1	<.001	5.267	3.401	8.158
	Cluster modal(6)	2.156	.221	94.835	1	<.001	8.640	5.598	13.336
	fodelselandmammapappa_ny(1)	.784	.199	15.451	1	<.001	2.190	1.482	3.238
	utbildningmammapappa_ny(1)	.341	.136	6.279	1	.012	1.407	1.077	1.837
	Birthyear_decades			9.120	5	.104			
	Birthyear_decades(1)	-.251	.230	1.191	1	.275	.778	.496	1.221
	Birthyear_decades(2)	-.083	.218	.143	1	.705	.921	.601	1.412
	Birthyear_decades(3)	.244	.211	1.335	1	.248	1.276	.844	1.931
	Birthyear_decades(4)	.166	.225	.546	1	.460	1.181	.760	1.836

	Birthyear_decades(5)	.414	.276	2.244	1	.134	1.512	.880	2.599
	Constant	-3.521	.196	321.635	1	<.001	.030		

a. Variable(s) entered on step 1: Cluster modal, fodelselandmammapappa_ny, utbildningmammapappa_ny, Birthyear_decades.

Logistic Regression

Notes

Output Created		26-AUG-2025 11:46:51
Comments		
Input	Data	/Users/stevenlc/Library/CloudStorage/OneDrive-Privat/ICloud filer/Doktorander/Rickard/Artikel 4/Rickardonlywomen7classmodel.sav
	Active Dataset	DataSet4
	File Label	Scored Data File
	Filter	<none>
	Weight	<none>
	Split File	<none>
	N of Rows in Working Data File	5681
Missing Value Handling	Definition of Missing	User-defined missing values are treated as missing

Syntax		LOGISTIC REGRESSION VARIABLES IBS_ny<br>/METHOD=ENTER clu# fodelselandmammapappa_ny utbildningmammapappa_ny Birthyear_decades<br>/CONTRAST (clu#)=Indicator(1)<br>/CONTRAST (fodelselandmammapappa_ny)=Indicator(1)<br>/CONTRAST (utbildningmammapappa_ny)=Indicator(1)<br>/CONTRAST (Birthyear_decades)=Indicator(1)<br>/PRINT=CI(95)<br>/CRITERIA=PIN(0.05) POUT(0.10) ITERATE(20) CUT(0.5).
Resources	Processor Time	00:00:00,26
	Elapsed Time	00:00:00,00

Case Processing Summary

Unweighted Cases[a]		N	Percent
Selected Cases	Included in Analysis	4555	80.2
	Missing Cases	1126	19.8
	Total	5681	100.0
Unselected Cases		0	.0
Total		5681	100.0

a. If weight is in effect, see classification table for the total number of cases.

Dependent Variable Encoding

Original Value	Internal Value
Nej	0
Ja	1

Categorical Variables Codings

	Frequency	Parameter coding

			(1)	(2)	(3)	(4)	(5)	(6)
Cluster modal	1	2138	.000	.000	.000	.000	.000	.000
	2	957	1.000	.000	.000	.000	.000	.000
	3	461	.000	1.000	.000	.000	.000	.000
	4	339	.000	.000	1.000	.000	.000	.000
	5	246	.000	.000	.000	1.000	.000	.000
	6	244	.000	.000	.000	.000	1.000	.000
	7	170	.000	.000	.000	.000	.000	1.000
Birthyear_decades	18-27	717	.000	.000	.000	.000	.000	
	28-37	760	1.000	.000	.000	.000	.000	
	38-47	862	.000	1.000	.000	.000	.000	
	48-57	908	.000	.000	1.000	.000	.000	
	58-67	929	.000	.000	.000	1.000	.000	
	68-74	379	.000	.000	.000	.000	1.000	
utbildningmammapappa_ny	Minst en förälder högre utb än grundskola	2448	.000					
	Båda föräldrarna grundskola	2107	1.000					
fodelselandmammapappa_ny	Minst en förälder född i norden	4261	.000					
	Båda födda utanför Norden	294	1.000					

Block 0: Beginning Block

Classification Table[a,b]

Observed			Predicted IBS_ny Nej	Predicted IBS_ny Ja	Percentage Correct
Step 0	IBS_ny	Nej	4270	0	100.0
		Ja	285	0	.0
	Overall Percentage				93.7

a. Constant is included in the model.
b. The cut value is ,500

Variables in the Equation

		B	S.E.	Wald	df	Sig.	Exp(B)
Step 0	Constant	-2.707	.061	1957.593	1	.000	.067

Variables not in the Equation

	Score	df	Sig.

Step 0	Variables	Cluster modal	38.603	6	<.001
		Cluster modal(1)	1.067	1	.302
		Cluster modal(2)	.711	1	.399
		Cluster modal(3)	3.297	1	.069
		Cluster modal(4)	3.199	1	.074
		Cluster modal(5)	6.994	1	.008
		Cluster modal(6)	15.924	1	<.001
		fodelselandmammapappa_ny(1)	1.198	1	.274
		utbildningmammapappa_ny(1)	2.611	1	.106
		Birthyear_decades	15.542	5	.008
		Birthyear_decades(1)	.830	1	.362
		Birthyear_decades(2)	.021	1	.884
		Birthyear_decades(3)	1.980	1	.159
		Birthyear_decades(4)	3.821	1	.051
		Birthyear_decades(5)	.902	1	.342
	Overall Statistics		56.935	13	<.001

Block 1: Method = Enter

Omnibus Tests of Model Coefficients

		Chi-square	df	Sig.
Step 1	Step	54.375	13	<.001
	Block	54.375	13	<.001
	Model	54.375	13	<.001

Model Summary

Step	-2 Log likelihood	Cox & Snell R Square	Nagelkerke R Square
1	2077.159[a]	.012	.032

a. Estimation terminated at iteration number 6 because parameter estimates changed by less than ,001.

Classification Table[a]

Observed			Predicted: IBS_ny Nej	Predicted: IBS_ny Ja	Percentage Correct
Step 1	IBS_ny	Nej	4270	0	100.0

	Ja	285	0	.0
	Overall Percentage			93.7

a. The cut value is ,500

Variables in the Equation

		B	S.E.	Wald	df	Sig.	Exp(B)	95% C.I.for EXP(B)	
								Lower	Upper
Step 1[a]	Cluster modal			38.681	6	<.001			
	Cluster modal(1)	.291	.178	2.673	1	.102	1.338	.944	1.897
	Cluster modal(2)	.469	.209	5.062	1	.024	1.599	1.062	2.406
	Cluster modal(3)	.728	.222	10.720	1	.001	2.070	1.339	3.200
	Cluster modal(4)	.694	.248	7.823	1	.005	2.002	1.231	3.257
	Cluster modal(5)	.934	.239	15.288	1	<.001	2.544	1.593	4.064
	Cluster modal(6)	1.226	.250	24.013	1	<.001	3.406	2.086	5.561
	fodelselandmammapappa_ny(1)	-.296	.285	1.077	1	.299	.744	.426	1.301
	utbildningmammapappa_ny(1)	.017	.139	.015	1	.904	1.017	.774	1.337
	Birthyear_decades			13.418	5	.020			
	Birthyear_decades(1)	.459	.260	3.121	1	.077	1.583	.951	2.635
	Birthyear_decades(2)	.548	.253	4.691	1	.030	1.730	1.053	2.841
	Birthyear_decades(3)	.703	.252	7.766	1	.005	2.020	1.232	3.313
	Birthyear_decades(4)	.857	.256	11.216	1	<.001	2.355	1.427	3.889
	Birthyear_decades(5)	.915	.301	9.204	1	.002	2.496	1.382	4.506
	Constant	-3.651	.226	261.431	1	<.001	.026		

a. Variable(s) entered on step 1: Cluster modal, fodelselandmammapappa_ny, utbildningmammapappa_ny, Birthyear_decades.

Logistic Regression

Notes

Output Created		26-AUG-2025 11:46:51
Comments		
Input	Data	/Users/stevenlc/Library/CloudStorage/OneDrive-Privat/ICloud filer/Doktorander/Rickard/Artikel 4/Rickardonlywomen7classmodel.sav
	Active Dataset	DataSet4

	File Label	Scored Data File
	Filter	<none>
	Weight	<none>
	Split File	<none>
	N of Rows in Working Data File	5681
Missing Value Handling	Definition of Missing	User-defined missing values are treated as missing
Syntax		LOGISTIC REGRESSION VARIABLES Fibromyalgi_ny<br>/METHOD=ENTER clu# fodelselandmammapappa_ny utbildningmammapappa_ny Birthyear_decades<br>/CONTRAST (clu#)=Indicator(1)<br>/CONTRAST (fodelselandmammapappa_ny)=Indicator(1)<br>/CONTRAST (utbildningmammapappa_ny)=Indicator(1)<br>/CONTRAST (Birthyear_decades)=Indicator(1)<br>/PRINT=CI(95)<br>/CRITERIA=PIN(0.05) POUT(0.10) ITERATE(20) CUT(0.5).
Resources	Processor Time	00:00:00,32
	Elapsed Time	00:00:01,00

Case Processing Summary

Unweighted Cases[a]		N	Percent
Selected Cases	Included in Analysis	4555	80.2
	Missing Cases	1126	19.8
	Total	5681	100.0
Unselected Cases		0	.0
Total		5681	100.0

a. If weight is in effect, see classification table for the total number of cases.

Dependent Variable Encoding

Original Value	Internal Value
Nej	0
Ja	1

Categorical Variables Codings

		Frequency	Parameter coding (1)	(2)	(3)	(4)	(5)	(6)
Cluster modal	1	2138	.000	.000	.000	.000	.000	.000
	2	957	1.000	.000	.000	.000	.000	.000
	3	461	.000	1.000	.000	.000	.000	.000
	4	339	.000	.000	1.000	.000	.000	.000
	5	246	.000	.000	.000	1.000	.000	.000
	6	244	.000	.000	.000	.000	1.000	.000
	7	170	.000	.000	.000	.000	.000	1.000
Birthyear_decades	18-27	717	.000	.000	.000	.000	.000	
	28-37	760	1.000	.000	.000	.000	.000	
	38-47	862	.000	1.000	.000	.000	.000	
	48-57	908	.000	.000	1.000	.000	.000	
	58-67	929	.000	.000	.000	1.000	.000	
	68-74	379	.000	.000	.000	.000	1.000	
utbildningmammapappa_ny	Minst en förälder högre utb än grundskola	2448	.000					
	Båda föräldrarna grundskola	2107	1.000					
fodelselandmammapappa_ny	Minst en förälder född i norden	4261	.000					
	Båda födda utanför Norden	294	1.000					

Block 0: Beginning Block

Classification Table[a,b]

Observed			Predicted: Fibromyalgia_ny Nej	Ja	Percentage Correct
Step 0	Fibromyalgia_ny	Nej	4409	0	100.0
		Ja	146	0	.0
	Overall Percentage				96.8

a. Constant is included in the model.

b. The cut value is ,500

Variables in the Equation

		B	S.E.	Wald	df	Sig.	Exp(B)
Step 0	Constant	-3.408	.084	1641.164	1	.000	.033

Variables not in the Equation

			Score	df	Sig.
Step 0	Variables	Cluster modal	26.949	6	<.001
		Cluster modal(1)	1.373	1	.241
		Cluster modal(2)	.600	1	.439
		Cluster modal(3)	.002	1	.966
		Cluster modal(4)	3.624	1	.057
		Cluster modal(5)	14.462	1	<.001
		Cluster modal(6)	6.069	1	.014
		fodelselandmammapappa_ny(1)	3.447	1	.063
		utbildningmammapappa_ny(1)	39.953	1	<.001
		Birthyear_decades	78.326	5	<.001
		Birthyear_decades(1)	17.159	1	<.001
		Birthyear_decades(2)	1.462	1	.227
		Birthyear_decades(3)	3.509	1	.061
		Birthyear_decades(4)	37.220	1	<.001
		Birthyear_decades(5)	7.269	1	.007
	Overall Statistics		119.075	13	<.001

Block 1: Method = Enter

Omnibus Tests of Model Coefficients

		Chi-square	df	Sig.
Step 1	Step	137.703	13	<.001
	Block	137.703	13	<.001
	Model	137.703	13	<.001

Model Summary

Step	-2 Log likelihood	Cox & Snell R Square	Nagelkerke R Square
1	1154.155[a]	.030	.121

a. Estimation terminated at iteration number 20 because maximum iterations has been reached. Final solution cannot be found.

Classification Table[a]

	Observed		Predicted		
			Fibromyalgia_ny		Percentage Correct
			Nej	Ja	
Step 1	Fibromyalgia_ny	Nej	4409	0	100.0
		Ja	146	0	.0
	Overall Percentage				96.8

a. The cut value is ,500

Variables in the Equation

		B	S.E.	Wald	df	Sig.	Exp(B)	95% C.I.for EXP(B)	
								Lower	Upper
Step 1[a]	Cluster modal			36.362	6	<.001			
	Cluster modal(1)	.429	.251	2.923	1	.087	1.535	.939	2.509
	Cluster modal(2)	.045	.326	.019	1	.890	1.046	.553	1.980
	Cluster modal(3)	.538	.342	2.469	1	.116	1.713	.875	3.351
	Cluster modal(4)	.799	.323	6.113	1	.013	2.222	1.180	4.186
	Cluster modal(5)	1.533	.295	26.967	1	<.001	4.632	2.597	8.260
	Cluster modal(6)	1.265	.352	12.881	1	<.001	3.542	1.775	7.066
	fodelselandmammapappa_ny(1)	-.545	.520	1.098	1	.295	.580	.209	1.607
	utbildningmammapappa_ny(1)	.531	.201	6.944	1	.008	1.701	1.146	2.524
	Birthyear_decades			29.124	5	<.001			
	Birthyear_decades(1)	16.231	1477.569	.000	1	.991	11189986.858	.000	.
	Birthyear_decades(2)	17.287	1477.569	.000	1	.991	32194517.031	.000	.
	Birthyear_decades(3)	17.698	1477.569	.000	1	.990	48542172.941	.000	.
	Birthyear_decades(4)	18.234	1477.569	.000	1	.990	82935200.093	.000	.
	Birthyear_decades(5)	18.155	1477.569	.000	1	.990	76658927.690	.000	.
	Constant	-21.617	1477.569	.000	1	.988	.000		

a. Variable(s) entered on step 1: Cluster modal, fodelselandmammapappa_ny, utbildningmammapappa_ny, Birthyear_decades.

Logistic Regression

Notes

Output Created		26-AUG-2025 11:46:52
Comments		
Input	Data	/Users/stevenlc/Library/CloudStorage/OneDrive-Privat/ICloud filer/Doktorander/Rickard/Artikel 4/Rickardonlywomen7classmodel.sav
	Active Dataset	DataSet4
	File Label	Scored Data File
	Filter	<none>
	Weight	<none>
	Split File	<none>
	N of Rows in Working Data File	5681
Missing Value Handling	Definition of Missing	User-defined missing values are treated as missing
Syntax		LOGISTIC REGRESSION VARIABLES Ischemic_HD_ny<br>/METHOD=ENTER clu# fodelselandmammapappa_ny utbildningmammapappa_ny Birthyear_decades<br>/CONTRAST (clu#)=Indicator(1)<br>/CONTRAST (fodelselandmammapappa_ny)=Indicator(1)<br>/CONTRAST (utbildningmammapappa_ny)=Indicator(1)<br>/CONTRAST (Birthyear_decades)=Indicator(1)<br>/PRINT=CI(95)<br>/CRITERIA=PIN(0.05) POUT(0.10) ITERATE(20) CUT(0.5).
Resources	Processor Time	00:00:00,28
	Elapsed Time	00:00:00,00

Case Processing Summary

Unweighted Cases[a]		N	Percent
Selected Cases	Included in Analysis	4555	80.2
	Missing Cases	1126	19.8
	Total	5681	100.0
Unselected Cases		0	.0
Total		5681	100.0

a. If weight is in effect, see classification table for the total number of cases.

Dependent Variable Encoding

Original Value	Internal Value
Nej	0
Ja	1

Categorical Variables Codings

		Frequency	Parameter coding (1)	(2)	(3)	(4)	(5)	(6)
Cluster modal	1	2138	.000	.000	.000	.000	.000	.000
	2	957	1.000	.000	.000	.000	.000	.000
	3	461	.000	1.000	.000	.000	.000	.000
	4	339	.000	.000	1.000	.000	.000	.000
	5	246	.000	.000	.000	1.000	.000	.000
	6	244	.000	.000	.000	.000	1.000	.000
	7	170	.000	.000	.000	.000	.000	1.000
Birthyear_decades	18-27	717	.000	.000	.000	.000	.000	
	28-37	760	1.000	.000	.000	.000	.000	
	38-47	862	.000	1.000	.000	.000	.000	
	48-57	908	.000	.000	1.000	.000	.000	
	58-67	929	.000	.000	.000	1.000	.000	
	68-74	379	.000	.000	.000	.000	1.000	
utbildningmammapappa_ny	Minst en förälder högre utb än grundskola	2448	.000					
	Båda föräldrarna grundskola	2107	1.000					
fodelselandmammapappa_ny	Minst en förälder född i norden	4261	.000					
	Båda födda utanför Norden	294	1.000					

Block 0: Beginning Block

Classification Table[a,b]

Observed			Predicted: IHD_ny Nej	Predicted: IHD_ny Ja	Percentage Correct
Step 0	IHD_ny	Nej	4419	0	100.0
		Ja	136	0	.0
	Overall Percentage				97.0

a. Constant is included in the model.

b. The cut value is ,500

Variables in the Equation

		B	S.E.	Wald	df	Sig.	Exp(B)
Step 0	Constant	-3.481	.087	1598.770	1	.000	.031

Variables not in the Equation

			Score	df	Sig.
Step 0	Variables	Cluster modal	14.970	6	.020
		Cluster modal(1)	4.186	1	.041
		Cluster modal(2)	1.181	1	.277
		Cluster modal(3)	5.206	1	.023
		Cluster modal(4)	2.801	1	.094
		Cluster modal(5)	2.063	1	.151
		Cluster modal(6)	.781	1	.377
		fodelselandmammapappa_ny(1)	.187	1	.665
		utbildningmammapappa_ny(1)	44.235	1	<.001
		Birthyear_decades	131.268	5	<.001
		Birthyear_decades(1)	10.221	1	.001
		Birthyear_decades(2)	12.234	1	<.001
		Birthyear_decades(3)	1.773	1	.183
		Birthyear_decades(4)	27.481	1	<.001
		Birthyear_decades(5)	81.749	1	<.001
	Overall Statistics		158.435	13	<.001

Block 1: Method = Enter

Omnibus Tests of Model Coefficients

		Chi-square	df	Sig.
Step 1	Step	141.148	13	<.001
	Block	141.148	13	<.001
	Model	141.148	13	<.001

Model Summary

Step	-2 Log likelihood	Cox & Snell R Square	Nagelkerke R Square
1	1081.831[a]	.031	.130

a. Estimation terminated at iteration number 8 because parameter estimates changed by less than ,001.

Classification Table[a]

Observed			Predicted IHD_ny Nej	Predicted IHD_ny Ja	Percentage Correct
Step 1	IHD_ny	Nej	4419	0	100.0
		Ja	136	0	.0
	Overall Percentage				97.0

a. The cut value is ,500

Variables in the Equation

		B	S.E.	Wald	df	Sig.	Exp(B)	95% C.I.for EXP(B) Lower	95% C.I.for EXP(B) Upper
Step 1[a]	Cluster modal			22.757	6	<.001			
	Cluster modal(1)	.054	.271	.039	1	.843	1.055	.620	1.795
	Cluster modal(2)	-.309	.347	.790	1	.374	.734	.372	1.451
	Cluster modal(3)	.930	.291	10.220	1	.001	2.535	1.433	4.483
	Cluster modal(4)	-.716	.600	1.424	1	.233	.489	.151	1.584
	Cluster modal(5)	.988	.349	7.995	1	.005	2.686	1.354	5.326
	Cluster modal(6)	.725	.420	2.975	1	.085	2.065	.906	4.707
	fodelselandmammapappa_ny(1)	.657	.352	3.499	1	.061	1.930	.969	3.844
	utbildningmammapappa_ny(1)	.636	.223	8.161	1	.004	1.890	1.221	2.924
	Birthyear_decades			73.901	5	<.001			
	Birthyear_decades(1)	.607	.606	1.004	1	.316	1.835	.560	6.019
	Birthyear_decades(2)	.517	.600	.742	1	.389	1.677	.517	5.442
	Birthyear_decades(3)	1.137	.562	4.100	1	.043	3.118	1.037	9.373
	Birthyear_decades(4)	2.085	.542	14.800	1	<.001	8.043	2.781	23.266

Birthyear_decades(5)	2.796	.553	25.586	1	<.001	16.380	5.544	48.398
Constant	-5.537	.523	112.002	1	<.001	.004		

a. Variable(s) entered on step 1: Cluster modal, fodelselandmammapappa_ny, utbildningmammapappa_ny, Birthyear_decades.

Logistic Regression

Notes

Output Created		26-AUG-2025 11:46:52
Comments		
Input	Data	/Users/stevenlc/Library/CloudStorage/OneDrive-Privat/ICloud filer/Doktorander/Rickard/Artikel 4/Rickardonlywomen7classmodel.sav
	Active Dataset	DataSet4
	File Label	Scored Data File
	Filter	<none>
	Weight	<none>
	Split File	<none>
	N of Rows in Working Data File	5681
Missing Value Handling	Definition of Missing	User-defined missing values are treated as missing

Syntax		LOGISTIC REGRESSION VARIABLES KOL_ny /METHOD=ENTER clu# fodelselandmammapappa_ny utbildningmammapappa_ny Birthyear_decades /CONTRAST (clu#)=Indicator(1) /CONTRAST (fodelselandmammapappa_ny)=Indicator(1) /CONTRAST (utbildningmammapappa_ny)=Indicator(1) /CONTRAST (Birthyear_decades)=Indicator(1) /PRINT=CI(95) /CRITERIA=PIN(0.05) POUT(0.10) ITERATE(20) CUT(0.5).
Resources	Processor Time	00:00:00,28
	Elapsed Time	00:00:00,00

Case Processing Summary

Unweighted Cases[a]		N	Percent
Selected Cases	Included in Analysis	4555	80.2
	Missing Cases	1126	19.8
	Total	5681	100.0
Unselected Cases		0	.0
Total		5681	100.0

a. If weight is in effect, see classification table for the total number of cases.

Dependent Variable Encoding

Original Value	Internal Value
Nej	0
Ja	1

Categorical Variables Codings

	Frequency	Parameter coding

			(1)	(2)	(3)	(4)	(5)	(6)
Cluster modal	1	2138	.000	.000	.000	.000	.000	.000
	2	957	1.000	.000	.000	.000	.000	.000
	3	461	.000	1.000	.000	.000	.000	.000
	4	339	.000	.000	1.000	.000	.000	.000
	5	246	.000	.000	.000	1.000	.000	.000
	6	244	.000	.000	.000	.000	1.000	.000
	7	170	.000	.000	.000	.000	.000	1.000
Birthyear_decades	18-27	717	.000	.000	.000	.000	.000	
	28-37	760	1.000	.000	.000	.000	.000	
	38-47	862	.000	1.000	.000	.000	.000	
	48-57	908	.000	.000	1.000	.000	.000	
	58-67	929	.000	.000	.000	1.000	.000	
	68-74	379	.000	.000	.000	.000	1.000	
utbildningmammapappa_ny	Minst en förälder högre utb än grundskola	2448	.000					
	Båda föräldrarna grundskola	2107	1.000					
fodelselandmammapappa_ny	Minst en förälder född i norden	4261	.000					
	Båda födda utanför Norden	294	1.000					

Block 0: Beginning Block

Classification Table[a,b]

			Predicted		
			COPD_ny		Percentage Correct
	Observed		Nej	Ja	
Step 0	COPD_ny	Nej	4479	0	100.0
		Ja	76	0	.0
	Overall Percentage				98.3

a. Constant is included in the model.
b. The cut value is ,500

Variables in the Equation

		B	S.E.	Wald	df	Sig.	Exp(B)
Step 0	Constant	-4.076	.116	1241.837	1	<.001	.017

Variables not in the Equation

	Score	df	Sig.

Step 0	Variables	Cluster modal	22.392	6	.001
		Cluster modal(1)	2.871	1	.090
		Cluster modal(2)	.014	1	.906
		Cluster modal(3)	4.211	1	.040
		Cluster modal(4)	6.277	1	.012
		Cluster modal(5)	4.074	1	.044
		Cluster modal(6)	6.456	1	.011
		fodelselandmammapappa_ny(1)	.002	1	.964
		utbildningmammapappa_ny(1)	15.273	1	<.001
		Birthyear_decades	86.268	5	<.001
		Birthyear_decades(1)	10.981	1	<.001
		Birthyear_decades(2)	11.299	1	<.001
		Birthyear_decades(3)	.111	1	.739
		Birthyear_decades(4)	34.635	1	<.001
		Birthyear_decades(5)	32.811	1	<.001
	Overall Statistics		112.378	13	<.001

Block 1: Method = Enter

Omnibus Tests of Model Coefficients

		Chi-square	df	Sig.
Step 1	Step	109.540	13	<.001
	Block	109.540	13	<.001
	Model	109.540	13	<.001

Model Summary

Step	-2 Log likelihood	Cox & Snell R Square	Nagelkerke R Square
1	663.358[a]	.024	.152

a. Estimation terminated at iteration number 9 because parameter estimates changed by less than ,001.

Classification Table[a]

Observed			Predicted COPD_ny Nej	Predicted COPD_ny Ja	Predicted Percentage Correct
Step 1	COPD_ny	Nej	4479	0	100.0

	Ja	76	0	.0
	Overall Percentage			98.3

a. The cut value is ,500

Variables in the Equation

		B	S.E.	Wald	df	Sig.	Exp(B)	95% C.I.for EXP(B) Lower	Upper
Step 1[a]	Cluster modal			25.702	6	<.001			
	Cluster modal(1)	.167	.372	.203	1	.653	1.182	.570	2.451
	Cluster modal(2)	.197	.403	.240	1	.624	1.218	.553	2.681
	Cluster modal(3)	-1.258	1.021	1.518	1	.218	.284	.038	2.103
	Cluster modal(4)	1.135	.396	8.204	1	.004	3.110	1.431	6.762
	Cluster modal(5)	1.425	.422	11.408	1	<.001	4.157	1.819	9.503
	Cluster modal(6)	1.427	.446	10.227	1	.001	4.167	1.738	9.991
	fodelselandmammapappa_ny(1)	.478	.491	.949	1	.330	1.612	.617	4.217
	utbildningmammapappa_ny(1)	.097	.272	.127	1	.722	1.102	.647	1.876
	Birthyear_decades			52.023	5	<.001			
	Birthyear_decades(1)	.590	1.227	.231	1	.631	1.804	.163	19.988
	Birthyear_decades(2)	.796	1.160	.471	1	.492	2.217	.228	21.525
	Birthyear_decades(3)	2.265	1.046	4.684	1	.030	9.627	1.238	74.849
	Birthyear_decades(4)	3.367	1.030	10.696	1	.001	28.992	3.854	218.071
	Birthyear_decades(5)	3.803	1.046	13.231	1	<.001	44.855	5.778	348.219
	Constant	-6.938	1.020	46.304	1	<.001	.001		

a. Variable(s) entered on step 1: Cluster modal, fodelselandmammapappa_ny, utbildningmammapappa_ny, Birthyear_decades.

Logistic Regression

Notes

Output Created		26-AUG-2025 11:46:52
Comments		
Input	Data	/Users/stevenlc/Library/CloudStorage/OneDrive-Privat/ICloud filer/Doktorander/Rickard/Artikel 4/Rickardonlywomen7classmodel.sav
	Active Dataset	DataSet4

	File Label	Scored Data File
	Filter	<none>
	Weight	<none>
	Split File	<none>
	N of Rows in Working Data File	5681
Missing Value Handling	Definition of Missing	User-defined missing values are treated as missing
Syntax		LOGISTIC REGRESSION VARIABLES Diabetes_II_ny /METHOD=ENTER clu# fodelselandmammapappa_ny utbildningmammapappa_ny Birthyear_decades /CONTRAST (clu#)=Indicator(1) /CONTRAST (fodelselandmammapappa_ny)=Indicator(1) /CONTRAST (utbildningmammapappa_ny)=Indicator(1) /CONTRAST (Birthyear_decades)=Indicator(1) /PRINT=CI(95) /CRITERIA=PIN(0.05) POUT(0.10) ITERATE(20) CUT(0.5).
Resources	Processor Time	00:00:00,32
	Elapsed Time	00:00:01,00

Case Processing Summary

Unweighted Cases[a]		N	Percent
Selected Cases	Included in Analysis	4555	80.2
	Missing Cases	1126	19.8
	Total	5681	100.0
Unselected Cases		0	.0
Total		5681	100.0

a. If weight is in effect, see classification table for the total number of cases.

Dependent Variable Encoding

Original Value	Internal Value
Nej	0
Ja	1

Categorical Variables Codings

		Frequency	Parameter coding (1)	(2)	(3)	(4)	(5)	(6)
Cluster modal	1	2138	.000	.000	.000	.000	.000	.000
	2	957	1.000	.000	.000	.000	.000	.000
	3	461	.000	1.000	.000	.000	.000	.000
	4	339	.000	.000	1.000	.000	.000	.000
	5	246	.000	.000	.000	1.000	.000	.000
	6	244	.000	.000	.000	.000	1.000	.000
	7	170	.000	.000	.000	.000	.000	1.000
Birthyear_decades	18-27	717	.000	.000	.000	.000	.000	
	28-37	760	1.000	.000	.000	.000	.000	
	38-47	862	.000	1.000	.000	.000	.000	
	48-57	908	.000	.000	1.000	.000	.000	
	58-67	929	.000	.000	.000	1.000	.000	
	68-74	379	.000	.000	.000	.000	1.000	
utbildningmammapappa_ny	Minst en förälder högre utb än grundskola	2448	.000					
	Båda föräldrarna grundskola	2107	1.000					
fodelselandmammapappa_ny	Minst en förälder född i norden	4261	.000					
	Båda födda utanför Norden	294	1.000					

Block 0: Beginning Block

Classification Table[a,b]

Observed			Predicted: Diabetes type II_ny Nej	Ja	Percentage Correct
Step 0	Diabetes type II_ny	Nej	4444	0	100.0
		Ja	111	0	.0
	Overall Percentage				97.6

a. Constant is included in the model.

b. The cut value is ,500

Variables in the Equation

		B	S.E.	Wald	df	Sig.	Exp(B)
Step 0	Constant	-3.690	.096	1474.381	1	.000	.025

Variables not in the Equation

			Score	df	Sig.
Step 0	Variables	Cluster modal	17.132	6	.009
		Cluster modal(1)	7.131	1	.008
		Cluster modal(2)	1.820	1	.177
		Cluster modal(3)	.213	1	.644
		Cluster modal(4)	1.632	1	.201
		Cluster modal(5)	1.581	1	.209
		Cluster modal(6)	.336	1	.562
		fodelselandmammapappa_ny(1)	.207	1	.649
		utbildningmammapappa_ny(1)	28.408	1	<.001
		Birthyear_decades	117.658	5	<.001
		Birthyear_decades(1)	14.005	1	<.001
		Birthyear_decades(2)	4.881	1	.027
		Birthyear_decades(3)	.985	1	.321
		Birthyear_decades(4)	25.953	1	<.001
		Birthyear_decades(5)	68.362	1	<.001
	Overall Statistics		125.524	13	<.001

Block 1: Method = Enter

Omnibus Tests of Model Coefficients

		Chi-square	df	Sig.
Step 1	Step	120.963	13	<.001
	Block	120.963	13	<.001
	Model	120.963	13	<.001

Model Summary

Step	-2 Log likelihood	Cox & Snell R Square	Nagelkerke R Square
1	922.918[a]	.026	.128

a. Estimation terminated at iteration number 20 because maximum iterations has been reached. Final solution cannot be found.

Classification Table[a]

	Observed		Predicted		
			Diabetes type II_ny		Percentage Correct
			Nej	Ja	
Step 1	Diabetes type II_ny	Nej	4444	0	100.0
		Ja	111	0	.0
	Overall Percentage				97.6

a. The cut value is ,500

Variables in the Equation

		B	S.E.	Wald	df	Sig.	Exp(B)	95% C.I.for EXP(B)	
								Lower	Upper
Step 1[a]	Cluster modal			7.025	6	.319			
	Cluster modal(1)	-.488	.322	2.296	1	.130	.614	.327	1.154
	Cluster modal(2)	-.716	.404	3.151	1	.076	.488	.221	1.077
	Cluster modal(3)	-.095	.409	.054	1	.817	.909	.408	2.029
	Cluster modal(4)	.318	.370	.737	1	.390	1.374	.665	2.839
	Cluster modal(5)	-.516	.603	.734	1	.391	.597	.183	1.944
	Cluster modal(6)	-.290	.605	.230	1	.632	.748	.229	2.449
	fodelselandmammapappa_ny(1)	.337	.439	.592	1	.442	1.401	.593	3.309
	utbildningmammapappa_ny(1)	.160	.231	.480	1	.489	1.173	.746	1.845
	Birthyear_decades			48.809	5	<.001			
	Birthyear_decades(1)	15.906	1492.917	.000	1	.991	8089429.055	.000	.
	Birthyear_decades(2)	16.847	1492.917	.000	1	.991	20722410.325	.000	.
	Birthyear_decades(3)	17.170	1492.917	.000	1	.991	28633137.474	.000	.
	Birthyear_decades(4)	18.023	1492.917	.000	1	.990	67178970.935	.000	.
	Birthyear_decades(5)	18.659	1492.917	.000	1	.990	126966516.407	.000	.
	Constant	-21.032	1492.917	.000	1	.989	.000		

a. Variable(s) entered on step 1: Cluster modal, fodelselandmammapappa_ny, utbildningmammapappa_ny, Birthyear_decades.

Logistic Regression

Notes

Output Created		26-AUG-2025 11:46:53
Comments		
Input	Data	/Users/stevenlc/Library/CloudStorage/OneDrive-Privat/ICloud filer/Doktorander/Rickard/Artikel 4/Rickardonlywomen7classmodel.sav
	Active Dataset	DataSet4
	File Label	Scored Data File
	Filter	<none>
	Weight	<none>
	Split File	<none>
	N of Rows in Working Data File	5681
Missing Value Handling	Definition of Missing	User-defined missing values are treated as missing
Syntax		LOGISTIC REGRESSION VARIABLES Tumörsjd_ny /METHOD=ENTER clu# fodelselandmammapappa_ny utbildningmammapappa_ny Birthyear_decades /CONTRAST (clu#)=Indicator(1) /CONTRAST (fodelselandmammapappa_ny)=Indicator(1) /CONTRAST (utbildningmammapappa_ny)=Indicator(1) /CONTRAST (Birthyear_decades)=Indicator(1) /PRINT=CI(95) /CRITERIA=PIN(0.05) POUT(0.10) ITERATE(20) CUT(0.5).
Resources	Processor Time	00:00:00,28
	Elapsed Time	00:00:00,00

Case Processing Summary

Unweighted Cases[a]		N	Percent
Selected Cases	Included in Analysis	4555	80.2
	Missing Cases	1126	19.8
	Total	5681	100.0
Unselected Cases		0	.0
Total		5681	100.0

a. If weight is in effect, see classification table for the total number of cases.

Dependent Variable Encoding

Original Value	Internal Value
Nej	0
Ja	1

Categorical Variables Codings

		Frequency	Parameter coding (1)	(2)	(3)	(4)	(5)	(6)
Cluster modal	1	2138	.000	.000	.000	.000	.000	.000
	2	957	1.000	.000	.000	.000	.000	.000
	3	461	.000	1.000	.000	.000	.000	.000
	4	339	.000	.000	1.000	.000	.000	.000
	5	246	.000	.000	.000	1.000	.000	.000
	6	244	.000	.000	.000	.000	1.000	.000
	7	170	.000	.000	.000	.000	.000	1.000
Birthyear_decades	18-27	717	.000	.000	.000	.000	.000	
	28-37	760	1.000	.000	.000	.000	.000	
	38-47	862	.000	1.000	.000	.000	.000	
	48-57	908	.000	.000	1.000	.000	.000	
	58-67	929	.000	.000	.000	1.000	.000	
	68-74	379	.000	.000	.000	.000	1.000	
utbildningmammapappa_ny	Minst en förälder högre utb än grundskola	2448	.000					
	Båda föräldrarna grundskola	2107	1.000					
fodelselandmammapappa_ny	Minst en förälder född i norden	4261	.000					
	Båda födda utanför Norden	294	1.000					

Block 0: Beginning Block

Classification Table[a,b]

Observed			Predicted: Cancer_ny Nej	Predicted: Cancer_ny Ja	Predicted: Percentage Correct
Step 0	Cancer_ny	Nej	4335	0	100.0
		Ja	220	0	.0
	Overall Percentage				95.2

a. Constant is included in the model.

b. The cut value is ,500

Variables in the Equation

		B	S.E.	Wald	df	Sig.	Exp(B)
Step 0	Constant	-2.981	.069	1860.388	1	.000	.051

Variables not in the Equation

			Score	df	Sig.
Step 0	Variables	Cluster modal	2.976	6	.812
		Cluster modal(1)	1.114	1	.291
		Cluster modal(2)	.004	1	.951
		Cluster modal(3)	.478	1	.489
		Cluster modal(4)	.331	1	.565
		Cluster modal(5)	.974	1	.324
		Cluster modal(6)	.426	1	.514
		fodelselandmammapappa_ny(1)	.810	1	.368
		utbildningmammapappa_ny(1)	41.069	1	<.001
		Birthyear_decades	135.274	5	<.001
		Birthyear_decades(1)	20.973	1	<.001
		Birthyear_decades(2)	10.808	1	.001
		Birthyear_decades(3)	3.717	1	.054
		Birthyear_decades(4)	45.045	1	<.001
		Birthyear_decades(5)	41.338	1	<.001
	Overall Statistics		143.929	13	<.001

Block 1: Method = Enter

Omnibus Tests of Model Coefficients

		Chi-square	df	Sig.
Step 1	Step	157.853	13	<.001
	Block	157.853	13	<.001
	Model	157.853	13	<.001

Model Summary

Step	-2 Log likelihood	Cox & Snell R Square	Nagelkerke R Square
1	1604.702[a]	.034	.106

a. Estimation terminated at iteration number 9 because parameter estimates changed by less than ,001.

Classification Table[a]

Observed			Predicted		
			Cancer_ny		Percentage Correct
			Nej	Ja	
Step 1	Cancer_ny	Nej	4335	0	100.0
		Ja	220	0	.0
	Overall Percentage				95.2

a. The cut value is ,500

Variables in the Equation

		B	S.E.	Wald	df	Sig.	Exp(B)	95% C.I.for EXP(B)	
								Lower	Upper
Step 1[a]	Cluster modal			8.994	6	.174			
	Cluster modal(1)	.267	.198	1.827	1	.177	1.306	.887	1.925
	Cluster modal(2)	.029	.244	.014	1	.907	1.029	.637	1.662
	Cluster modal(3)	.460	.265	3.017	1	.082	1.584	.943	2.662
	Cluster modal(4)	-.107	.344	.098	1	.755	.898	.458	1.762
	Cluster modal(5)	.655	.296	4.883	1	.027	1.925	1.077	3.440
	Cluster modal(6)	.462	.351	1.729	1	.188	1.587	.797	3.158
	fodelselandmammapappa_ny(1)	.090	.326	.077	1	.782	1.094	.578	2.073
	utbildningmammapappa_ny(1)	.196	.162	1.465	1	.226	1.216	.886	1.669
	Birthyear_decades			79.242	5	<.001			
	Birthyear_decades(1)	1.706	.766	4.956	1	.026	5.506	1.226	24.721
	Birthyear_decades(2)	2.220	.741	8.971	1	.003	9.210	2.154	39.376
	Birthyear_decades(3)	3.051	.727	17.612	1	<.001	21.142	5.085	87.907
	Birthyear_decades(4)	3.522	.725	23.566	1	<.001	33.836	8.164	140.239

	Birthyear_decades(5)	3.821	.736	26.958	1	<.001	45.633	10.787	193.035
	Constant	-6.089	.716	72.352	1	<.001	.002		

a. Variable(s) entered on step 1: Cluster modal, fodelselandmammapappa_ny, utbildningmammapappa_ny, Birthyear_decades.

Logistic Regression

Notes

Output Created		26-AUG-2025 11:46:53
Comments		
Input	Data	/Users/stevenlc/Library/CloudStorage/OneDrive-Privat/ICloud filer/Doktorander/Rickard/Artikel 4/Rickardonlywomen7classmodel.sav
	Active Dataset	DataSet4
	File Label	Scored Data File
	Filter	<none>
	Weight	<none>
	Split File	<none>
	N of Rows in Working Data File	5681
Missing Value Handling	Definition of Missing	User-defined missing values are treated as missing

Syntax		LOGISTIC REGRESSION VARIABLES Fetma_ny<br>/METHOD=ENTER clu# fodelselandmammapappa_ny utbildningmammapappa_ny Birthyear_decades<br>/CONTRAST (clu#)=Indicator(1)<br>/CONTRAST (fodelselandmammapappa_ny)=Indicator(1)<br>/CONTRAST (utbildningmammapappa_ny)=Indicator(1)<br>/CONTRAST (Birthyear_decades)=Indicator(1)<br>/PRINT=CI(95)<br>/CRITERIA=PIN(0.05) POUT(0.10) ITERATE(20) CUT(0.5).
Resources	Processor Time	00:00:00,26
	Elapsed Time	00:00:00,00

Case Processing Summary

Unweighted Cases[a]		N	Percent
Selected Cases	Included in Analysis	4471	78.7
	Missing Cases	1210	21.3
	Total	5681	100.0
Unselected Cases		0	.0
Total		5681	100.0

a. If weight is in effect, see classification table for the total number of cases.

Dependent Variable Encoding

Original Value	Internal Value
Nej	0
Ja	1

Categorical Variables Codings

	Frequency	Parameter coding

			(1)	(2)	(3)	(4)	(5)	(6)
Cluster modal	1	2094	.000	.000	.000	.000	.000	.000
	2	941	1.000	.000	.000	.000	.000	.000
	3	455	.000	1.000	.000	.000	.000	.000
	4	331	.000	.000	1.000	.000	.000	.000
	5	244	.000	.000	.000	1.000	.000	.000
	6	241	.000	.000	.000	.000	1.000	.000
	7	165	.000	.000	.000	.000	.000	1.000
Birthyear_decades	18-27	702	.000	.000	.000	.000	.000	
	28-37	757	1.000	.000	.000	.000	.000	
	38-47	849	.000	1.000	.000	.000	.000	
	48-57	891	.000	.000	1.000	.000	.000	
	58-67	903	.000	.000	.000	1.000	.000	
	68-74	369	.000	.000	.000	.000	1.000	
utbildningmammapappa_ny	Minst en förälder högre utb än grundskola	2412	.000					
	Båda föräldrarna grundskola	2059	1.000					
fodelselandmammapappa_ny	Minst en förälder född i norden	4185	.000					
	Båda födda utanför Norden	286	1.000					

Block 0: Beginning Block

Classification Table[a,b]

Observed			Predicted Obesity_ny Nej	Predicted Obesity_ny Ja	Percentage Correct
Step 0	Obesity_ny	Nej	3923	0	100.0
		Ja	548	0	.0
	Overall Percentage				87.7

a. Constant is included in the model.

b. The cut value is ,500

Variables in the Equation

		B	S.E.	Wald	df	Sig.	Exp(B)
Step 0	Constant	-1.968	.046	1862.915	1	.000	.140

Variables not in the Equation

	Score	df	Sig.

Step 0	Variables	Cluster modal	15.539	6	.016
		Cluster modal(1)	5.697	1	.017
		Cluster modal(2)	1.373	1	.241
		Cluster modal(3)	4.687	1	.030
		Cluster modal(4)	.341	1	.560
		Cluster modal(5)	1.702	1	.192
		Cluster modal(6)	3.538	1	.060
		fodelselandmammapappa_ny(1)	.039	1	.844
		utbildningmammapappa_ny(1)	44.162	1	<.001
		Birthyear_decades	38.291	5	<.001
		Birthyear_decades(1)	2.809	1	.094
		Birthyear_decades(2)	4.351	1	.037
		Birthyear_decades(3)	11.568	1	<.001
		Birthyear_decades(4)	.023	1	.881
		Birthyear_decades(5)	.915	1	.339
	Overall Statistics		78.698	13	<.001

Block 1: Method = Enter

Omnibus Tests of Model Coefficients

		Chi-square	df	Sig.
Step 1	Step	80.986	13	<.001
	Block	80.986	13	<.001
	Model	80.986	13	<.001

Model Summary

Step	-2 Log likelihood	Cox & Snell R Square	Nagelkerke R Square
1	3245.526[a]	.018	.034

a. Estimation terminated at iteration number 5 because parameter estimates changed by less than ,001.

Classification Table[a]

Observed			Predicted: Obesity_ny Nej	Predicted: Obesity_ny Ja	Percentage Correct
Step 1	Obesity_ny	Nej	3923	0	100.0

Ja	548	0	.0
Overall Percentage			87.7

a. The cut value is ,500

Variables in the Equation

		B	S.E.	Wald	df	Sig.	Exp(B)	95% C.I.for EXP(B) Lower	Upper
Step 1[a]	Cluster modal			14.836	6	.022			
	Cluster modal(1)	-.151	.130	1.345	1	.246	.860	.666	1.110
	Cluster modal(2)	-.191	.168	1.294	1	.255	.826	.595	1.148
	Cluster modal(3)	.336	.167	4.062	1	.044	1.399	1.009	1.940
	Cluster modal(4)	-.180	.217	.689	1	.407	.835	.546	1.278
	Cluster modal(5)	.263	.196	1.804	1	.179	1.301	.886	1.911
	Cluster modal(6)	.408	.221	3.409	1	.065	1.504	.975	2.320
	fodelselandmammapappa_ny(1)	.066	.192	.119	1	.730	1.069	.733	1.558
	utbildningmammapappa_ny(1)	.532	.106	25.277	1	<.001	1.703	1.384	2.095
	Birthyear_decades			19.993	5	.001			
	Birthyear_decades(1)	.437	.196	4.967	1	.026	1.549	1.054	2.275
	Birthyear_decades(2)	.703	.187	14.094	1	<.001	2.020	1.399	2.916
	Birthyear_decades(3)	.693	.190	13.280	1	<.001	2.000	1.378	2.905
	Birthyear_decades(4)	.378	.199	3.614	1	.057	1.459	.988	2.155
	Birthyear_decades(5)	.479	.231	4.312	1	.038	1.615	1.027	2.538
	Constant	-2.740	.166	272.965	1	<.001	.065		

a. Variable(s) entered on step 1: Cluster modal, fodelselandmammapappa_ny, utbildningmammapappa_ny, Birthyear_decades.

Logistic Regression

Notes

Output Created		26-AUG-2025 11:46:53
Comments		
Input	Data	/Users/stevenlc/Library/CloudStorage/OneDrive-Privat/ICloud filer/Doktorander/Rickard/Artikel 4/Rickardonlywomen7classmodel.sav
	Active Dataset	DataSet4

	File Label	Scored Data File
	Filter	<none>
	Weight	<none>
	Split File	<none>
	N of Rows in Working Data File	5681
Missing Value Handling	Definition of Missing	User-defined missing values are treated as missing
Syntax		LOGISTIC REGRESSION VARIABLES Storrökare_ny /METHOD=ENTER clu# fodelselandmammapappa_ny utbildningmammapappa_ny Birthyear_decades /CONTRAST (clu#)=Indicator(1) /CONTRAST (fodelselandmammapappa_ny)=Indicator(1) /CONTRAST (utbildningmammapappa_ny)=Indicator(1) /CONTRAST (Birthyear_decades)=Indicator(1) /PRINT=CI(95) /CRITERIA=PIN(0.05) POUT(0.10) ITERATE(20) CUT(0.5).
Resources	Processor Time	00:00:00,27
	Elapsed Time	00:00:00,00

Case Processing Summary

Unweighted Cases[a]		N	Percent
Selected Cases	Included in Analysis	4509	79.4
	Missing Cases	1172	20.6
	Total	5681	100.0
Unselected Cases		0	.0
Total		5681	100.0

a. If weight is in effect, see classification table for the total number of cases.

Dependent Variable Encoding

Original Value	Internal Value
Nej	0
Ja	1

Categorical Variables Codings

		Frequency	Parameter coding (1)	(2)	(3)	(4)	(5)	(6)
Cluster modal	1	2120	.000	.000	.000	.000	.000	.000
	2	947	1.000	.000	.000	.000	.000	.000
	3	456	.000	1.000	.000	.000	.000	.000
	4	336	.000	.000	1.000	.000	.000	.000
	5	244	.000	.000	.000	1.000	.000	.000
	6	242	.000	.000	.000	.000	1.000	.000
	7	164	.000	.000	.000	.000	.000	1.000
Birthyear_decades	18-27	714	.000	.000	.000	.000	.000	
	28-37	757	1.000	.000	.000	.000	.000	
	38-47	856	.000	1.000	.000	.000	.000	
	48-57	898	.000	.000	1.000	.000	.000	
	58-67	909	.000	.000	.000	1.000	.000	
	68-74	375	.000	.000	.000	.000	1.000	
utbildningmammapappa_ny	Minst en förälder högre utb än grundskola	2431	.000					
	Båda föräldrarna grundskola	2078	1.000					
fodelselandmammapappa_ny	Minst en förälder född i norden	4218	.000					
	Båda födda utanför Norden	291	1.000					

Block 0: Beginning Block

Classification Table[a,b]

Observed			Predicted Heavy smoking_ny Nej	Ja	Percentage Correct
Step 0	Heavy smoking_ny	Nej	4383	0	100.0
		Ja	126	0	.0
	Overall Percentage				97.2

a. Constant is included in the model.

b. The cut value is ,500

Variables in the Equation

		B	S.E.	Wald	df	Sig.	Exp(B)
Step 0	Constant	-3.549	.090	1542.852	1	.000	.029

Variables not in the Equation

			Score	df	Sig.
Step 0	Variables	Cluster modal	37.961	6	<.001
		Cluster modal(1)	5.387	1	.020
		Cluster modal(2)	.142	1	.706
		Cluster modal(3)	10.935	1	<.001
		Cluster modal(4)	6.095	1	.014
		Cluster modal(5)	.805	1	.370
		Cluster modal(6)	12.816	1	<.001
		fodelselandmammapappa_ny(1)	1.113	1	.292
		utbildningmammapappa_ny(1)	7.327	1	.007
		Birthyear_decades	38.852	5	<.001
		Birthyear_decades(1)	6.025	1	.014
		Birthyear_decades(2)	3.330	1	.068
		Birthyear_decades(3)	22.374	1	<.001
		Birthyear_decades(4)	5.698	1	.017
		Birthyear_decades(5)	.025	1	.875
	Overall Statistics		76.055	13	<.001

Block 1: Method = Enter

Omnibus Tests of Model Coefficients

		Chi-square	df	Sig.
Step 1	Step	70.694	13	<.001
	Block	70.694	13	<.001
	Model	70.694	13	<.001

Model Summary

Step	-2 Log likelihood	Cox & Snell R Square	Nagelkerke R Square

1	1079.294[a]	.016	.069

a. Estimation terminated at iteration number 7 because parameter estimates changed by less than ,001.

Classification Table[a]

Observed			Predicted: Heavy smoking_ny Nej	Ja	Percentage Correct
Step 1	Heavy smoking_ny	Nej	4383	0	100.0
		Ja	126	0	.0
	Overall Percentage				97.2

a. The cut value is ,500

Variables in the Equation

		B	S.E.	Wald	df	Sig.	Exp(B)	95% C.I.for EXP(B) Lower	Upper
Step 1[a]	Cluster modal			31.708	6	<.001			
	Cluster modal(1)	.001	.300	.000	1	.998	1.001	.555	1.804
	Cluster modal(2)	.398	.314	1.609	1	.205	1.489	.805	2.754
	Cluster modal(3)	1.152	.288	15.992	1	<.001	3.164	1.799	5.565
	Cluster modal(4)	.913	.329	7.714	1	.005	2.493	1.308	4.748
	Cluster modal(5)	.743	.381	3.810	1	.051	2.102	.997	4.431
	Cluster modal(6)	1.371	.345	15.809	1	<.001	3.940	2.004	7.746
	fodelselandmammapappa_ny(1)	.424	.332	1.629	1	.202	1.527	.797	2.928
	utbildningmammapappa_ny(1)	.149	.203	.539	1	.463	1.161	.779	1.729
	Birthyear_decades			27.151	5	<.001			
	Birthyear_decades(1)	.310	.489	.403	1	.525	1.364	.523	3.554
	Birthyear_decades(2)	.544	.462	1.390	1	.238	1.723	.697	4.259
	Birthyear_decades(3)	1.545	.423	13.337	1	<.001	4.689	2.046	10.746
	Birthyear_decades(4)	1.364	.436	9.770	1	.002	3.912	1.663	9.202
	Birthyear_decades(5)	1.084	.518	4.389	1	.036	2.958	1.072	8.159
	Constant	-5.014	.409	150.413	1	<.001	.007		

a. Variable(s) entered on step 1: Cluster modal, fodelselandmammapappa_ny, utbildningmammapappa_ny, Birthyear_decades.

Logistic Regression

Notes

Output Created		26-AUG-2025 11:46:53
Comments		
Input	Data	/Users/stevenlc/Library/CloudStorage/OneDrive-Privat/ICloud filer/Doktorander/Rickard/Artikel 4/Rickardonlywomen7classmodel.sav
	Active Dataset	DataSet4
	File Label	Scored Data File
	Filter	<none>
	Weight	<none>
	Split File	<none>
	N of Rows in Working Data File	5681
Missing Value Handling	Definition of Missing	User-defined missing values are treated as missing
Syntax		LOGISTIC REGRESSION VARIABLES AUDITRISK_inknykt_ny /METHOD=ENTER clu# fodelselandmammapappa_ny utbildningmammapappa_ny Birthyear_decades /CONTRAST (clu#)=Indicator(1) /CONTRAST (fodelselandmammapappa_ny)=Indicator(1) /CONTRAST (utbildningmammapappa_ny)=Indicator(1) /CONTRAST (Birthyear_decades)=Indicator(1) /PRINT=CI(95) /CRITERIA=PIN(0.05) POUT(0.10) ITERATE(20) CUT(0.5).
Resources	Processor Time	00:00:00,26
	Elapsed Time	00:00:01,00

Case Processing Summary

Unweighted Cases[a]		N	Percent
Selected Cases	Included in Analysis	4423	77.9
	Missing Cases	1258	22.1
	Total	5681	100.0
Unselected Cases		0	.0
Total		5681	100.0

a. If weight is in effect, see classification table for the total number of cases.

Dependent Variable Encoding

Original Value	Internal Value
Nej	0
Ja	1

Categorical Variables Codings

			Parameter coding					
		Frequency	(1)	(2)	(3)	(4)	(5)	(6)
Cluster modal	1	2075	.000	.000	.000	.000	.000	.000
	2	930	1.000	.000	.000	.000	.000	.000
	3	452	.000	1.000	.000	.000	.000	.000
	4	327	.000	.000	1.000	.000	.000	.000
	5	240	.000	.000	.000	1.000	.000	.000
	6	233	.000	.000	.000	.000	1.000	.000
	7	166	.000	.000	.000	.000	.000	1.000
Birthyear_decades	18-27	700	.000	.000	.000	.000	.000	
	28-37	745	1.000	.000	.000	.000	.000	
	38-47	842	.000	1.000	.000	.000	.000	
	48-57	876	.000	.000	1.000	.000	.000	
	58-67	899	.000	.000	.000	1.000	.000	
	68-74	361	.000	.000	.000	.000	1.000	
utbildningmammapappa_ny	Minst en förälder högre utb än grundskola	2394	.000					
	Båda föräldrarna grundskola	2029	1.000					
fodelselandmammapappa_ny	Minst en förälder född i norden	4137	.000					
	Båda födda utanför Norden	286	1.000					

Block 0: Beginning Block

Classification Table[a,b]

Observed			Predicted: Heavy drinking_ny Nej	Predicted: Heavy drinking_ny Ja	Predicted: Percentage Correct
Step 0	Heavy drinking_ny	Nej	3672	0	100.0
		Ja	751	0	.0
	Overall Percentage				83.0

a. Constant is included in the model.

b. The cut value is ,500

Variables in the Equation

		B	S.E.	Wald	df	Sig.	Exp(B)
Step 0	Constant	-1.587	.040	1570.459	1	.000	.205

Variables not in the Equation

			Score	df	Sig.
Step 0	Variables	Cluster modal	111.386	6	<.001
		Cluster modal(1)	13.288	1	<.001
		Cluster modal(2)	.245	1	.620
		Cluster modal(3)	9.823	1	.002
		Cluster modal(4)	.049	1	.825
		Cluster modal(5)	10.926	1	<.001
		Cluster modal(6)	44.940	1	<.001
		fodelselandmammapappa_ny(1)	14.721	1	<.001
		utbildningmammapappa_ny(1)	69.213	1	<.001
		Birthyear_decades	303.059	5	<.001
		Birthyear_decades(1)	.827	1	.363
		Birthyear_decades(2)	8.732	1	.003
		Birthyear_decades(3)	4.344	1	.037
		Birthyear_decades(4)	31.779	1	<.001
		Birthyear_decades(5)	31.381	1	<.001
	Overall Statistics		417.860	13	<.001

Block 1: Method = Enter

Omnibus Tests of Model Coefficients

		Chi-square	df	Sig.
Step 1	Step	381.801	13	<.001
	Block	381.801	13	<.001
	Model	381.801	13	<.001

Model Summary

Step	-2 Log likelihood	Cox & Snell R Square	Nagelkerke R Square
1	3648.082[a]	.083	.138

a. Estimation terminated at iteration number 5 because parameter estimates changed by less than ,001.

Classification Table[a]

Observed			Predicted: Heavy drinking_ny Nej	Predicted: Heavy drinking_ny Ja	Predicted: Percentage Correct
Step 1	Heavy drinking_ny	Nej	3621	51	98.6
		Ja	706	45	6.0
	Overall Percentage				82.9

a. The cut value is ,500

Variables in the Equation

		B	S.E.	Wald	df	Sig.	Exp(B)	95% C.I.for EXP(B) Lower	95% C.I.for EXP(B) Upper
Step 1[a]	Cluster modal			90.033	6	<.001			
	Cluster modal(1)	.486	.111	19.234	1	<.001	1.626	1.308	2.020
	Cluster modal(2)	.386	.150	6.607	1	.010	1.472	1.096	1.976
	Cluster modal(3)	.812	.154	27.668	1	<.001	2.252	1.664	3.048
	Cluster modal(4)	.576	.189	9.303	1	.002	1.780	1.229	2.577
	Cluster modal(5)	.857	.174	24.258	1	<.001	2.357	1.676	3.315
	Cluster modal(6)	1.470	.183	64.199	1	<.001	4.348	3.035	6.229
	fodelselandmammapappa_ny(1)	-1.030	.221	21.780	1	<.001	.357	.232	.550
	utbildningmammapappa_ny(1)	-.135	.102	1.734	1	.188	.874	.715	1.068
	Birthyear_decades			199.072	5	<.001			
	Birthyear_decades(1)	-1.243	.130	91.030	1	<.001	.289	.224	.372
	Birthyear_decades(2)	-1.433	.134	114.604	1	<.001	.239	.183	.310
	Birthyear_decades(3)	-1.336	.137	95.358	1	<.001	.263	.201	.344
	Birthyear_decades(4)	-1.586	.151	110.874	1	<.001	.205	.152	.275
	Birthyear_decades(5)	-2.054	.242	72.134	1	<.001	.128	.080	.206
	Constant	-.738	.098	57.244	1	<.001	.478		

a. Variable(s) entered on step 1: Cluster modal, fodelselandmammapappa_ny, utbildningmammapappa_ny, Birthyear_decades.

Logistic Regression

Notes

Output Created		26-AUG-2025 11:46:54
Comments		
Input	Data	/Users/stevenlc/Library/CloudStorage/OneDrive-Privat/ICloud filer/Doktorander/Rickard/Artikel 4/Rickardonlywomen7classmodel.sav
	Active Dataset	DataSet4
	File Label	Scored Data File
	Filter	<none>
	Weight	<none>
	Split File	<none>
	N of Rows in Working Data File	5681
Missing Value Handling	Definition of Missing	User-defined missing values are treated as missing

Syntax		LOGISTIC REGRESSION VARIABLES Drogmissbruk_ny /METHOD=ENTER clu# fodelselandmammapappa_ny utbildningmammapappa_ny Birthyear_decades /CONTRAST (clu#)=Indicator(1) /CONTRAST (fodelselandmammapappa_ny)=Indicator(1) /CONTRAST (utbildningmammapappa_ny)=Indicator(1) /CONTRAST (Birthyear_decades)=Indicator(1) /PRINT=CI(95) /CRITERIA=PIN(0.05) POUT(0.10) ITERATE(20) CUT(0.5).
Resources	Processor Time	00:00:00,33
	Elapsed Time	00:00:00,00

Case Processing Summary

Unweighted Cases[a]		N	Percent
Selected Cases	Included in Analysis	4555	80.2
	Missing Cases	1126	19.8
	Total	5681	100.0
Unselected Cases		0	.0
Total		5681	100.0

a. If weight is in effect, see classification table for the total number of cases.

Dependent Variable Encoding

Original Value	Internal Value
Nej	0
Ja	1

Categorical Variables Codings

		Frequency	Parameter coding (1)	(2)	(3)	(4)	(5)	(6)
Cluster modal	1	2138	.000	.000	.000	.000	.000	.000
	2	957	1.000	.000	.000	.000	.000	.000
	3	461	.000	1.000	.000	.000	.000	.000
	4	339	.000	.000	1.000	.000	.000	.000
	5	246	.000	.000	.000	1.000	.000	.000
	6	244	.000	.000	.000	.000	1.000	.000
	7	170	.000	.000	.000	.000	.000	1.000
Birthyear_decades	18-27	717	.000	.000	.000	.000	.000	
	28-37	760	1.000	.000	.000	.000	.000	
	38-47	862	.000	1.000	.000	.000	.000	
	48-57	908	.000	.000	1.000	.000	.000	
	58-67	929	.000	.000	.000	1.000	.000	
	68-74	379	.000	.000	.000	.000	1.000	
utbildningmammapappa_ny	Minst en förälder högre utb än grundskola	2448	.000					
	Båda föräldrarna grundskola	2107	1.000					
fodelselandmammapappa_ny	Minst en förälder född i norden	4261	.000					
	Båda födda utanför Norden	294	1.000					

Block 0: Beginning Block

Classification Table[a,b]

Observed			Predicted: Drug abuse_ny Nej	Ja	Percentage Correct
Step 0	Drug abuse_ny	Nej	4541	0	100.0
		Ja	14	0	.0
	Overall Percentage				99.7

a. Constant is included in the model.

b. The cut value is ,500

Variables in the Equation

		B	S.E.	Wald	df	Sig.	Exp(B)
Step 0	Constant	-5.782	.268	466.578	1	<.001	.003

Variables not in the Equation

			Score	df	Sig.
Step 0	Variables	Cluster modal	33.651	6	<.001
		Cluster modal(1)	.383	1	.536
		Cluster modal(2)	1.581	1	.209
		Cluster modal(3)	.002	1	.966
		Cluster modal(4)	7.061	1	.008
		Cluster modal(5)	.088	1	.766
		Cluster modal(6)	24.116	1	<.001
		fodelselandmammapappa_ny(1)	.011	1	.916
		utbildningmammapappa_ny(1)	.628	1	.428
		Birthyear_decades	6.397	5	.269
		Birthyear_decades(1)	.227	1	.634
		Birthyear_decades(2)	.057	1	.811
		Birthyear_decades(3)	.281	1	.596
		Birthyear_decades(4)	1.519	1	.218
		Birthyear_decades(5)	1.275	1	.259
	Overall Statistics		40.746	13	<.001

Block 1: Method = Enter

Omnibus Tests of Model Coefficients

		Chi-square	df	Sig.
Step 1	Step	26.322	13	.015
	Block	26.322	13	.015
	Model	26.322	13	.015

Model Summary

Step	-2 Log likelihood	Cox & Snell R Square	Nagelkerke R Square
1	163.613[a]	.006	.141

a. Estimation terminated at iteration number 20 because maximum iterations has been reached. Final solution cannot be found.

Classification Table[a]

Observed	Predicted	
	Drug abuse_ny	Percentage

			Nej	Ja	Correct
Step 1	Drug abuse_ny	Nej	4541	0	100.0
		Ja	14	0	.0
	Overall Percentage				99.7

a. The cut value is ,500

Variables in the Equation

		B	S.E.	Wald	df	Sig.	Exp(B)	95% C.I.for EXP(B)	
								Lower	Upper
Step 1[a]	Cluster modal			18.737	6	.005			
	Cluster modal(1)	.129	.918	.020	1	.889	1.137	.188	6.878
	Cluster modal(2)	-14.584	1804.062	.000	1	.994	.000	.000	.
	Cluster modal(3)	.584	1.159	.254	1	.614	1.794	.185	17.390
	Cluster modal(4)	2.246	.828	7.362	1	.007	9.447	1.865	47.842
	Cluster modal(5)	.918	1.160	.625	1	.429	2.504	.257	24.343
	Cluster modal(6)	2.744	.772	12.626	1	<.001	15.556	3.423	70.688
	fodelselandmammapappa_ny(1)	-.210	1.054	.040	1	.842	.811	.103	6.393
	utbildningmammapappa_ny (1)	.332	.621	.285	1	.593	1.393	.413	4.705
	Birthyear_decades			5.266	5	.384			
	Birthyear_decades(1)	-.689	.743	.861	1	.353	.502	.117	2.152
	Birthyear_decades(2)	-1.010	.763	1.755	1	.185	.364	.082	1.623
	Birthyear_decades(3)	-1.645	.907	3.292	1	.070	.193	.033	1.141
	Birthyear_decades(4)	-2.162	1.166	3.436	1	.064	.115	.012	1.132
	Birthyear_decades(5)	-16.114	1970.362	.000	1	.993	.000	.000	.
	Constant	-5.576	.692	64.891	1	<.001	.004		

a. Variable(s) entered on step 1: Cluster modal, fodelselandmammapappa_ny, utbildningmammapappa_ny, Birthyear_decades.
